# Curcumin mimics of potential chemoprevention with NQO1 induction properties

**DOI:** 10.1038/s41598-025-85588-w

**Published:** 2025-01-17

**Authors:** Dalia R. Aboshouk, Ahmed R. Hamed, Siva S. Panda, Mohamed S. Bekheit, M. Adel Youssef, Adel S. Girgis

**Affiliations:** 1 Department of Pesticide Chemistry, National Research Centre, Dokki, 12622 Giza Egypt; 2 Chemistry of Medicinal Plants Department, National Research Centre, Dokki, 12622 Giza Egypt; 3 Department of Chemistry & Biochemistry and Department of Biochemistry and Molecular Biology, Augusta University, Augusta, GA 30912 USA; 4 Department of Chemistry, Faculty of Science, Helwan University, Helwan, Egypt

**Keywords:** Cancer, Chemoprevention, Curcumin mimic, Piperidone, NQO1, iNOS, Molecular modeling, Drug delivery, Small molecules

## Abstract

Chemoprevention is one of the accessible strategies for preventing, delaying or reversing cancer processing utilizing chemical intervention of carcinogenesis. NAD(P)H quinone oxidoreductase 1 (NQO1) is a xenobiotic metabolizing cytosolic enzyme/protein with important functional properties towards oxidation stress, supporting its ability in detoxification/chemoprotective role. A set of 3,5-diylidene-4-piperidones (as curcumin mimics) bearing alkyl sulfonyl group were synthesized with potential NQO1 induction properties. Compounds **5ab** (R = 2-MeOC_6_H_4_, R’ = Me) and **5ac** (R = 2-MeOC_6_H_4_, R’ = Et) are the most promising agents synthesized (% induction of NQO1 = 51.562, 45.793) relative to that of 4’-bromoflavone (4’-BF, reference standard) at 10 µM. LPS-induced iNOS production in RAW264.7 macrophages of the most promising agents discovered (**5ab** and **5ac**) displayed concentration-dependent with comparable activities to the reference anti-inflammatory drug indomethacin. Molecular modeling studies (including QSAR, molecular docking and molecular dynamics) were accessed supporting the observed biological profiles.

**Supplementary Information:**

The online version contains supplementary material available at 10.1038/s41598-025-85588-w.

## Introduction

Cancer is one of the top humanity struggled fatal diseases^1^. Chemoprevention is one of the accessible strategies for preventing, delaying or reversing cancer processing^2^. Carcinogenesis is a multi-step complicated process due to either exogenous and/or endogenous agents affecting cellular metabolism and leads to cancer^3^. Chemoprevention is of three stages. Blocking the de novo malignancy, that is considered the preliminary/first stage of cancer prevention. Delaying and/or preventing the progression of the pre-malignant lesions, is the second prevention stage. Prevention of cancer recurrence (metastasis) in cured patients from previously treated diseases is the third stage. Although the first/preliminary approach is preferable to avoid exposure to pain and symptoms accompanied with malignancy, the third approach seems of high interest/need due to difficulties for curing metastasis and also the elevated mortality rates^4^.

Oxidative stress can carry out the canonical activation of Nrf2 (nuclear factor-erythroid2-related factor 2), which requires several steps. First, the Nrf2-Keap1 (Kelch-like ECH-associated protein 1) complex is disrupted by modifications to Keap1’s reactive cysteine residues, such as oxidation or covalent modification by electrophiles. This disruption prevents Nrf2 degradation and promotes its accumulation. Second, nuclear import proteins facilitate the translocation of Nrf2 in the nucleus. Once inside the nucleus, Nrf2 forms heterodimers with small musculoaponeurotic fibrosarcoma (Maf) proteins, another type of transcription factor. This heterodimerization strengthens Nrf2 binding to the antioxidant response element, promoting gene transcription. Third, the Nrf2-Maf complex recruits, activates, and interacts with the basal transcriptional machinery, triggering the transcription of a number of chemopreventive genes, including NAD(P)H quinone oxidoreductase 1 (NQO1), heme oxygenase-1 (HMOX-1), and glutamate-cysteine ligase (GCL)^5^. These genes produce proteins that aid in cellular detoxification, in addition to antioxidation, and redox homeostasis. Chemopreventive genes can be upregulated with structurally-diverse natural and synthetic compounds. Additionally, chemopreventive agents can activate detoxification metabolizing phase II enzymes in particular, including UDP-glucuronosyltransferase, NQO1 and glutathione *S*-transferase^4,6^.

Inducers of chemopreventive genes can be classified into direct, indirect and bifunctional antioxidants. Some hydroxyl phenolic, thiols and Michael acceptors such as olefins or acetylenes connected to carbonyl or electron-withdrawing groups are identified as indirect antioxidants. They are able to induce cytoprotective (phase II) enzymes giving rise to upregulation of many cytoprotecive gene transcription factors^7^. NQO1 is a xenobiotic metabolizing cytosolic enzyme/protein with important functional properties towards oxidation stress. The ability of NQO1 to defend against either exogenous or endogenous quinones through the reduction to less toxic hydroquinones was reported, emphasizing its detoxification/chemoprotective role^8,9^. Hydroquinones are usually unstable and can be turned back to their precursor/parent oxidized forms. During this cycle, many reactive oxygen species (ROS) were formed giving rise to cancer cellular apoptosis^10,11^. Many *p*-quinone, *o*-quinone and non-quinone (of which, coumarins, flavonoids, indolequinone, quinolines, quinazolines, and curcumin) containing compounds were identified as NQO1 substrates with potential anticancer properties^10–16^. The role of NQO1 against free radicals and stabilization of important cellular regulators such as p53 (apoptosis regulator) were also evidenced^17,18^. The overexpression of NQO1 in the early stage of carcinogenesis directed attention towards usefulness in diagnosis of some solid cancers^19–21^. Moreover, compounds capable in regulating NQO1 were also reported as neuroprotective agents useful against Alzheimer’s^22–25^, Parkinson’s diseases^26^ and cerebral ischemic injury^27^.

**Fig. 1:**
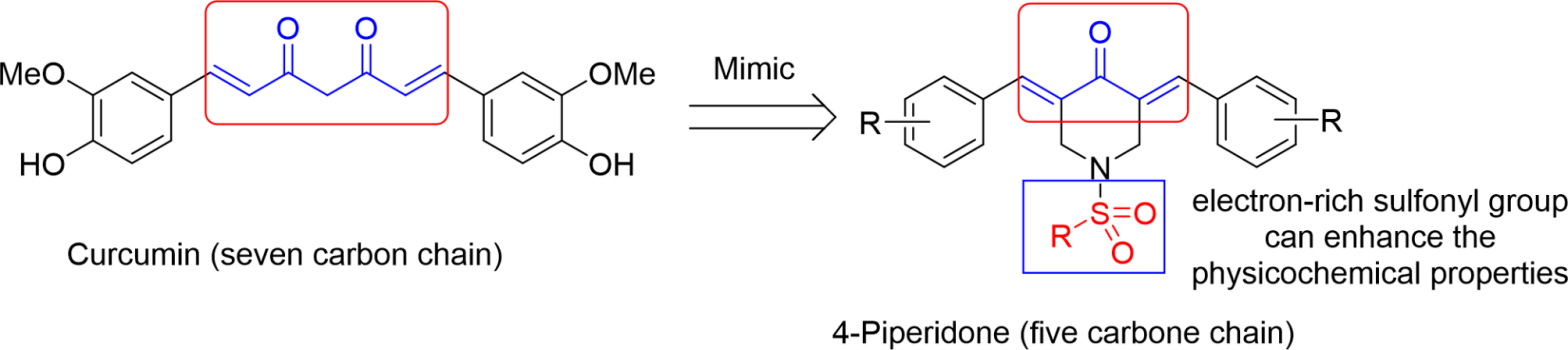
Design of the targeted 1-sulfonyl-3,5-ylidene-4-piperidones (curcumin mimics) with NQO1 induction properties.

The current work deals with synthesis and NQO1 induction investigation of a variety of 3,5-diylidene-4-piperidones. Interest in these compounds (scaffold) is originated from the fact that, these analogs may be recognized as curcumin mimics. Where, the seven carbon chain of curcumin is turned into a five carbon chain via removing the methylene group (responsible for keto-enol isomerization) and one of the ketonic groups. The sulfonyl group linked to the piperidinyl nitrogen is an electron-rich function can enhance the physicochemical properties (Fig. 1). The reported NQO1 induction properties of curcumin^28^ and Michael acceptor-containing compounds with olefinic/unsaturated linkage or electron withdrawing function^7,29,30^ add good support for the hypothesis of the current design/study.

The robust association between inflammation and cancer is now well established^31,32^. The predominant cause of human malignancies is chronic inflammation that drives every stage of carcinogenesis. Activated inflammatory-immune cells in inflamed tissues, such as macrophages and natural killer cells, generate ROS and/or reactive nitrogen species, which can inflict DNA damage and trigger carcinogenesis by activating oncogenes and/or inactivating tumor suppressor genes^33^. Chronic inflammatory tissue damage alters the production and functions of proteins that regulate intracellular signal transduction pathways, as well as structurally modifying DNA or activating carcinogens^34^. Due to all the above mentioned, the current study also includes anti-inflammatory properties investigation of the most promising analogs discovered.

In-silico studies including QSAR (quantitative structure-activity relation), molecular docking, and dynamic simulation will also been considered. Detection of the key descriptor(s) essential for bio-properties in QSAR study can identify the rules/parameters necessary for the efficacy, and optimizing/developing newer hits/leads. Molecular docking is a useful technique for determining the key functional group(s) giving interaction with the lead amino acid(s) of the protein active site controlling the bio-chemical interaction. Molecular dynamic simulation studies are accessible tools for supporting the stability of an effective agent discovered during docking process in the protein active site.

## Results and discussion

### Chemical synthesis

**Fig. 2:**
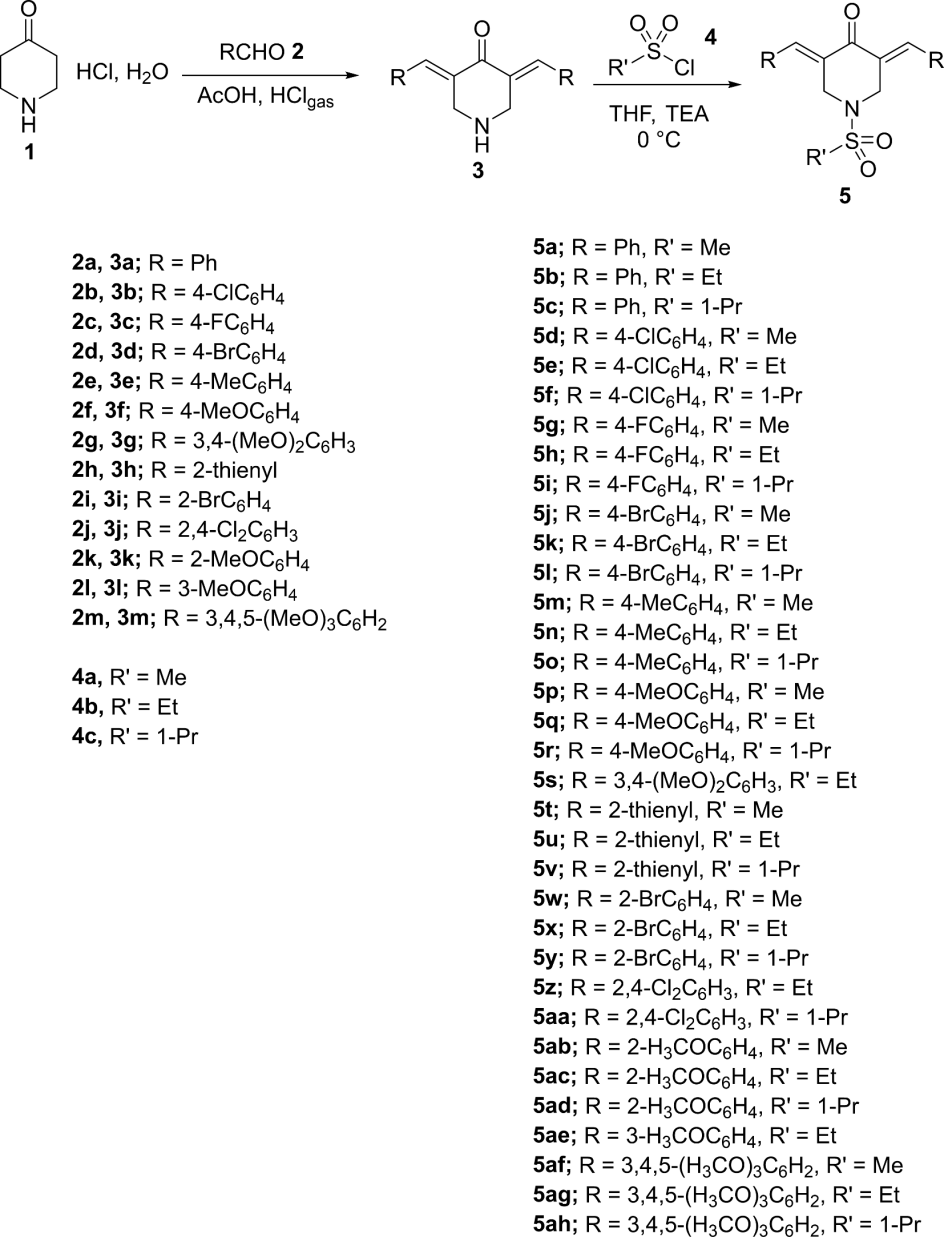
Synthetic route towards **5a-5ah**.

The targeted 1-sulfonyl-3,5-diylidene-4-piperidinones **5a‒5ah** were obtained via direct sulfonylation of the appropriate 3,5-diylidene-1-piperidones **3a‒3m** with the corresponding sulfonyl chlorides **4a‒4c**, as depicted in Fig. 2 (in dry THF “tetrahydrofuran” containing TEA “triethylamine” as basic catalyst for the formed HCl removal/abstraction at 0 °C)^35^. Compounds **3a‒3m** were synthesized through condensation of the appropriate aromatic aldehyde **2a‒2m** with 4-piperidone hydrochloride mono-hydrate **1** in glacial acetic acid using dry HCl_gas_ as dehydrating agent^35–40^. Different spectroscopic technique (IR, 1H- and 13C-NMR) observations support the chemical structure of the synthesized agents (Supplementary material Figs. S1‒S36). The unsaturated carbonyl group was shown at ν = 1667–1678 cm^− 1^, δ_C_ = 184.5‒184.9. The alkylsulfonyl group was revealed at δ_H_ = 3.00‒3.06; 1.15‒1.22, 3.10‒3.20; 0.93‒0.97, 1.57‒1.74, 3.04‒3.17 and at δ_C_ = 35.8‒36.5; 7.4‒7.7, 44.1‒44.8; 12.6‒12.7, 16.3‒16.6, 50.8‒51.7 for the methyl, ethyl, and propyl groups, respectively. The piperidinyl methylene groups were shown at δ_H_ = 4.49‒4.73 and at δ_C_ = 46.0‒46.9. The *E*-configuration was assigned due to the singlet signal at δ_H_ = 7.73‒7.97^41,42^.

### Biological studies

#### NQO1 induction properties

The pre-synthesized piperidones **5a‒5v** with safe behavior against normal cells^35^ were subjected for NQO1 induction assay utilizing the standard technique at 10 µM considering 4’-bromoflavone (4’-BF) as a standard reference^43,44^. From the observed results (Table 1, Supplementary Fig. S37) it noted that, some of the tested agents revealed considerable NQO1 induction properties. Compound **5s** [R = 3,4-(MeO)_2_C_6_H_3_, R’ = Et] was the most promising agent observed (% induction of NQO1 = 45.694). Compounds **5d** (R = 4-ClC_6_H_4_, R’ = Me) and **5e** (R = 4-ClC_6_H_4_, R’ = Et) also exhibited close bio-observations (% induction of NQO1 = 45.264, 42.215, respectively).

SAR (structure-activity relationship) evidenced that, the ethylsulfonyl-containing analogs of 3,5-bis(halogenated benzylidene)-4-piperidones are of enhanced NQO1 induction than the other alkylated (methyl, propyl) analogs investigated (compounds **5e** is an exception). Additionally, the halogenated benzylidene-containing analogs have better bio-observations than the methylbenzylidene-containing compounds (compound **5i** is an exception). The methoxybenzylidene-containing piperidones also have enhanced bio-properties relative to the methylbenzylidene-containing analogs.

**Fig. 3:**
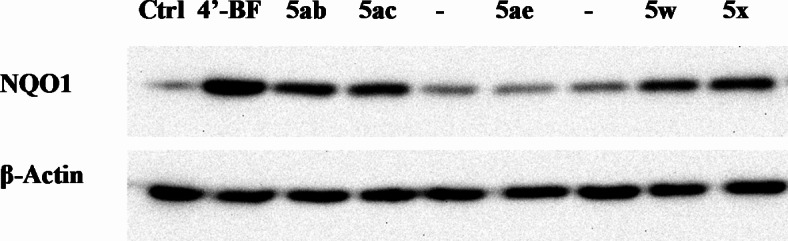
Western blotting of NQO1 induction by the synthesized piperidones **5w**, **5x**, **5ab**, **5ac** and **5ae**. Hepa1c1c7 cells were treated for 48 h with vehicle (0.1% DMSO) or 10 µM of the tested compounds. 4’-BF was used as reference NQO1 inducer. Cell lysates were prepared and NQO1 expression was detected as mentioned in the experimental section.

Based on all the above observations novel curcumin mimics **5w‒5ah** were synthesized and subjected for bio-properties investigation. Firstly, safety of the synthesized agents was investigated at 10 µM adopting the standard technique^43,44^. Due to the revealed safety behavior, compounds **5w**, **5x**, **5ab**, **5ac**, and **5ae** were considered for NQO1 induction properties investigation. From the exhibited results (Fig. 3, Table 2), it is noticed that compound **5ab** (R = 2-MeOC_6_H_4_, R’ = Me) is the most promising agent synthesized (% induction of NQO1 = 51.562). Also, compound **5ac** (R = 2-MeOC_6_H_4_, R’ = Et) showed promising properties (% induction of NQO1 = 45.793). These observations are consistent with the SAR shown by the investigated training set supporting the role of alkyl group of sulfonyl function and also the effect of methoxybenzylidene ring in optimizing bio-active agents.

**Fig. 4:**
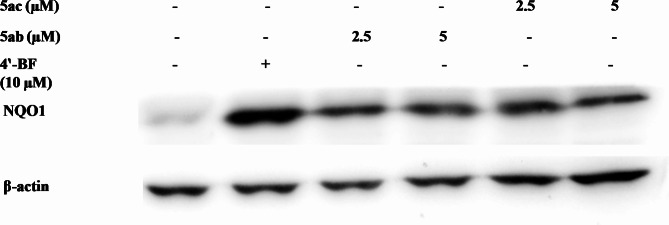
Western blotting showing NQO1 induction by 2.5 and 5 µM of compounds **5ab** and **5ac**.

**Fig. 5:**
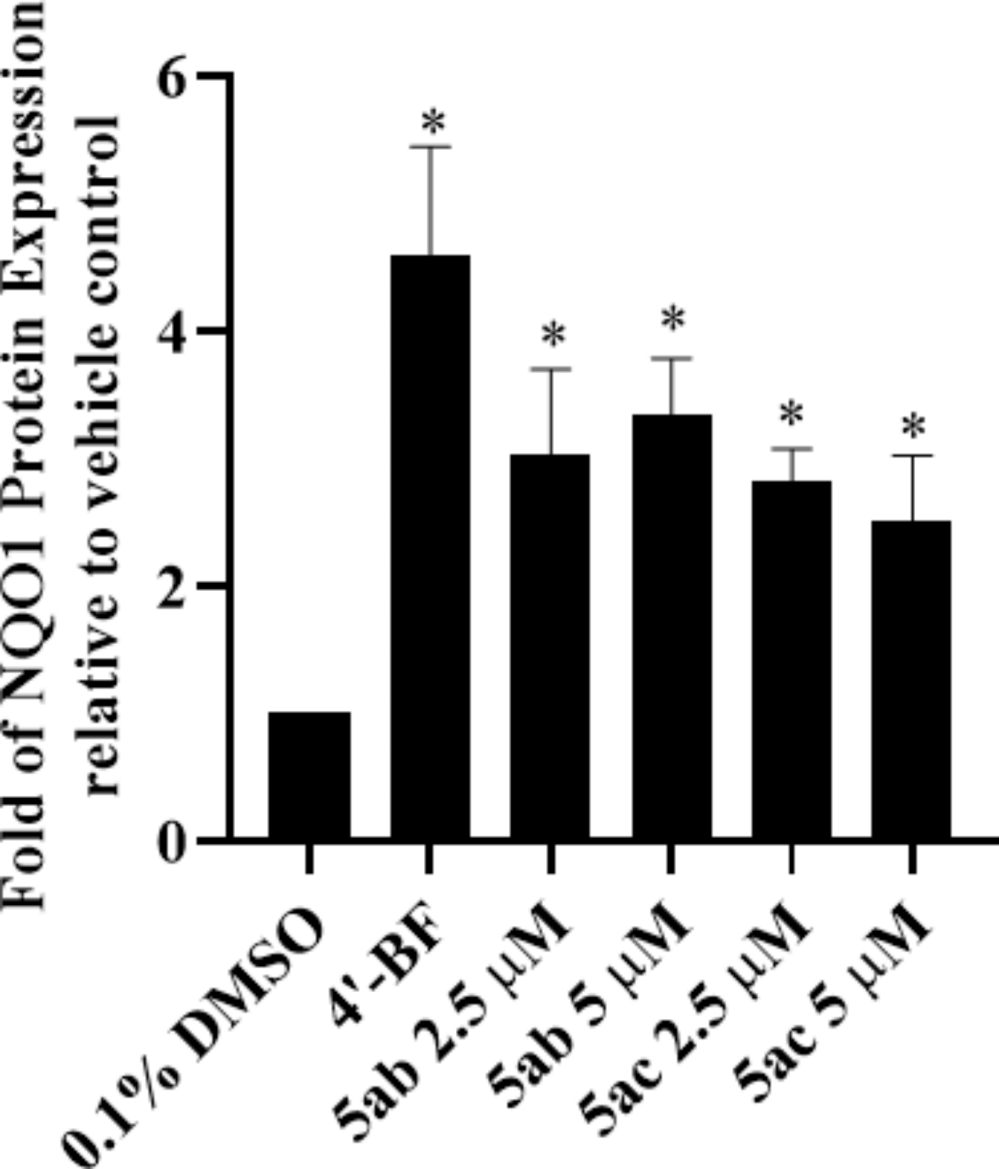
Densitometric analysis of the observed NQO1 induction of protein expression by compounds **5ab** and **5ac** at 2.5 and 5 µM.

As compounds **5ab** and **5ac** revealed the highest NQO1 induction properties among the tested compounds (Tables 1 and 2), we have re-tested them with Western blotting technique at lower concentrations to reveal their concentration-dependent potential. As shown in Fig. 4 (Western blotting) and Fig. 5 (densitometric analysis), considerable induction was observed by both compounds at 2.5 and 5 µM (fold of NQO1 protein induction expression relative to vehicle = 3.0 ± 0.6, 3.3 ± 0.4; 2.8 ± 0.2, 2.5 ± 0.5 for compounds **5ab** and **5ac** at 2.5 and 5 µM, respectively).

**Table 1:** Densitometric analysis showing fold of NQO1 induction properties of the tested compounds **5a‒v** relative to control.

Compd.	Fold induction relative to control	% induction of NQO1 relative to 4’-BF
**5a**	1.044	32.574
**5b**	1.113	34.741
**5c**	0.972	30.343
**5d**	1.450	45.264
**5e**	1.352	42.215
**5f**	1.078	33.661
**5 g**	1.173	36.609
**5 h**	1.204	37.577
**5i**	0.869	27.123
**5j**	1.020	31.822
**5k**	1.182	36.880
**5 L**	1.017	31.752
**5 m**	0.944	29.461
**5n**	0.913	28.483
**5o**	0.960	29.956
**5p**	1.068	33.331
**5q**	1.059	33.045
**5r**	1.222	38.157
**5s**	1.464	45.694
**5t**	1.020	31.827
**5u**	0.934	29.161
**5v**	1.102	34.394
**4’-BF**	3.204	100

**Table 2:** Densitometric analysis showing fold of NQO1 induction properties of the tested compounds **5w**, **5x**, **5ab**, **5ac**, **5ae** and 4’-BF relative to control.

Compd.	Fold induction relative to control	% induction of NQO1 relative to 4’-BF
**5w**	2.777	33.238
**5x**	3.116	37.295
**5ab**	4.308	51.562
**5ac**	3.826	45.793
**5ae**	1.603	19.186
**4’-BF**	8.355	100

#### Anti-inflammatory inhibition of LPS-induced NO production & iNOS protein expression

**Fig. 6:**
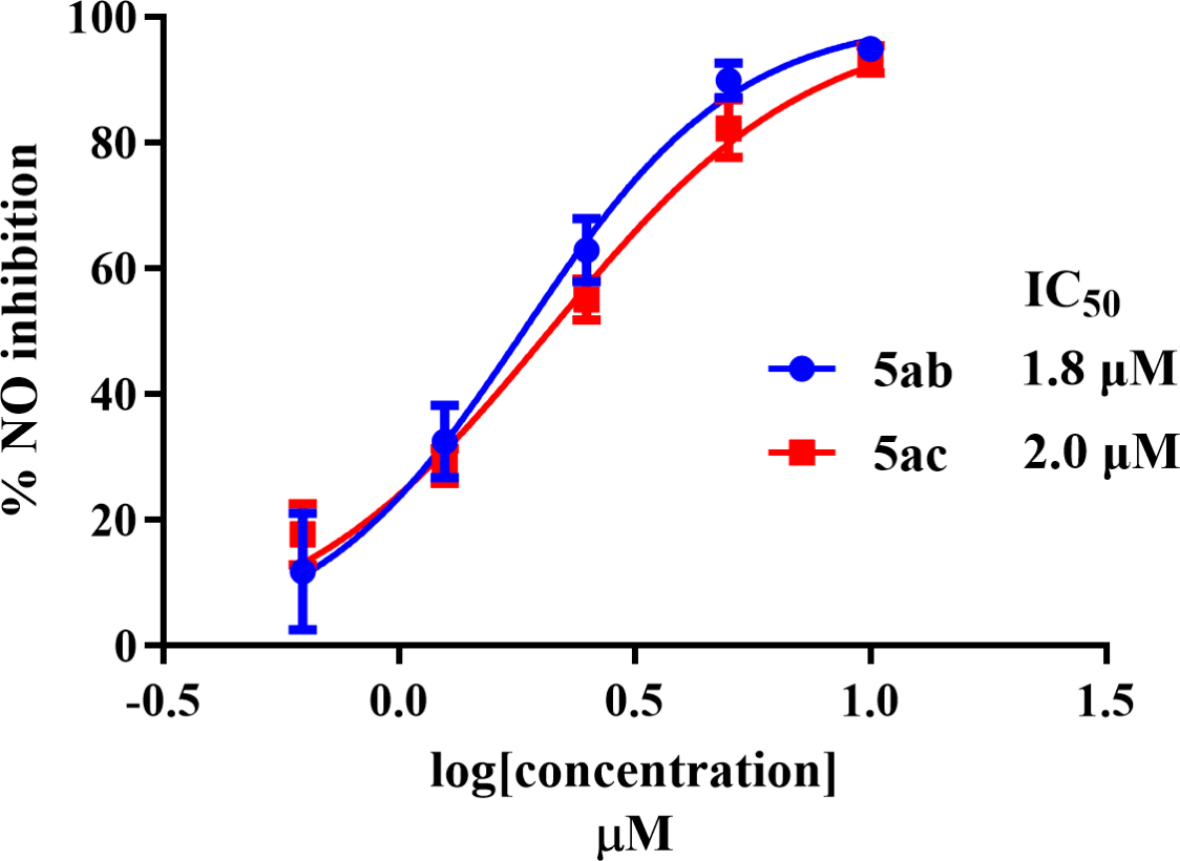
Concentration-dependent inhibition of LPS-induced NO production by **5ab** and **5ac** in RAW264.7 macrophages.

As a strong linkage exists between inflammation and cancer, we tested the anti-inflammatory activity of the promising compounds discovered **5ab** and **5ac**, we employed RAW264.7 macrophages to test for the inhibition of lipopolysaccharide (LPS)–induced NO production by those compounds^43,45^. A strong concentration-dependent inhibition of LPS-induced NO production by **5ab** and **5ac** was revealed. Figure 6 displays the concentration-dependent inhibition recorded. The calculated IC_50_ values of NO inhibition by **5ab** and **5ac** were 1.8 and 2.0 µM, respectively.

**Fig. 7:**
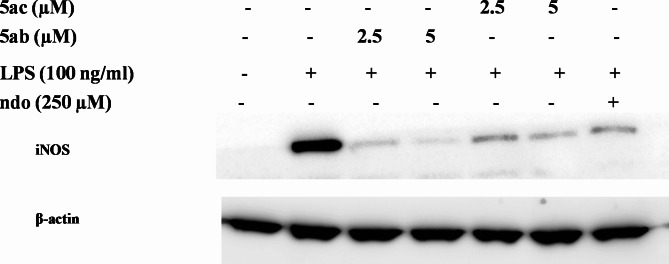
Western blot showing concentration-dependent inhibition of LPS-induced iNOS expression by **5ab** and **5ac** in RAW264.7 macrophages.

**Fig. 8:**
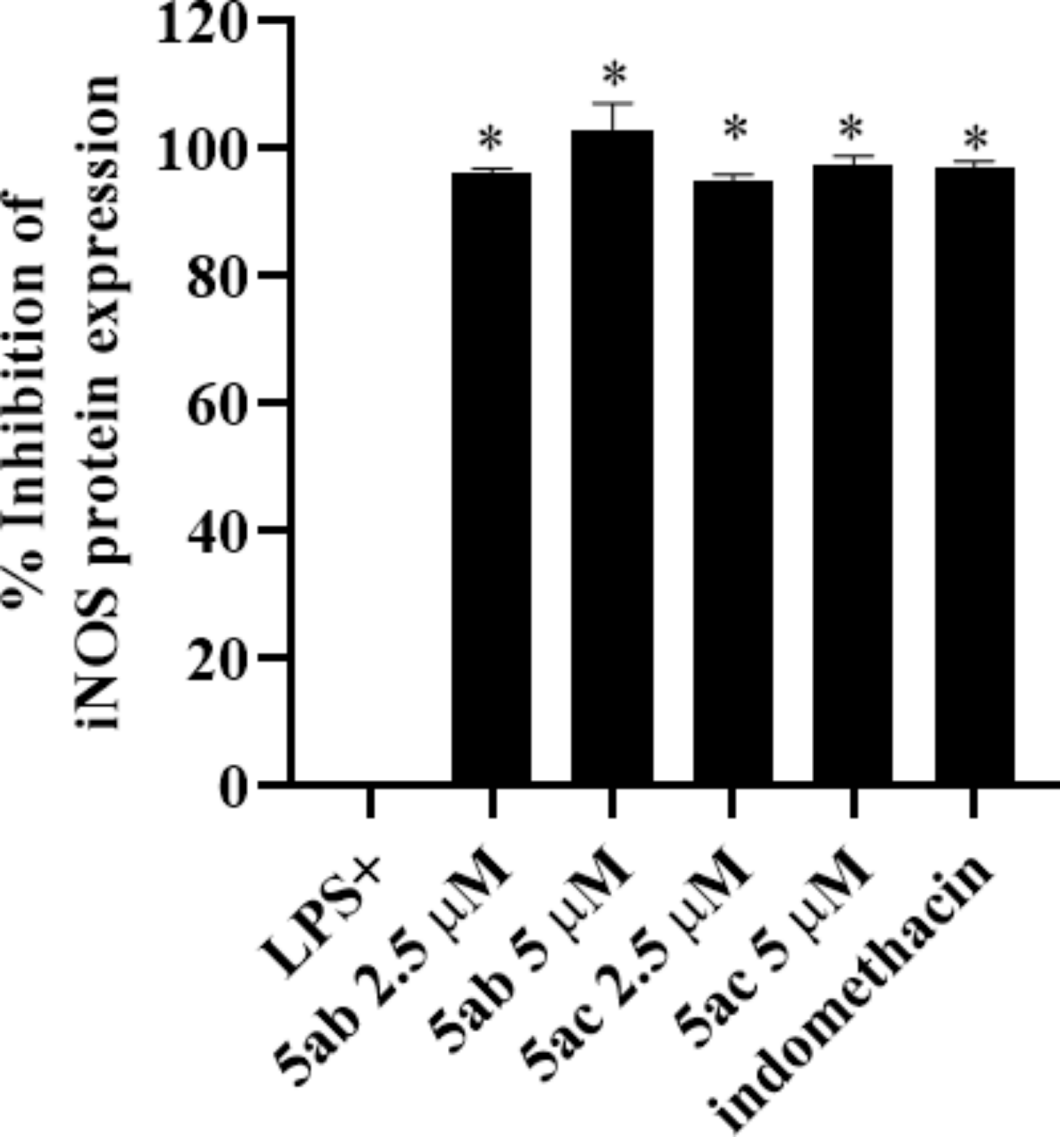
Densitometric analysis of Western blot of LPS-induced iNOS protein expression by **5ab** and **5ac** in RAW264.7 macrophages.

The observed NO inhibitory properties encouraged further investigation of the inhibition of protein expression of the nitric oxide precursor enzyme, inducible nitric oxide synthase (iNOS) using Western blotting^44,46^. The results revealed that both tested compounds showed inhibition of the LPS-induced protein expression of iNOS in RAW264.7 macrophages, The inhibition was concentration-dependent and comparable to the reference anti-inflammatory drug indomethacin (Indo) (% inhibition of LPS-induced iNOS protein production = 96.0 ± 0.8, 100 ± 4.0; 94.5 ± 1.0, 97.1 ± 0.8 for compounds **5ab** and **5ac** at 2.5 and 5 µM, respectively) (Figs. 7 and 8).

### In-silico studies

#### 2D-QSAR studies

The bio-properties can be computationally expressed in mathematical equations expressing the estimated efficacies/potencies in terms of descriptors (physico-chemical) parameters, capable to identify the items governing the biological observations^47,48^. A set of 25 tested compounds (**5a‒n**, **5p‒r**, **5t‒x**, **5ab**, **5ac** and **5ae**) were considered for QSAR modeling studies (training set analogs) utilizing CODESSA-Pro software. However, compounds **5o** and **5s** (representing promising and potent agents) were considered as the test set^49,50^. Five descriptor robust model was optimized due to the training set analogs (homogeneous/non-diverse approach, *N* = 25, *n* = 5, *R*^2^ = 0.836, *R*^2^cvOO = 0.831, *R*^2^cvMO = 0.999) covering a range of % NQO1 induction_(observed)_ = 19.186‒51.562 and % NQO1 induction_(predicted)_ = 22.830‒48.604 (QSAR model/equation is mentioned in the experimental section, Supplementary Tables S1–S3, Fig. S38).

Interaction for N-S bond is a semi-empirical descriptor with highest value of criterion (*t* = 5.996) among the other QSAR model’s descriptors and coefficient value (0.541). The appearance of this descriptor as one of the most important parameter of QSAR model evidenced the importance of sulfonyl group in controlling biological properties which is one of the main elements for the designed molecules. Little differences due to this descriptor value of the training set analogs were noted however, these seem satisfactory for fair exhibition of the predicted biological behavior as shown in compounds **5u** and **5ab** of estimated (% NQO1 induction) values = 26.855, 27.263, due to descriptor value = 28.196, 48.604, respectively. The total energy due to interaction between two atoms can be calculated by equ. (S1)^51^.

Valency for atom H is an atomic type descriptor identifying the type of hybridization as well as the aliphatic relative to aromatic properties of the molecule. Again, little differences were observed due to descriptor values of the training set analogs. Meanwhile, the high coefficient value of the descriptor (3.80646) among the other model’s descriptors justified the predicted properties as shown in compounds **5j** and **5ab** (descriptor value = 0.799, 0.861; estimated % induction of NQO1 = 34.325, 48.604, respectively). Equ. (S2) can calculate the free valence^51^.

Atomic state energy is a semi-empirical descriptor. The coefficient value is of negative sign so, the higher mathematically descriptor value, the lower estimated bio-properties as shown in compounds **5i**/**5ab** (descriptor value = 310.572/310.555, estimated % induction of NQO1 = 26.389/48.604, respectively).

Exchange energy of C-S bond is also a semi-empirical descriptor with a negative coefficient value (-2.024) in the QSAR model. This can justify the estimated bio-properties of the training set analogs **5p**/**5ac** (descriptor value = 3.321/3.200, estimated % induction of NQO1 = 32.350/47.920, respectively). Equ. (S3) can calculate the electronic exchange energy for two atoms^51^.

Relative number of H atoms is a constitutional descriptor with negative sign of coefficient value (-3.112). This explains the predicted % induction of NQO1 of training set compounds **5n**/**5w** (descriptor value = 0.472/0.386, estimated property = 29.782/32.885, respectively). Additionally, a good explanation was also attained due to this descriptor for the lower bio-properties of the 1-propylsulfonyl containing analogs relative to those with ethyl function.

Internal validation of the QSAR model was established due to the comparative values of both cross-validation leave-one-out (*R*^2^cvOO) and leave-many-out (*R*^2^cvMO) coefficient values relative to that of the original coefficient value of the QSAR model (*R*^2^ = 0.836, *R*^2^cvOO = 0.831, *R*^2^cvMO = 0.999). The statistical values (*F* “Fisher criteria” = 19.362, *s*^2^ “standard deviation” = 0.001) are also good signs for the goodness of the QSAR model. The comparable estimated properties to that of the experimental values also support the success of the attained model.

External validation was achieved via utilization of compounds **5o** and **5s** (representing promising and potent agents). The estimated values (% induction of NQO1 = 22.600, 40.808) are close to the experimentally observed values (29.956, 45.694, respectively) adding good evidence for the goodness of the attained QSAR model (Supplementary Table S3).

#### Docking studies

Docking studies of the most promising agents discovered (**5ab** and **5ac**) were undertaken by Discovery Studio 4.1 software (standard CDOCKER technique, utilizing PDB: 4IQK, resolution: 1.97 Å, RMS gradient: 0.0897)^52–54^. NQO1 (NAD(P)H quinone oxidoreductase 1) is one of the important Nrf2 can reduce the reactive quinones capable on oxidative stress so, its induction exhibits detoxification and reveals other functions where, cancer prevention is one of them^8,17,55^.

Docking observations of compound **5ab** in the active site of 4IQK reveal hydrogen bonding of sulfonyl oxygen with ARG415. Non-bonding interactions were also noted by the ylidene phenyl rings including π‒π interaction with TYR334, TYR572 and TYR525 in addition to π-cation interaction with ARG415. Needless to say that, the interacted amino acids revealed are the lead ones giving interactions with the co-crystallized ligand in the active site of 4IQK.

**Fig. 9:**
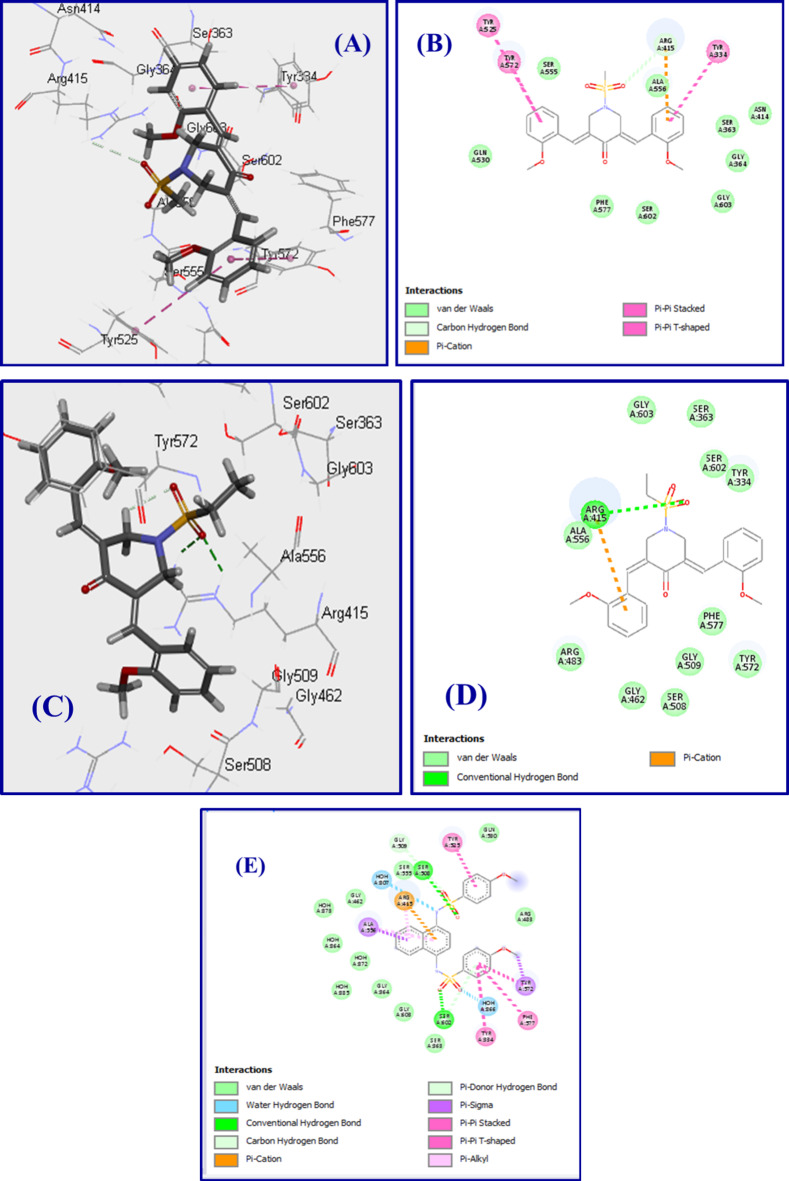
(**A**), (**B**) docking poses (3D and 2D) of **5ab**; (**C**), (**D**) docking poses (3D and 2D) of **5ac**; (**E**) docking pose (2D) of the co-crystallized ligand in the active site of PDB: 4IQK. Figs were obtained due to docking studies and drawn by Biovia Discovery Studio Visualizer (https://discover.3ds.com/discovery-studio-visualizer-download).

Docking observations of **5ac** is similar to that of **5ab**, exhibiting two hydrogen bonding of the sulfonyl oxygen with ARG415 and one non-bonding (π-cation) interaction taking place between phenyl group and ARG415. CDOCKER interaction energy score of compound **5ac** is slightly higher than that of **5ab** (‒35.836, ‒38.206 kcal mol^− 1^ for **5ab** and **5ac**, respectively). The slight differences between the experimentally revealed % induction of NQO1 for **5ab** and **5ac** (51.562, 45.793, respectively) and the estimated properties by 2D-QSAR (48.604, 47.920, respectively), can be correlated to the condition differences between the techniques/methodologies applied (Table 3; Fig. 9).

**Table 3:** CDOCKER interaction energy scores (‒kcal mol^− 1^), hydrogen bonding and non-bonding of the compounds **5ab** and **5ac** in the active site of PDB: 4IQK.

Compd.	Docking score (‒kcal mol^− 1^)	Hydrogen bonding	Non-bonding interaction	
			*π-π interactions*	*π-cation interaction*
**5ab**	35.836	sulfonyl S = O … ARG415	phenyl ‒ TYR334, phenyl ‒ TYR572, phenyl ‒ TYR525	phenyl ‒ ARG415
**5ac**	38.206	2 sulphonyl S = O … ARG415	---	phenyl ‒ ARG415

### Molecular dynamic simulation

Molecular docking affords good information about interactions taking place between a specific ligand/tested agent and amino acids of the protein active site. However, the attained information lacks supporting elements about the stability of ligand-receptor binding interactions. For this purpose, molecular dynamic simulation studies were considered by Discovery Studio 4.1 that has accessibility for identifying RMSD and RMSF (root mean square deviation and fluctuation, respectively). This technique gives good information about flexibility of protein structure within the simulation time and stability taking place of the docked ligand in the protein active site^56–58^. The poses of compounds **5ab** and **5ac** revealing best docking observations in the protein active pocket of PDB: 4IQK were utilized in molecular dynamic simulation studies for assigning the binding stability taking place between the tested agent and protein beside the interaction persistence within the simulation period/time^57^. RMSD can afford good indications about the stability of ligand’s confirmations during molecular dynamic simulation process. Meanwhile, RMSF can determine the volatility of each receptor’s amino acid within the applied simulation process^57,58^.

**Fig. 10:**
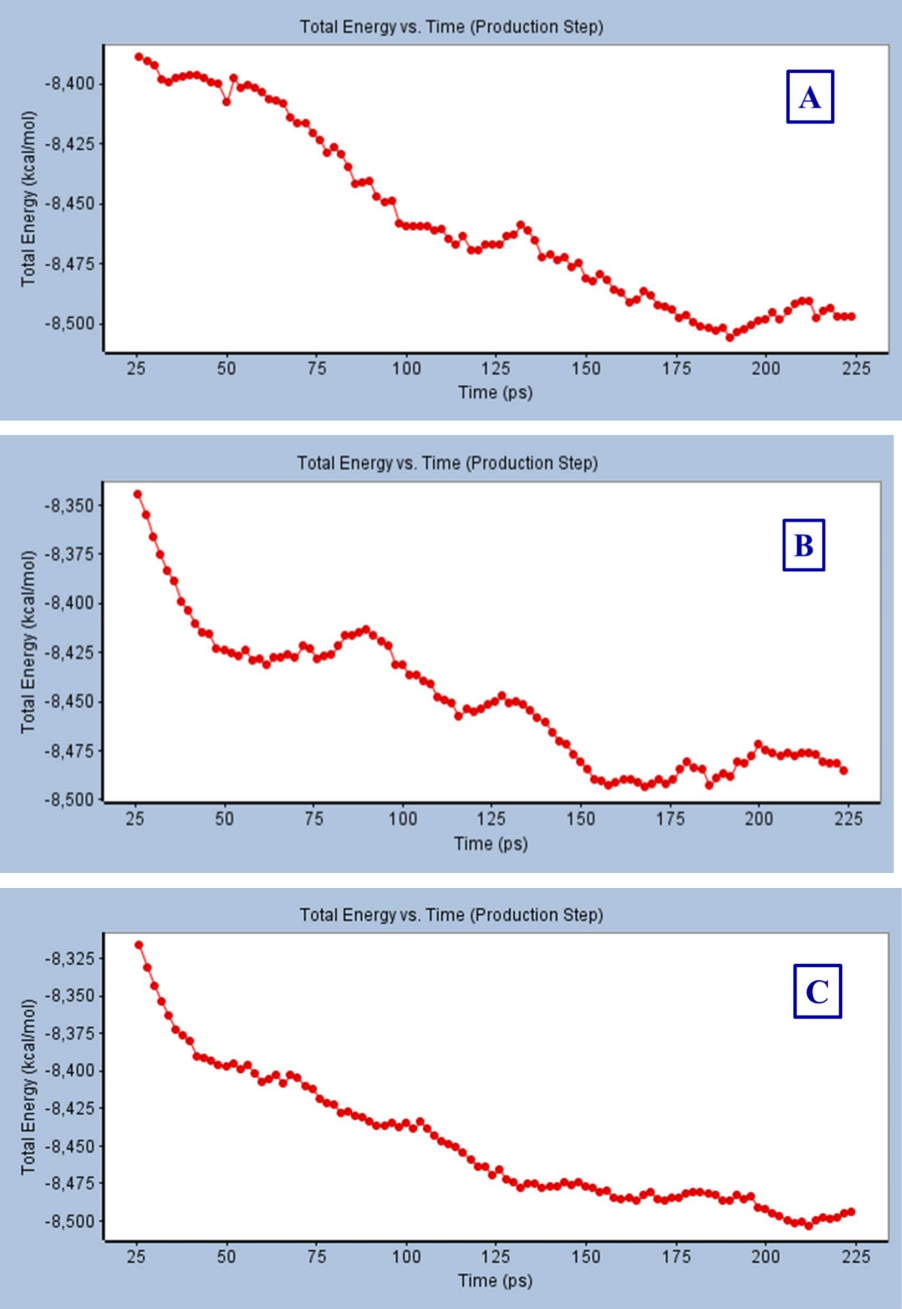
Total energy vs. time in production step during interaction of; (**A**): protein of PDB: 4IQk, (**B**): best conformation pose of compound **5ab** in PDB: 4IQK, (**C**): best conformations pose of compound **5ac** in PDB: 4IQK.

Figure 10 shows the total energy of compounds’ conformers of **5ab** and **5ac** revealing best docking observations in the active pocket of PDB: 4IQk versus simulation time (ps) of the protein. It noticed that the total energy of compound **5ab** drastically reduced from ‒8345.0 to ‒8431.43 kcal mol^− 1^ within 26–62 ps period of simulation, followed by slight increment in total energy (‒8427.79 to ‒ 8413.90 kcal mol^− 1^) within 64‒90 ps time interval. The gradual decrease of the total energy (‒8416.55 to ‒8457.61 kcal mol^− 1^) was then observed within the time period 92–116 ps, followed by an increment in the total energy (‒8454.04 to ‒8450.41 kcal mol^− 1^) for a short time period (118‒132 ps). Finally, slight decrease of the total energy revealing stability (‒8451.46 to ‒8493.62 kcal mol^− 1^) was observed within the simulation period, 134‒168 ps. On the other hand, the total energy of compound **5ac** decreased from ‒8316.68 to ‒8397.81 kcal mol^− 1^ was shown within 26‒50 ps (i.e. ≈ 81 kcal mol^− 1^ depression which seems similar to that of compound **5ab** “≈ 86 kcal mol^− 1^” within shorter period of time “36, 24 ps for **5ab** and **5ac**, respectively”). Gradual decrease in energy from ‒8399.27 to ‒8477.94 kcal mol^− 1^ was shown within 54‒132 ps followed by slight stability in the total energy (‒8478.6 to ‒8494.49 kcal mol^− 1^) within 138‒224 ps period of simulation time. Similar observations were also noticed for the protein structure of PDB: 4IQk (Fig. 10A). The initial total energy of the tested compounds **5ab** and **5ac** upon dropping their most promising pose observed during molecular docking (-8345.0, -8316.68 kcal mol^− 1^, respectively) drastically decreased within the applied simulation time/period revealing stable conformation with total energy values = -8485.52, -8494.49 mol^− 1^, respectively.

**Fig. 11:**
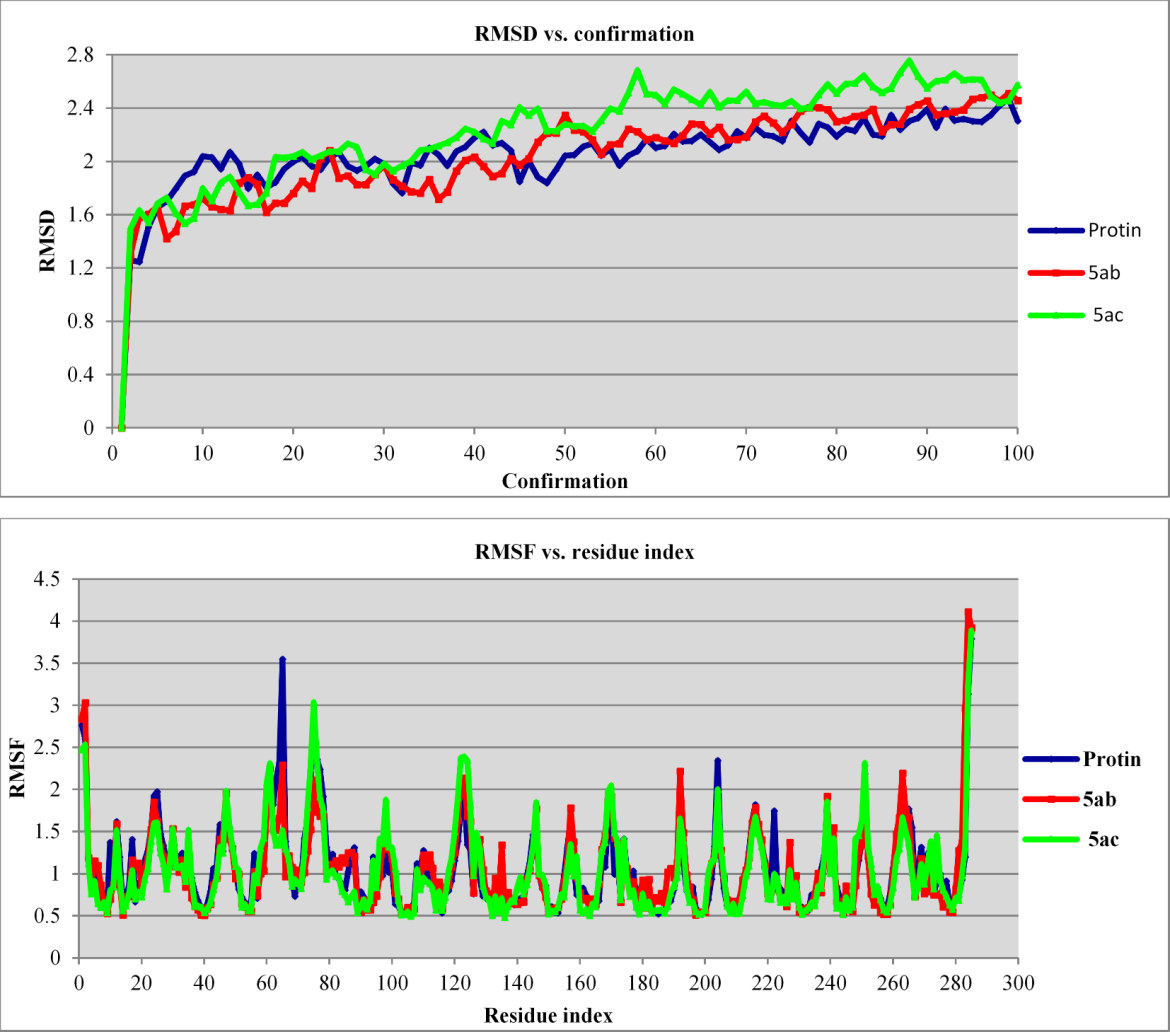
(**A**): RMSD, (**B**): RMSF of the protein and best conformation pose of compounds **5ab** and **5ac** in PDB: 4IQK.

Stability of RMSD of compounds **5ab** and **5ac** was observed for all the conformations detected throughout the molecular dynamic simulation studies with range = 1.293‒2.512 and 1.489‒2.758 Å, respectively, which is a similar behavior to the protein PDB: 4IQk (range = 1.246‒2.470 Å) within the trajectory period (Fig. 11, Supplementary Tables S4‒S6).

RMSF (Fig. 11, Supplementary Tables S7‒S9) is helpful for identifying fluctuations/local changes taking place in protein structure within the trajectory period. It is noticed in compound **5ab** that, high RMSF was shown at residue indexes = 2, 284 and 285 with RMSF = 3.03317, 4.11098 and 3.92339, corresponding to the protein amino acids ARG326, VAL608 and THR609, respectively. Compound **5ac** revealed the highest fluctuation (RMSF) values at residue indexes = 75, 284 and 285 with RMSF values = 3.03753, 3.24464 and 3.88858, corresponding to protein amino acids MET399, VAL608 and THR609, respectively. Another important notice due to mild RMSF of residue index = 91 of RMSF values = 0.690361 and 0.692867 in **5ab** and **5ac**, respectively which corresponds to ARG415. Taking into consideration that, ARG415 is one of the lead amino acids of the protein active site/pocket, revealing interactions with the synthesized/tested agents and co-crystallized ligand (Fig. 9). From all the above studies including RMSD and RMSF, it can be concluded that molecular dynamic simulation supported the stability of docking poses for compounds **5ab** and **5ac** within the trajectory period. In other words these observations evidence the CDOCKER interaction attained with energy values = ‒35.836 and ‒38.206 kcal mol^− 1^ for compounds **5ab** and **5ac**, respectively.

## Conclusion

In conclusion, the synthesized 3,5-diylidene-4-piperidones bearing alkyl sulfonyl group **5** have potential NQO1 induction properties. Compounds **5ab** (R = 2-MeOC_6_H_4_, R’ = Me) and **5ac** (R = 2-MeOC_6_H_4_, R’ = Et) are the most promising agents discovered revealing considerable NQO1 induction observations relative to the standard reference 4’-BF. Anti-inflammatory properties of the most promising agents discovered (**5ab** and **5ac**) were established through LPS-induced iNOS production in RAW264.7 macrophages displaying concentration-dependent comparable to the standard reference drug indomethacin. Molecular modeling studies (including QSAR, molecular docking and molecular dynamics) were accessed supporting the observed biological profiles. Bonding interactions revealed by the sulfonyl group with the lead amino acid (ARG415) of the protein active site during the docking studies evidenced the role of this functional group for bio-observations. QSAR studies also evidenced the importance of this functional group for bio-properties due to the revealed descriptor “interaction for bond N-S” as the top important one controlling QSAR model based on its *t*-value. Although few synthesized analogs showed promising NQO1 induction properties, none revealed potent observations relative to the standard reference used. Further chemical modification and biological exploration based on the bio-properties revealed by the promising agents discovered can assist in optimizing lead active agent(s). Manipulation of the substituent of both sulfonyl group and ylidene linkages may optimize effective agents. Molecular modeling techniques can also assist in optimizing the chemical structure of potent hits.

### Experimental

#### Chemical synthesis

Melting points were determined on a capillary point apparatus (Stuart SMP3) equipped with a digital thermometer. IR spectra (KBr) were recorded on a Shimadzu FT-IR 8400 S spectrophotometer. Reactions were monitored using thin layer chromatography (TLC) on 0.2 mm silica gel F254 plates (Merck) utilizing various solvents for elution. The chemical structures of the synthesized compounds were characterized by nuclear magnetic resonance spectra 1H-, 13C-NMR) and determined on a Bruker NMR spectrometer (500 MHz, 125 MHz for 1H and^ 13^C, respectively)^13^. C NMR spectra are fully decoupled. Chemical shifts were reported in parts per million (ppm) using the deuterated solvent peak or tetramethylsilane as an internal standard. Compounds **3a‒3 m** and **5a‒5v** were obtained through the previously mentioned procedures^35–40^.

**Synthesis of 1-(alkylsulfonyl)-3**,**5-bis(arylmethylene)-4-piperidones (general procedure) 5a‒5ah**.

A solution of the appropriate alkylsulfonyl chloride **4a‒4c** (2.75 mmol in dry tetrahydrofuran (THF, 5 ml), was added dropwise to a solution of the corresponding 3,5-bis(arylmethylene)-4-piperidone **3a‒3m** (2.5 mmol) in dry tetrahydrofuran (20 ml) containing triethylamine (TEA, 2.75 mmol), while stirring at 0 °C (ice bath) within 10 min. Stirring of the reaction at the same condition was continued for 3 h. The reaction was stored in the fridge (5 °C) overnight then evaporated under reduced pressure. The remaining residue was triturated with methanol (5 ml) so; the separated solid was collected and crystallized from a suitable solvent affording the corresponding **5a‒5ah**.

3,5-Bis[(*E*)-2-bromobenzylidene]-1-(methylsulfonyl)piperidin-4-one **5w**.

Obtained from the reaction of **3i** and **4a**, as yellow microcrystals from n-butanol, mp 180–182 °C and yield 91% (1.16 g). IR: *ν*_max_/cm^− 1^ 3063, 2924, 2820, 1674, 1616, 1582, 1462. 1H-NMR (DMSO-*d*_*6*_) *δ* (ppm): 3.00 (s, 3 H, SCH_3_), 4.49 (s, 4 H, 2 NCH_2_), 7.43 (dt, *J* = 1.7, 8.5 Hz, 2 H, arom. H), 7.48, (d, *J* = 7.7 Hz, 2 H, arom. H), 7.54 (t, *J* = 7.5 Hz, 2 H, arom. H), 7.81 (d, *J* = 7.9 Hz, 2 H, arom. H), 7.86 (s, 2 H, 2 olefinic CH). 13C-NMR (DMSO-*d*_*6*_) *δ* (ppm): 36.3 (SCH_3_), 46.3 (NCH_2_), 124.5, 128.0, 130.9, 131.4, 132.3, 133.1, 133.7, 136.0 (arom. C + olefinic C), 184.5 (CO). Anal. Calcd. for C_20_H_17_Br_2_NO_3_S (511.23): C, 46.99; H, 3.35; N, 2.74. Found: C, 47.22; H, 3.56; N, 2.91.

3,5-Bis[(*E*)-2-bromobenzylidene]-1-(ethylsulfonyl)piperidin-4-one **5x**.

Obtained from the reaction of **3i** and **4b**, as yellow microcrystals from n-butanol, mp 157–159 °C and yield 87% (1.14 g). IR: *ν*_max_/cm^− 1^ 3059, 2982, 2928, 1674, 1620, 1585, 1462. 1H-NMR (DMSO-*d*_*6*_) *δ* (ppm): 1.15 (t, *J* = 7.4 Hz, 3 H, CH_3_), 3.10 (q, *J* = 7.4 Hz, 2 H, SCH_2_), 4.53 (s, 4 H, 2 NCH_2_), 7.42 (t, *J* = 7.7 Hz, 2 H, arom. H), 7.47 (d, *J* = 7.7 Hz, 2 H, arom. H), 7.53 (t, *J* = 7.6 Hz, 2 H, arom. H), 7.80 (d, *J* = 8.1 Hz, 2 H, arom. H), 7.84 (s, 2 H, 2 olefinic CH). 13C-NMR (DMSO-*d*_*6*_) *δ* (ppm): 7.4 (CH_3_), 44.6 (SCH_2_), 46.1 (NCH_2_), 124.5, 128.0, 130.9, 131.4, 132.7, 133.1, 133.7, 135.8 (arom. C + olefinic C), 184.7 (CO). Anal. Calcd. for C_21_H_19_Br_2_NO_3_S (525.26): C, 48.02; H, 3.65; N, 2.67. Found: C, 48.21; H, 3.89; N, 2.80.

3,5-Bis[(*E*)-2-bromobenzylidene]-1-(propylsulfonyl)piperidin-4-one **5y**.

Obtained from the reaction of **3i** and **4c**, as yellow microcrystals from methanol, mp 139–141 °C and yield 73% (0.99 g). IR: *ν*_max_/cm^− 1^ 3059, 2970, 2920, 2874, 1678, 1616, 1585, 1462. 1H-NMR (DMSO-*d*_*6*_) *δ* (ppm): 0.93 (dt, *J* = 2.1, 7.5 Hz, 3 H, CH_3_), 1.57‒1.64 (m, 2 H, CH_3_*CH*_*2*_CH_2_), 3.04 (dt, *J* = 2.0, 7.7 Hz, 2 H, S*CH*_*2*_CH_2_), 4.52 (s, 4 H, 2 NCH_2_), 7.42 (dt, *J* = 1.8, 7.7 Hz, 2 H, arom. H), 7.47 (dd, *J* = 1.9, 7.8 Hz, 2 H, arom. H), 7.54 (t, *J* = 7.5 Hz, 2 H, arom. H), 7.81 (d, *J* = 8.2 Hz, 2 H, arom. H), 7.83 (s, 2 H, 2 olefinic CH). 13C-NMR (DMSO-*d*_*6*_) *δ* (ppm): 12.6 (CH_3_), 16.4 (CH_3_*CH*_*2*_), 46.0 (NCH_2_), 51.5 (SCH_2_), 124.5, 128.0, 130.9, 131.4, 132.7, 133.1, 133.7, 135.8 (arom. C + olefinic C), 184.8 (CO). Anal. Calcd. for C_22_H_21_Br_2_NO_3_S (539.28): C, 49.00; H, 3.93; N, 2.60. Found: C, 49.16; H, 3.82; N, 2.74.

3,5-Bis[(*E*)-2,4-dichlorobenzylidene]-1-(ethylsulfonyl)piperidin-4-one **5z**.

Obtained from the reaction of **3j** and **4b**, as pale yellow microcrystals from n-butanol, mp 183–185 °C and yield 78% (0.98 g). IR: *ν*_max_/cm^− 1^ 3071, 2974, 2940, 1674, 1612, 1582, 1466. 1H-NMR (DMSO-*d*_*6*_) *δ* (ppm): 1.15 (t, *J* = 7.3 Hz, 3 H, CH_3_), 3.13 (q, *J* = 7.4 Hz, 2 H, SCH_2_), 4.52 (s, 4 H, 2 NCH_2_), 7.51 (d, *J* = 8.4 Hz, 2 H, arom. H), 7.58 (dd, *J* = 2.1, 8.4 Hz, 2 H, arom. H), 7.80 (s, 2 H, 2 olefinic CH), 7.82 (d, *J* = 2.1 Hz, 2 H, arom. H). 13C-NMR (DMSO-*d*_*6*_) *δ* (ppm): 7.4 (CH_3_), 44.6 (SCH_2_), 46.1 (NCH_2_), 127.7, 129.5, 131.0, 132.0, 132.1, 133.7, 135.0 (arom. C + olefinic C), 184.6 (CO). Anal. Calcd. for C_21_H_17_Cl_4_NO_3_S (505.23): C, 49.92; H, 3.39; N, 2.77. Found: C, 50.06; H, 3.48; N, 2.87.

3,5-Bis[(E)-2,4-dichlorobenzylidene]-1-(propylsulfonyl)piperidin-4-one **5aa**.

Obtained from the reaction of **3j** and **4c**, as pale yellow microcrystals from n-butanol, mp 147–149 °C and yield 79% (1.03 g). IR: *ν*_max_/cm^− 1^ 3071, 2967, 2924, 1674, 1612, 1578, 1466. 1H-NMR (DMSO-*d*_*6*_) *δ* (ppm): 0.93 (t, *J* = 7.4 Hz, 3 H, CH_3_), 1.61 (sextet, *J* = 7.5 Hz, 2 H, CH_3_*CH*_*2*_CH_2_), 3.08 (t, *J* = 7.8 Hz, 2 H, S*CH*_*2*_CH_2_), 4.51 (s, 4 H, 2 NCH_2_), 7.51 (d, *J* = 8.4 Hz, 2 H, arom. H), 7.58 (dd, *J* = 2.2, 8.3 Hz, 2 H, arom. H), 7.80 (s, 2 H, 2 olefinic CH), 7.83 (d, *J* = 2.1 Hz, 2 H, arom. H). 13C-NMR (DMSO-*d*_*6*_) *δ* (ppm): 12.6 (CH_3_), 16.4 (CH_3_*CH*_*2*_), 46.1 (NCH_2_), 51.4 (SCH_2_), 127.7, 129.5, 131.0, 132.0, 132.2, 133.6, 134.97, 135.01 (arom. C + olefinic C), 184.6 (CO). Anal. Calcd. for C_22_H_19_Cl_4_NO_3_S (519.26): C, 50.89; H, 3.69; N, 2.70. Found: C, 50.81; H, 3.57; N, 2.76.

3,5-Bis[(*E*)-2-methoxybenzylidene]-1-(methylsulfonyl)piperidin-4-one **5ab**.

Obtained from the reaction of **3k** and **4a**, as yellow microcrystals from methanol, mp 182–184 °C and yield 84% (0.87 g). IR: *ν*_max_/cm^− 1^ 3005, 2928, 2835, 1674, 1620, 1597, 1485. 1H-NMR (DMSO-*d*_*6*_) *δ* (ppm): 3.00 (s, 3 H, SCH_3_), 3.88 (s, 6 H, 2 OCH_3_), 4.50 (s, 4 H, 2 NCH_2_), 7.07 (t, *J* = 7.5 Hz, 2 H, arom. H), 7.15 (d, *J* = 8.4 Hz, 2 H, arom. H), 7.35 (d, *J* = 7.7 Hz, 2 H, arom. H), 7.48 (t, *J* = 7.9 Hz, 2 H, arom. H), 7.97 (s, 2 H, 2 olefinic CH). 13C-NMR (DMSO-*d*_*6*_) *δ* (ppm): 35.8 (SCH_3_), 46.9 (NCH_2_), 55.7 (OCH_3_), 111.5, 120.4, 122.5, 130.2, 130.6, 131.6, 132.5, 158.1 (arom. C + olefinic C), 184.7 (CO). Anal. Calcd. for C_22_H_23_NO_5_S (413.49): C, 63.91; H, 5.61; N, 3.39. Found: C, 64.04; H, 5.54; N, 3.28.

1-(Ethylsulfonyl)-3,5-bis[(*E*)-2-methoxybenzylidene]piperidin-4-one **5ac**.

Obtained from the reaction of **3k** and **4b**, as yellow microcrystals from methanol, mp 180–182 °C and yield 78% (0.83 g). IR: *ν*_max_/cm^− 1^ 3067, 2943, 2920, 2843, 1670, 1616, 1597, 1485. 1H-NMR (DMSO-*d*_*6*_) *δ* (ppm): 1.18 (t, *J* = 7.4 Hz, 3 H, CH_3_), 3.12 (q, *J* = 7.3 Hz, 2 H, SCH_2_), 3.88 (s, 6 H, 2 OCH_3_), 4.55 (s, 4 H, 2 NCH_2_), 7.07 (t, *J* = 7.5 Hz, 2 H, arom. H), 7.15 (d, *J* = 8.4 Hz, 2 H, arom. H), 7.33 (d, *J* = 7.6 Hz, 2 H, arom. H), 7.47 (dt, *J* = 1.7, 8.7 Hz, 2 H, arom. H), 7.94 (s, 2 H, 2 olefinic CH). 13C-NMR (DMSO-*d*_*6*_) *δ* (ppm): 7.4 (CH_3_), 44.1 (SCH_2_), 46.7 (NCH_2_), 55.7 (OCH_3_), 111.5, 120.4, 122.6, 130.2, 131.1, 131.6, 132.2, 158.1 (arom. C + olefinic C), 184.9 (CO). Anal. Calcd. for C_23_H_25_NO_5_S (427.52): C, 64.62; H, 5.89; N, 3.28. Found: C, 64.77; H, 5.76; N, 3.24.

3,5-Bis[(*E*)-2-methoxybenzylidene]-1-(propylsulfonyl)piperidin-4-one **5ad**.

Obtained from the reaction of **3k** and **4c**, as yellow microcrystals from n-butanol, mp 156–158 °C and yield 72% (0.79 g). IR: *ν*_max_/cm^− 1^ 3075, 3017, 2970, 2936, 2839, 1670, 1601, 1574, 1489. 1H-NMR (DMSO-*d*_*6*_) *δ* (ppm): 0.95 (dt, *J* = 1.8, 7.4 Hz, 3 H, CH_3_), 1.65 (sextet, *J* = 7.5 Hz, 2 H, CH_3_*CH*_*2*_CH_2_), 3.09 (t, *J* = 7.8 Hz, 2 H, S*CH*_*2*_CH_2_), 3.88 (s, 6 H, 2 OCH_3_), 4.54 (s, 4 H, 2 NCH_2_), 7.07 (t, *J* = 7.5 Hz, 2 H, arom. H), 7.15 (d, *J* = 8.4 Hz, 2 H, arom. H), 7.33 (d, *J* = 7.6 Hz, 2 H, arom. H), 7.47 (t, *J* = 8.0 Hz, 2 H, arom. H), 7.94 (s, 2 H, 2 olefinic CH). 13C-NMR (DMSO-*d*_*6*_) *δ* (ppm): 12.7 (CH_3_), 16.3 (CH_3_*CH*_*2*_), 46.6 (NCH_2_), 50.8 (SCH_2_), 55.6 (OCH_3_), 111.5, 120.4, 122.6, 130.2, 131.1, 131.6, 132.2, 158.1 (arom. C + olefinic C), 184.9 (CO). Anal. Calcd. for C_24_H_27_NO_5_S (441.54): C, 65.29; H, 6.16; N, 3.17. Found: C, 65.12; H, 6.11; N, 3.14.

1-(Ethylsulfonyl)-3,5-bis[(*E*)-3-methoxybenzylidene]piperidin-4-one **5ae**.

Obtained from the reaction of **3l** and **4b**, as yellow microcrystals from n-butanol, mp 144–146 °C and yield 71% (0.76 g). IR: *ν*_max_/cm^− 1^ 3063, 2963, 2928, 2835, 1667, 1605, 1574, 1497. 1H-NMR (DMSO-*d*_*6*_) *δ* (ppm): 1.20 (t, *J* = 7.4 Hz, 3 H, CH_3_), 3.19 (q, *J* = 7.4 Hz, 2 H, SCH_2_), 3.83 (s, 6 H, 2 OCH_3_), 4.68 (s, 4 H, 2 NCH_2_), 7.05‒7.12 (m, 6 H, arom. H), 7.42‒7.48 (m, 2 H, arom. H), 7.75 (s, 2 H, 2 olefinic CH). 13C-NMR (DMSO-*d*_*6*_) *δ* (ppm): 7.5 (CH_3_), 44.3 (SCH_2_), 46.6 (NCH_2_), 55.3 (OCH_3_), 115.5, 115.80, 115.89, 115.90, 122.5, 122.6, 128.1, 129.96, 130.0, 131.8, 135.0, 135.4, 136.6, 139.2, 159.37, 159.4 (arom. C + olefinic C), 184.9 (CO). Anal. Calcd. for C_23_H_25_NO_5_S (427.52): C, 64.62; H, 5.89; N, 3.28. Found: C, 64.73; H, 5.95; N, 3.35.

1-(Methylsulfonyl)-3,5-bis[(*E*)-3,4,5-trimethoxybenzylidene]piperidin-4-one **5af**.

Obtained from the reaction of **3m** and **4a**, as yellow microcrystals from n-butanol, mp 163–165 °C and yield 86% (1.15 g). IR: *ν*_max_/cm^− 1^ 3009, 2932, 2839, 1678, 1612, 1578, 1504. 1H-NMR (DMSO-*d*_*6*_) *δ* (ppm): 3.06 (s, 3 H, SCH_3_), 3.75 (s, 6 H, 2 OCH_3_), 3.85 (s, 12 H, 4 OCH_3_), 4.67 (s, 4 H, 2 NCH_2_), 6.85 (s, 4 H, arom. H), 7.75 (s, 2 H, 2 olefinic CH). 13C-NMR (DMSO-*d*_*6*_) *δ* (ppm): 36.5 (SCH_3_), 46.6 (NCH_2_), 56.1, 60.1 (OCH_3_), 108.2, 129.5, 130.5, 137.3, 138.9, 152.9 (arom. C + olefinic C), 184.6 (CO). Anal. Calcd. for C_26_H_31_NO_9_S (533.59): C, 58.53; H, 5.86; N, 2.63. Found: C, 58.41; H, 5.78; N, 2.77.

1-(Ethylsulfonyl)-3,5-bis[(*E*)-3,4,5-trimethoxybenzylidene]piperidin-4-one **5ag**.

Obtained from the reaction of **3m** and **4b**, as yellow microcrystals from n-butanol, mp 155–157 °C and yield 74% (1.01 g). IR: *ν*_max_/cm^− 1^ 3067, 2947, 2828, 1674, 1612, 1582, 1508. 1H-NMR (DMSO-*d*_*6*_) *δ* (ppm): 1.22 (t, *J* = 7.2 Hz, 3 H, CH_3_), 3.20 (q, *J* = 7.4 Hz, 2 H, SCH_2_), 3.75 (s, 6 H, 2 OCH_3_), 3.85 (s, 12 H, 4 OCH_3_), 4.73 (s, 4 H, 2 NCH_2_), 6.85 (s, 4 H, arom. H), 7.73 (s, 2 H, 2 olefinic CH). 13C-NMR (DMSO-*d*_*6*_) *δ* (ppm): 7.7 (CH_3_), 44.8 (SCH_2_), 46.5 (NCH_2_), 56.1, 60.1 (OCH_3_), 108.2, 129.6, 131.0, 136.9, 138.9, 152.9 (arom. C + olefinic C), 184.8 (CO). Anal. Calcd. for C_27_H_33_NO_9_S (547.62): C, 59.22; H, 6.07; N, 2.56. Found: C, 59.02; H, 6.19; N, 2.65.

1-(Propylsulfonyl)-3,5-bis[(*E*)-3,4,5-trimethoxybenzylidene]piperidin-4-one **5ah**.

Obtained from the reaction of **3m** and **4c**, as yellow microcrystals from n-butanol, mp 134–136 °C and yield 70% (0.98 g). IR: *ν*_max_/cm^− 1^ 2970, 2940, 2882, 2839, 1678, 1616, 1582, 1504. 1H-NMR (DMSO-*d*_*6*_) *δ* (ppm): 0.97 (dt, *J* = 1.9, 7.4 Hz, 3 H, CH_3_), 1.67‒1.74 (m, 2 H, CH_3_*CH*_*2*_CH_2_), 3.17 (dt, *J* = 1.8, 7.2 Hz, 2 H, S*CH*_*2*_CH_2_), 3.75 (s, 6 H, 2 OCH_3_), 3.86 (s, 12 H, 4 OCH_3_), 4.71 (s, 4 H, 2 NCH_2_), 6.85 (s, 4 H, arom. H), 7.74 (s, 2 H, 2 olefinic CH). 13C-NMR (DMSO-*d*_*6*_) *δ* (ppm): 12.7 (CH_3_), 16.6 (CH_3_*CH*_*2*_), 46.5 (NCH_2_), 51.7 (SCH_2_), 56.1, 60.1 (OCH_3_), 108.2, 129.6, 131.0, 137.0, 138.9, 152.9 (arom. C + olefinic C), 184.8 (CO). Anal. Calcd. for C_28_H_35_NO_9_S (561.65): C, 59.88; H, 6.28; N, 2.49. Found: C, 59.77; H, 6.21; N, 2.43.

### Biological studies

All the biological studies conducted obey the standards and approved by the Research Ethics Committee, National Research Centre, Egypt (associated with project ID: 13060103).

#### Cell culture

Hepa-1c1c7 cells (ATCC^®^) were grown as monolayers in alpha-modified Minimum Essential Medium Eagle (α-MEME) supplemented with 10% (v/v) heat- and charcoal-inactivated fetal bovine serum, 2 mM L-glutamine, 100 U/mL penicillin, and 100 µg/mL streptomycin sulfate. RAW264.7 (ATCC^®^) cells were grown in Dulbecco’s Modified Eagle’s Medium supplemented with 10% (v/v) fetal bovine serum, 2 mM L-glutamine, 100 U/mL penicillin, and 100 µg/mL streptomycin sulfate. Both cells were grown and cultured in a humidified 5% CO_2_ incubator. Routine culturing of Hepa-1c1c7 and RAW264.7 cells was achieved using either Trypsine/EDTA or sterile rubber scrappers^43,44^.

#### Treatment for chemopreventive NQO1 induction & NQO1 Western blotting

Overnight cultures of originally seeded Hepa-1c1c7 cells in 6-well plates were treated with either vehicle [0.1% dimethyl sulfoxide (DMSO) final concentration] in culture media or compounds at a final concentration of 10 µM. Cells were cultured for 48 h, then rinsed with Dulbecco’s phosphate-buffered saline (DPBS) and scraped in homogenization buffer. Cell suspensions were sonicated on ice (30% amplitude) for 5 s and centrifuged at 12,000×g for 5 min. Supernatants were utilized for both NQO1 activities. For NQO1 Western blotting, control, positive control-treated (4’-BF), or compound-treated cell lysate proteins (50 µg/lane) were loaded onto a 10% polyacrylamide gel and electrophoresed on a Biorad Tetra Cell (Biorad, USA) for 90 min at 110 volts. Following electrophoresis, proteins were blotted onto nitrocellulose membranes using a Biorad transfer module (60 min at 100 volts). Following electroblotting, blots were rapidly stained with Ponceau S stain to visualize the transferred proteins. Blots were cut alongside the Mwt. Ladder onto appropriate strip for each protein target (i.e. a strip for NQO1 and another for β-actin). Membrane strips were photographed, destained with Tris buffered saline with tween 20 (TBST) and then blocked for 1 h with 5% non-fat milk. Strips were then incubated with corresponding primary antibody (NQO1, Elabscience, USA or β-actin, Thermofisher Scientific, USA) at 4 °C overnight and gently rolled on a tube roller. Membrane streps were washed with TBST for 5 min before being incubated with corresponding horseradish peroxidase-conjugated secondary antibodies for 1 h on a tube roller. Following secondary antibody incubation, membrane strips were washed with TBST for 5 min. Membrane strips belonging to the same blot/replica were reassembled in the dark room of the imager for quick photographing. Protein bands were observed with an enzyme chemiluminescence kit (Pierce, USA). Bands were photographed and analyzed for densitometry of NQO1 bands relative to control and normalized to β-actin using a Biospectrum Imager (UVP, UK) and the Visionworks Acquisition and Analysis software package (version 8.20.17096.9551, Analytik Jena, USA)^44^. All the uncropped western blotting figures were inserted in the supplementary file.

#### Treatment of RAW264.7 for anti-inflammatory NO inhibition

96-well microwell plates were seeded with RAW264.7 cell stock (0.1 × 10^6^ cells/well) and treated by the standard technique^45^. Overnight-incubated RAW264.7 cell cultures were treated with DMSO at a final concentration of 0.1% v/v in fresh complete medium. Wells in the inflammation-inducing group were treated with 100 ng/mL LPS. Increasing amount (0.625, 1.25, 2.5, 5 and 10 µM, dissolved in DMSO) was utilized to treat cells in the presence of LPS. After 24 h of incubation, nitric oxide (NO) levels in each well were evaluated using the Griess test^46^. The absorbance at 520 nm was measured using a Tristar2 lbTM microplate reader (Berthold, Germany) after equal amounts of culture supernatants and Griess reagent were mixed and incubated at room temperature for 10 min to produce the colored diazonium salt. The percentage of NO inhibition in the test extract was compared to the group subjected to LPS-induced inflammation (% NO inhibition of LPS = 0). Concentration of the compound that caused inhibition of 50% of NO in LPS group (IC_50_) was derived from non-linear regression curve fit, using Graphpad Prism 6 (San Diego, USA).

#### Western blotting of iNOS protein expression

RAW264.7 cells were seeded at 1.5 × 10^6^ cells per well in 6-well plates. The cells were cultured overnight before being treated with the compounds (10 µM, final DMSO concentration 0.1% v/v) or indomethacin (250 µM) in the absence or presence of 100 ng/mL LPS. Western blotting was used to determine the relative protein expression of the pro-inflammatory marker iNOS, as previously described^43^. After 24 h of treatment, RAW264.7 cells were rinsed with ice-cold DPBS and scraped in homogenization buffer. On ice, cell suspensions were sonicated at 30% amplitude for 10 s. Sonicates were centrifuged at 12,000×g for 5 min. Supernatants were examined for iNOS protein expression, as previously described^45^. iNOS band detection and densitometric analysis was performed as described above. All the uncropped western blotting figures were inserted in the supplementary file.

### Statistical analysis

The data were analyzed using one-way analysis of variance (ANOVA) on GraphPad Prism 6 statistical software (San Diego, USA) to compare treated groups to vehicle control groups (significance was considered *p* < 0.05). The IC_50_ was estimated by fitting a non-linear regression curve to the concentration-response relationship.

### Computational studies

Are mentioned in the supplementary file. Table 4 mentioned the 2D-QSAR model attained and its descriptors.

**Table 4:** Descriptors of the QSAR model for % induction of NQO1 of the training set compounds.

Entry	ID	Coefficient	s	t	Descriptor
1	0	2027.15	507.543	3.994	Intercept
2	*D*_1_	0.540992	0.090	5.996	Min. total interaction for bond N-S
3	*D*_2_	3.80646	0.637	5.972	Max. valency for atom H
4	*D*_3_	-6.55445	1.631	-4.018	Max. atomic state energy for atom O
5	*D*_4_	-2.02368	0.296	-6.847	Min exchange energy for bond C-S
6	*D*_5_	-3.11199	0.419	-7.421	Relative number of H atoms

*N* = 25, *n* = 5, *R*^2^ = 0.836, *R*^2^cvOO = 0.831, *R*^2^cvMO = 0.999, *F* = 19.362, *s*^2^ = 0.001

Log(% induction of NQO1) = 2027.15 + (0.540992 x *D*_1_) + (3.80646 x *D*_2_) ‒ (6.55445 x *D*_3_) ‒ (2.02368 x *D*_4_) ‒ (3.11199 x *D*_5_)

## Electronic supplementary material

Below is the link to the electronic supplementary material.

Supplementary Material 1

## Data Availability

All data generated or analyzed during this study are included in this submitted article and its supplementary information file.
