# Supplementary material for: Curcumin mimics of potential chemoprevention with NQO1 induction properties
Source: Sci Rep. 2025 Jan 17;15:2332. doi: 10.1038/s41598-025-85588-w (PMC11748699; doi:10.1038/s41598-025-85588-w)
Supplement: Supplementary file 1 — Supplementary Material 1 [file 41598_2025_85588_MOESM1_ESM.docx]

**Curcumin mimics of potential chemoprevention with NQO1 induction properties**

Dalia R. Aboshouk^1^, Ahmed R. Hamed^2^, Siva S. Panda^3^, Mohamed S. Bekheit^1^, M. Adel Youssef^4^ & Adel S. Girgis*^,1^

^1^Department of Pesticide Chemistry, National Research Centre, Dokki, Giza 12622, Egypt

^2^Chemistry of Medicinal Plants Department, National Research Centre, Dokki, Giza 12622, Egypt

^3^Department of Chemistry and Biochemistry, Augusta University, Augusta, GA 30912, USA

^4^Department of Chemistry, Faculty of Science, Helwan University, Helwan, Egypt

*Corresponding author: [girgisas10@yahoo.com](mailto:girgisas10@yahoo.com), as.girgis@nrc.sci.eg

**Supplementary material**

**Table titles**

**Table S1.** Observed and estimated % induction of NQO1 of the training set compounds.

**Table S2.** Molecular descriptor values of the QSAR model for the training set compounds.

**Table S3**. Observed/estimated properties and descriptor values of the test set compounds.

**Table S4.** RMSD of protein of PDB: 4IQK.

**Table S5.** RMSD of the best conformation pose of compound **5ab** in the protein of PDB: 4IQK.

**Table S6.** RMSD of the best conformation pose of compound **5ac** in in the protein of PDB: 4IQK.

**Table S7.** RMSF of protein of PDB: 4IQK.

**Table S8.** RMSF of the best conformation pose of compound **5ab** in in the protein of PDB: 4IQK.

**Table S9.** RMSF of the best conformation pose of compound **5ac** in in the protein of PDB: 4IQK.

**Figure captions**

**Fig. S1.** IR spectrum of compound **5w** (KBr pellet).

**Fig. S2.** ^1^H-NMR spectrum of compound **5w** in DMSO-*d_6_*.

**Fig. S3.** ^13^C-NMR spectrum of compound **5w** in DMSO-*d_6_*.

**Fig. S4.** IR spectrum of compound **5x** (KBr pellet).

**Fig. S5.** ^1^H-NMR spectrum of compound **5x** in DMSO-*d_6_*.

**Fig. S6.** ^13^C-NMR spectrum of compound **5x** in DMSO-*d_6_*.

**Fig. S7.** IR spectrum of compound **5y** (KBr pellet).

**Fig. S8.** ^1^H-NMR spectrum of compound **5y** in DMSO-*d_6_*.

**Fig. S9.** ^13^C-NMR spectrum of compound **5y** in DMSO-*d_6_*.

**Fig. S10.** IR spectrum of compound **5z** (KBr pellet).

**Fig. S11.** ^1^H-NMR spectrum of compound **5z** in DMSO-*d_6_*.

**Fig. S12.** ^13^C-NMR spectrum of compound **5z** in DMSO-*d_6_*.

**Fig. S13.** IR spectrum of compound **5aa** (KBr pellet).

**Fig. S14.** ^1^H-NMR spectrum of compound **5aa** in DMSO-*d_6_*.

**Fig. S15.** ^13^C-NMR spectrum of compound **5aa** in DMSO-*d_6_*.

**Fig. S16.** IR spectrum of compound **5ab** (KBr pellet).

**Fig. S17.** ^1^H-NMR spectrum of compound **5ab** in DMSO-*d_6_*.

**Fig. S18.** ^13^C-NMR spectrum of compound **5ab** in DMSO-*d_6_*.

**Fig. S19.** IR spectrum of compound **5ac** (KBr pellet).

**Fig. S20.** ^1^H-NMR spectrum of compound **5ac** in DMSO-*d_6_*.

**Fig. S21.** ^13^C-NMR spectrum of compound **5ac** in DMSO-*d_6_*.

**Fig. S22.** IR spectrum of compound **5ad** (KBr pellet).

**Fig. S23.** ^1^H-NMR spectrum of compound **5ad** in DMSO-*d_6_*.

**Fig. S24.** ^13^C-NMR spectrum of compound **5ad** in DMSO-*d_6_*.

**Fig. S25.** IR spectrum of compound **5ae** (KBr pellet).

**Fig. S26.** ^1^H-NMR spectrum of compound **5ae** in DMSO-*d_6_*.

**Fig. S27.** ^13^C-NMR spectrum of compound **5ae** in DMSO-*d_6_*.

**Fig. S28.** IR spectrum of compound **5af** (KBr pellet).

**Fig. S29.** ^1^H-NMR spectrum of compound **5af** in DMSO-*d_6_*.

**Fig. S30.** ^13^C-NMR spectrum of compound **5af** in DMSO-*d_6_*.

**Fig. S31.** IR spectrum of compound **5ag** (KBr pellet).

**Fig. S32.** ^1^H-NMR spectrum of compound **5ag** in DMSO-*d_6_*.

**Fig. S33.** ^13^C-NMR spectrum of compound **5ag** in DMSO-*d_6_*.

**Fig. S34.** IR spectrum of compound **5ah** (KBr pellet).

**Fig. S35.** ^1^H-NMR spectrum of compound **5ah** in DMSO-*d_6_*.

**Fig. S36.** ^13^C-NMR spectrum of compound **5ah** in DMSO-*d_6_*.

**Fig. S37.** Western blotting of NQO1 induction by pre-synthesized piperidones **5a‒5v**. Hepa1c1c7 cells were treated for 48h with vehicle (0.1% DMSO) or 10 µM of compounds **5a‒5v**. 4'BF was used as reference NQO1 inducer. Cell lysates were prepared and NQO1 expression was detected as mentioned in the experimental section.

**Fig. S38.** QSAR plot representing the observed versus predicted property “log(% induction of NQO1)” for the training set compounds at 10 μM.

**Fig. S39.** Uncropped western blotting of NQO1 induction by pre-synthesized piperidones **5a‒5v**. Hepa1c1c7 cells were treated for 48h with vehicle (0.1% DMSO) or 10 µM of compounds **5a‒5v**. 4'BF was used as reference NQO1 inducer. Cell lysates were prepared and NQO1 expression was detected as mentioned in the experimental section. Image sensitivity was enhanced in B, compared to auto enhancement A. This was done to reveal the membrane strips in the background as no photos were taken for the membranes before chemiluminescence in this occasion for this experiment.

**Fig. S40.** Supplementary images of Figure 3. (A) Colored mobile camera photo of the Ponceau stained membranes of each replica showing the cut strips before blocking step. (B) Imager photos of membrane strips before chemiluminescence. Chemiluminescence image of the replica (2 replicas) of Figure 3. Blue arrow refers to the crude image source of Figure 3. Red shapes indicate the samples involved in the present study. (D) Uncropped western blotting of NQO1 induction by the synthesized piperidones **5w**, **5x**, **5ab**, **5ac** and **5ae** exhibited in the manuscript. Hepa1c1c7 cells were treated for 48h with vehicle (0.1% DMSO) or 10 µM of the tested compounds. 4'-BF was used as reference NQO1 inducer. Cell lysates were prepared and NQO1 expression was detected as mentioned in the experimental section.

**Fig. S41.** Supplementary images of Figure 4. (A) Colored mobile camera photo of the Ponceau S- stained membranes of REPLICA 1 & REPLICA 2 showing the cut strips before blocking step. (B) Imager photos of membrane strips OF REPLICA 1 & REPLICA 2 before chemiluminescence. (C) Chemiluminescence image of the REPLICA 1 & REPLICA 2 of Figure 4, Blue arrow refers to the crude image source of Figure 4. (D) Colored mobile camera photo of the Ponceau stained membranes of REPLICA 3 & REPLICA 4 showing the cut strips before blocking step. (E) Imager photos of membrane strips OF REPLICA 3 & REPLICA 4 before chemiluminescence. (F) Chemiluminescence image of the REPLICA 3 & REPLICA 4 of Figure 4. (D) Uncropped western blotting showing NQO1 induction by 2.5 and 5 µM of compounds **5ab** and **5ac** mentioned in the manuscript as Figure 4.

**Fig. S42.** Supplementary images of Figure 7. (A) Colored mobile camera photo of the Ponceau stained membranes of REPLICA 1, REPLICA 2 & REPLICA 3 showing the cut strips before blocking step. (B) Imager photos of membrane strips REPLICA 1, REPLICA 2 & REPLICA 3 before chemiluminescence. (C) Chemiluminescence image of REPLICA 1, REPLICA 2 & REPLICA 3 of Figure 7, Blue arrow refers to the crude image source of Figure 7. All replicas contain samples that are essential for the analysis. (D) Uncropped western blot showing concentration-dependent inhibition of LPS-induced iNOS expression by **5ab** and **5ac** in RAW264.7 macrophages mentioned in Figure 7 of manuscript.

**In-silico studies**

In-silico studies including QSAR (quantitative structure-activity relation), molecular docking and dynamic simulation are considered in this study. QSAR is a computational technique capable of developing mathematical equations expressing the estimated efficacies/potencies in terms of descriptors (physico-chemical) parameters. Detection of the key descriptor(s) essential for bio-properties can identify the rules/parameters necessary for the efficacy. Also, can be used for optimizing/discovering newer hits/leads^1,2^. Molecular docking is a useful technique for determining the key functional group(s) giving interaction with the lead amino acid(s) of the protein active site controlling the biochemical interaction^3^. Molecular dynamic simulation studies are accessible tools for supporting the stability of an effective agent discovered during molecular docking studies in the protein active site^4^.

**2D-QSAR study**

The synthesized agents **5a‒n**, **5p‒r**, **5t-x**, **5ab**, **5ac** and **5ae** were utilized as the training set for developing the 2D-QSAR modeling by CODESSA-Pro (comprehensive descriptors for structural and statistical analysis) software while compounds **5o** and **5s** (representing promising and potent agents) were considered as the test set^1,2^. Geometry of the compounds was initially optimized by AM1 technique using hyperChem 8.0 then, uploaded to CODESSA-Pro for final geometrical structure optimization by MOPAC. CODESSA-Pro calculated 797 molecular descriptors (constitutional, topological, geometrical, charge-related, semi-empirical, molecular-type, atomic-type and bond-type descriptors) for the exported agents. Mathematical transformation of the experimental property “% induction of NQO1 values” [including property, 1/property, log(property) and 1/ log(property)] were used searching for the best QSAR model. CODESSA-Pro can utilize/run the best multi-linear regression (BMLR) technique/process which is a stepwise search for the best *n*-parameter regression equations (where *n* stands for the number of descriptors used), based on the highest *R*^2^ (squared correlation coefficient), *R*^2^cvOO (squared cross-validation “leave one-out, LOO” coefficient), *R*^2^cvMO (squared cross-validation “leave many-out up to 20% of the training set, LMO” coefficient), *F* (Fisher statistical significance criteria) values, and *s*^2^ (standard deviation). The QSAR up to 5-descriptor model describing the biological activity of the tested agents were generated (obeying the thumb rule of 5:1 which is the ratio between the data points and the number of QSAR descriptor) (Tables S1‒S3, Fig. S38).

Equations needed for calculating the descriptors are.

$$E_{tot} \left( AB \right)= E_{C \left( AB \right)} + E_{exc}\left( AB \right)\ldots\ldots\ldots(S1)$$

Where, *A* and *B* are two different atomic species. The $E_{C}(AB)$ and $E_{exc} (AB)$ are electrostatic interaction and electronic exchange energies between the two atomic species.

$$V_{fA}= V_{max} - P_{A}\ldots\ldots\ldots(S2)$$

Where, $V_{max}$ is the maximum valence of atom *A*. $P_{A}$ is the total electronic population on atom *A*.

$$E_{exc} \left( AB \right)= \sum_{\mu,\nu\in A} \sum_{\lambda,\sigma\in B} P_{\mu\lambda}P_{\nu\sigma}\left\langle\mu\lambda| \nu\sigma\right\rangle\ldots\ldots\ldots(S3)$$

Where, *A* and *B* are two atomic species. $P_{\mu\nu}$, $P_{\lambda\sigma}$ are the dentistry matrix elements over atomic basis $\left\{ \mu\nu\lambda\sigma\right\}$. The $\left\langle\mu\nu| \lambda\sigma\right\rangle$ is the electron repulsion integrals on atomic basis $\left\{ \mu\nu\lambda\sigma\right\}$.

**Molecular docking studies**

Molecular docking study using CDOCKER protocol in Discovery Studio 4.1 Software was carried out. The synthesized compounds were docked into the PDB: 4IQK active site. The X-ray crystallographic structure of PDB: 4IQK was downloaded^5^ and optimized by the standard protocol (force field: CHARMm, Partial charge: MMFF94, resolution: 1.97 Å, radius of the active site employed: 8.0309 Å, RMS gradient: 0.0897).

The binding mode of the compounds (**5ab** and **5ac**) in the protein active site was studied to explain their biological results and to detect the essential bonding/non-bonding interactions with the active site/pocket protein amino acid(s).

**Molecular dynamic simulation studies**

Molecular dynamic simulation studies using Discovery Studio 4.1 Software were carried out for the protein of PDB: 4IQK and best poses detected in molecular docking studies for compounds **5ab** and **5ac** utilizing the standard protocol (standard dynamic cascade). For the tested agents, the pose revealing best docking interaction was dragged and dropped in the protein of PDB: 4IQK, optimized by the standard protocol (force field: CHARMm, Partial charge: MMFF94) then the standard molecular dynamic protocol was applied.

Minimization 1

Algorithm: Steepest Descent, Maximum Steps: 2000, RMS Gradient: 1.0.

Minimization 2

Algorithm: Conjugate Gradient, Max. Steps: 2000, RMS Gradient: 0.1

Heating

Simulation Time (ps): 4, Time Steps (fs): 2, Initial Temperature: 50, Target Temperature: 300, Adjust Velocity Frequency: 1000, Save Results Interval (ps): 2.

Equilibration

Simulation Time (ps): 20, Time Step (fs): 2, Target Temperature: 300, Adjust Velocity Frequency: 1000, Save Results Intervals (ps): 2.

Production

Simulation Time (ps): 200, Time Steps (fs): 2, Target Temperature: 300, Type: NVT.

Implicit Solvent Model

Generalized Born with a simple Switching (GBSW).

RMSD and RMSF were obtained upon applying trajectory analysis on the output files obtained from the molecular dynamic simulation studies.

**References**

1. Ghanim, A. M., Girgis, A. S., Kariuki, B. M., Samir, N., Said, M. F., Abdelnaser, A., Nasr, S., Bekheit, M. S., Abdelhameed, M. F., Almalki, A. J., Ibrahim, T. S., Panda, S. S. Design and synthesis of ibuprofen-quinoline conjugates as potential anti-inflammatory and analgesic drug candidates. *Bioorg. Chem.* **119**, 105557. <https://doi.org/10.1016/j.bioorg.2021.105557> (2022).
2. Tiwari, A. D., Panda, S. S., Girgis, A. S., Sahu, S., George, R. F., Srour, A. M., La Starza, B., Asiri, A. M., Hall, C. D., Katritzky, A. R. Microwave assisted synthesis and QSAR study of novel NSAID acetaminophen conjugates with amino acid linkers. *Org. Biomol. Chem.* **12**, 7238–7249, doi:10.1039/c4ob01281j (2014).
3. Wyman, K. A., Girgis, A. S., Surapaneni, P. S., Moore, J. M., Abo Shama, N. M., Mahmoud, S. H., Mostafa, A., Barghash, R. F., Juan, Z., Dobaria, R. D., Almalki, A. J., Ibrahim, T. S., Panda, S. S. Synthesis of potential antiviral agents for SARS-CoV-2 using molecular hybridization approach. *Molecules* **27**, 5923. <https://doi.org/10.3390/molecules27185923> (2022).
4. Azmy, E. M., Hagras, M., Ewida, M. A., Doghish, A. S., Khidr, E. G., El-Husseiny, A. A., Gomaa, M. H., Refaat, H. M., Ismail, N. S. M., Nassar, I. F., Lashin, W. H. Development of pyrolo[2,3-*c*]pyrazole, pyrolo[2,3-*d*]pyrimidine and their bioisosteres as novel CDK2 inhibitors with potent *in vitro* apoptotic anti-proliferative activity: Synthesis, biological evaluation and molecular dynamics investigations. *Bioorg. Chem.* **139**, 106729, <https://doi.org/10.1016/j.bioorg.2023.106729> (2023).
5. <https://www.rcsb.org/structure/4IQK>

**Table S1.** Observed and estimated % induction of NQO1 of the training set compounds.

| Entry | Compd. | log (observed, % induction of NQO1) | Observed (% induction of NQO1) | log (estimated, % induction of NQO1) | Estimated (% induction of NQO1) | Error^*^ |
| --- | --- | --- | --- | --- | --- | --- |
| 1 | **5a** | 1.51287 | 32.574 | 1.54409 | 35.002 | -2.428 |
| 2 | **5b** | 1.54084 | 34.741 | 1.52468 | 33.472 | 1.269 |
| 3 | **5c** | 1.48206 | 30.343 | 1.44058 | 27.579 | 2.764 |
| 4 | **5d** | 1.65575 | 45.264 | 1.60702 | 40.459 | 4.805 |
| 5 | **5e** | 1.62547 | 42.215 | 1.61461 | 41.173 | 1.042 |
| 6 | **5f** | 1.52713 | 33.661 | 1.47699 | 29.991 | 3.670 |
| 7 | **5g** | 1.56359 | 36.609 | 1.5863 | 38.574 | -1.965 |
| 8 | **5h** | 1.57492 | 37.577 | 1.56825 | 37.004 | 0.573 |
| 9 | **5i** | 1.43334 | 27.123 | 1.42143 | 26.389 | 0.734 |
| 10 | **5j** | 1.50273 | 31.822 | 1.53561 | 34.325 | -2.503 |
| 11 | **5k** | 1.56679 | 36.88 | 1.59653 | 39.494 | -2.614 |
| 12 | **5l** | 1.50177 | 31.752 | 1.56935 | 37.098 | -5.346 |
| 13 | **5m** | 1.46925 | 29.461 | 1.47619 | 29.936 | -0.475 |
| 14 | **5n** | 1.47648 | 29.956 | 1.47395 | 29.782 | 0.174 |
| 15 | **5p** | 1.52285 | 33.331 | 1.50988 | 32.350 | 0.981 |
| 16 | **5q** | 1.51911 | 33.045 | 1.54144 | 34.789 | -1.744 |
| 17 | **5r** | 1.58157 | 38.157 | 1.52458 | 33.464 | 4.693 |
| 18 | **5t** | 1.5028 | 31.827 | 1.47068 | 29.558 | 2.269 |
| 19 | **5u** | 1.4648 | 29.161 | 1.45018 | 28.196 | 0.965 |
| 20 | **5v** | 1.53648 | 34.394 | 1.56445 | 36.682 | -2.288 |
| 21 | **5w** | 1.52163 | 33.238 | 1.517 | 32.885 | 0.353 |
| 22 | **5x** | 1.57165 | 37.295 | 1.5705 | 37.196 | 0.099 |
| 23 | **5ab** | 1.71233 | 51.562 | 1.68667 | 48.604 | 2.958 |
| 24 | **5ac** | 1.6608 | 45.793 | 1.68052 | 47.920 | -2.127 |
| 25 | **5ae** | 1.28298 | 19.186 | 1.3585 | 22.830 | -3.644 |

^*^Error is the difference between the observed and estimated property.

**Table S2.** Molecular descriptor values of the QSAR model for the training set compounds.

| Entry | Compd | Descriptors*^*^* | | | | |
| --- | --- | --- | --- | --- | --- | --- |
|  |  | *D*_1_ | *D*_2_ | *D*_3_ | *D*_4_ | *D*_5_ |
| 1 | **5a** | 27.1888 | 0.83812 | 310.554 | 3.2836 | 0.43182 |
| 2 | **5b** | 27.0697 | 0.83778 | 310.5559 | 3.2315 | 0.44681 |
| 3 | **5c** | 27.1164 | 0.85452 | 310.572 | 3.2446 | 0.46 |
| 4 | **5d** | 27.1085 | 0.83515 | 310.5542 | 3.2947 | 0.38636 |
| 5 | **5e** | 26.9263 | 0.85529 | 310.5672 | 3.2105 | 0.40426 |
| 6 | **5f** | 26.9285 | 0.85597 | 310.5662 | 3.2594 | 0.42 |
| 7 | **5g** | 27.1698 | 0.8099 | 310.556 | 3.268 | 0.38636 |
| 8 | **5h** | 26.9673 | 0.84656 | 310.5712 | 3.215 | 0.40426 |
| 9 | **5i** | 26.9692 | 0.84587 | 310.5718 | 3.2606 | 0.42 |
| 10 | **5j** | 27.0547 | 0.79949 | 310.5585 | 3.2346 | 0.38636 |
| 11 | **5k** | 27.0005 | 0.84292 | 310.5672 | 3.216 | 0.40426 |
| 12 | **5l** | 26.9568 | 0.855 | 310.5545 | 3.2574 | 0.42 |
| 13 | **5m** | 27.2087 | 0.83933 | 310.5553 | 3.2772 | 0.46 |
| 14 | **5n** | 27.0916 | 0.84008 | 310.5562 | 3.2275 | 0.4717 |
| 15 | **5p** | 27.2283 | 0.85804 | 310.5574 | 3.3214 | 0.44231 |
| 16 | **5q** | 27.1318 | 0.85956 | 310.5647 | 3.2404 | 0.45455 |
| 17 | **5r** | 26.9771 | 0.85996 | 310.5611 | 3.2029 | 0.46552 |
| 18 | **5t** | 26.9993 | 0.81499 | 310.5608 | 3.2607 | 0.39474 |
| 19 | **5u** | 26.8548 | 0.82745 | 310.5581 | 3.2338 | 0.41463 |
| 20 | **5v** | 27.0337 | 0.85482 | 310.5699 | 3.212 | 0.43182 |
| 21 | **5w** | 27.1014 | 0.80607 | 310.5562 | 3.2761 | 0.38636 |
| 22 | **5x** | 27.028 | 0.84942 | 310.5586 | 3.2763 | 0.40426 |
| 23 | **5ab** | 27.2631 | 0.86138 | 310.5554 | 3.2561 | 0.44231 |
| 24 | **5ac** | 27.207 | 0.85694 | 310.5607 | 3.1998 | 0.45455 |
| 25 | **5ae** | 26.9365 | 0.86636 | 310.57 | 3.2742 | 0.45455 |

^*^*D*_1_ = Min. total interaction for bond N-S, *D*_2_ = Max. valency for atom H, *D*_3_ = Max. atomic state energy for atom O, *D*_4_ = Min exchange energy for bond C-S, *D*_5_ = Relative number of H atoms.

**Table S3**. Observed/estimated properties and descriptor values of the test set compounds.

| Entry | Compd. | Observed (% induction of NQO1) | Estimated  (% induction of NQO1) | Error^a^ | Descriptors^b^ | | | | |
| --- | --- | --- | --- | --- | --- | --- | --- | --- | --- |
|  |  |  |  |  | *D*_1_ | *D*_2_ | *D*_3_ | *D*_4_ | *D*_5_ |
| 1 | **5o** | 29.956 | 22.600 | 7.356 | 26.9748 | 0.85398 | 310.5737 | 3.2089 | 0.48214 |
| 2 | **5s** | 45.694 | 40.808 | 4.886 | 27.0682 | 0.87791 | 310.5684 | 3.2028 | 0.46032 |

^a^ Error is the difference between the observed and estimated property (IC_50_, *μ*M).

^b^ *D*_1_ = Min. total interaction for bond N-S, *D*_2_ = Max. valency for atom H, *D*_3_ = Max. atomic state energy for atom O, *D*_4_ = Min exchange energy for bond C-S, *D*_5_ = Relative number of H atoms.

**Table S4.** RMSD of protein of PDB: 4IQK.

| Entry | Item | RMSD (Å) |
| --- | --- | --- |
| 1 | Conformation 1 | 0 |
| 2 | Conformation 2 | 1.25746 |
| 3 | Conformation 3 | 1.24612 |
| 4 | Conformation 4 | 1.50706 |
| 5 | Conformation 5 | 1.65681 |
| 6 | Conformation 6 | 1.70054 |
| 7 | Conformation 7 | 1.79461 |
| 8 | Conformation 8 | 1.89205 |
| 9 | Conformation 9 | 1.92199 |
| 10 | Conformation 10 | 2.03729 |
| 11 | Conformation 11 | 2.03081 |
| 12 | Conformation 12 | 1.94261 |
| 13 | Conformation 13 | 2.06902 |
| 14 | Conformation 14 | 1.98302 |
| 15 | Conformation 15 | 1.7962 |
| 16 | Conformation 16 | 1.89861 |
| 17 | Conformation 17 | 1.80476 |
| 18 | Conformation 18 | 1.84312 |
| 19 | Conformation 19 | 1.94261 |
| 20 | Conformation 20 | 1.99751 |
| 21 | Conformation 21 | 2.0323 |
| 22 | Conformation 22 | 1.96482 |
| 23 | Conformation 23 | 1.93911 |
| 24 | Conformation 24 | 2.02789 |
| 25 | Conformation 25 | 2.0674 |
| 26 | Conformation 26 | 1.9655 |
| 27 | Conformation 27 | 1.93002 |
| 28 | Conformation 28 | 1.96292 |
| 29 | Conformation 29 | 2.01903 |
| 30 | Conformation 30 | 1.98107 |
| 31 | Conformation 31 | 1.83378 |
| 32 | Conformation 32 | 1.76196 |
| 33 | Conformation 33 | 1.98953 |
| 34 | Conformation 34 | 1.97102 |
| 35 | Conformation 35 | 2.10116 |
| 36 | Conformation 36 | 2.05152 |
| 37 | Conformation 37 | 1.96734 |
| 38 | Conformation 38 | 2.0751 |
| 39 | Conformation 39 | 2.10809 |
| 40 | Conformation 40 | 2.17626 |
| 41 | Conformation 41 | 2.22128 |
| 42 | Conformation 42 | 2.12112 |
| 43 | Conformation 43 | 2.13901 |
| 44 | Conformation 44 | 2.08182 |
| 45 | Conformation 45 | 1.84761 |
| 46 | Conformation 46 | 2.0039 |
| 47 | Conformation 47 | 1.88108 |
| 48 | Conformation 48 | 1.83768 |
| 49 | Conformation 49 | 1.93687 |
| 50 | Conformation 50 | 2.04371 |
| 51 | Conformation 51 | 2.04786 |
| 52 | Conformation 52 | 2.11161 |
| 53 | Conformation 53 | 2.13277 |
| 54 | Conformation 54 | 2.04524 |
| 55 | Conformation 55 | 2.08824 |
| 56 | Conformation 56 | 1.97109 |
| 57 | Conformation 57 | 2.04276 |
| 58 | Conformation 58 | 2.0775 |
| 59 | Conformation 59 | 2.16854 |
| 60 | Conformation 60 | 2.10121 |
| 61 | Conformation 61 | 2.11596 |
| 62 | Conformation 62 | 2.20727 |
| 63 | Conformation 63 | 2.15059 |
| 64 | Conformation 64 | 2.15277 |
| 65 | Conformation 65 | 2.20199 |
| 66 | Conformation 66 | 2.14544 |
| 67 | Conformation 67 | 2.08754 |
| 68 | Conformation 68 | 2.12223 |
| 69 | Conformation 69 | 2.22633 |
| 70 | Conformation 70 | 2.17637 |
| 71 | Conformation 71 | 2.25165 |
| 72 | Conformation 72 | 2.19968 |
| 73 | Conformation 73 | 2.19009 |
| 74 | Conformation 74 | 2.15261 |
| 75 | Conformation 75 | 2.30296 |
| 76 | Conformation 76 | 2.2159 |
| 77 | Conformation 77 | 2.14319 |
| 78 | Conformation 78 | 2.28307 |
| 79 | Conformation 79 | 2.25473 |
| 80 | Conformation 80 | 2.18582 |
| 81 | Conformation 81 | 2.24299 |
| 82 | Conformation 82 | 2.22799 |
| 83 | Conformation 83 | 2.32434 |
| 84 | Conformation 84 | 2.20194 |
| 85 | Conformation 85 | 2.1896 |
| 86 | Conformation 86 | 2.34726 |
| 87 | Conformation 87 | 2.23836 |
| 88 | Conformation 88 | 2.30066 |
| 89 | Conformation 89 | 2.32611 |
| 90 | Conformation 90 | 2.39071 |
| 91 | Conformation 91 | 2.25511 |
| 92 | Conformation 92 | 2.39302 |
| 93 | Conformation 93 | 2.30596 |
| 94 | Conformation 94 | 2.31907 |
| 95 | Conformation 95 | 2.30068 |
| 96 | Conformation 96 | 2.29744 |
| 97 | Conformation 97 | 2.34813 |
| 98 | Conformation 98 | 2.4187 |
| 99 | Conformation 99 | 2.46953 |
| 100 | Conformation 100 | 2.30353 |
| 101 | Average RMSD | 2.05116 |

**Table S5.** RMSD of the best conformation pose of compound **5ab** in the protein of PDB: 4IQK.

| Entry | Item | RMSD (Å) |
| --- | --- | --- |
| 1 | Conformation 1 | 0 |
| 2 | Conformation 2 | 1.29261 |
| 3 | Conformation 3 | 1.58368 |
| 4 | Conformation 4 | 1.60421 |
| 5 | Conformation 5 | 1.65945 |
| 6 | Conformation 6 | 1.42237 |
| 7 | Conformation 7 | 1.47604 |
| 8 | Conformation 8 | 1.66592 |
| 9 | Conformation 9 | 1.67545 |
| 10 | Conformation 10 | 1.7219 |
| 11 | Conformation 11 | 1.65962 |
| 12 | Conformation 12 | 1.64053 |
| 13 | Conformation 13 | 1.63118 |
| 14 | Conformation 14 | 1.83855 |
| 15 | Conformation 15 | 1.88078 |
| 16 | Conformation 16 | 1.82841 |
| 17 | Conformation 17 | 1.61865 |
| 18 | Conformation 18 | 1.68645 |
| 19 | Conformation 19 | 1.688 |
| 20 | Conformation 20 | 1.76094 |
| 21 | Conformation 21 | 1.85224 |
| 22 | Conformation 22 | 1.7983 |
| 23 | Conformation 23 | 2.01968 |
| 24 | Conformation 24 | 2.08192 |
| 25 | Conformation 25 | 1.87586 |
| 26 | Conformation 26 | 1.89234 |
| 27 | Conformation 27 | 1.82684 |
| 28 | Conformation 28 | 1.82569 |
| 29 | Conformation 29 | 1.90114 |
| 30 | Conformation 30 | 1.96562 |
| 31 | Conformation 31 | 1.86287 |
| 32 | Conformation 32 | 1.81352 |
| 33 | Conformation 33 | 1.77277 |
| 34 | Conformation 34 | 1.76336 |
| 35 | Conformation 35 | 1.86619 |
| 36 | Conformation 36 | 1.71607 |
| 37 | Conformation 37 | 1.77117 |
| 38 | Conformation 38 | 1.92775 |
| 39 | Conformation 39 | 2.00919 |
| 40 | Conformation 40 | 2.03389 |
| 41 | Conformation 41 | 1.96256 |
| 42 | Conformation 42 | 1.88707 |
| 43 | Conformation 43 | 1.90825 |
| 44 | Conformation 44 | 2.02125 |
| 45 | Conformation 45 | 1.97296 |
| 46 | Conformation 46 | 2.02111 |
| 47 | Conformation 47 | 2.14569 |
| 48 | Conformation 48 | 2.21011 |
| 49 | Conformation 49 | 2.21266 |
| 50 | Conformation 50 | 2.34873 |
| 51 | Conformation 51 | 2.23651 |
| 52 | Conformation 52 | 2.22427 |
| 53 | Conformation 53 | 2.16245 |
| 54 | Conformation 54 | 2.05522 |
| 55 | Conformation 55 | 2.12589 |
| 56 | Conformation 56 | 2.13446 |
| 57 | Conformation 57 | 2.24196 |
| 58 | Conformation 58 | 2.22303 |
| 59 | Conformation 59 | 2.16612 |
| 60 | Conformation 60 | 2.18035 |
| 61 | Conformation 61 | 2.15567 |
| 62 | Conformation 62 | 2.13524 |
| 63 | Conformation 63 | 2.19408 |
| 64 | Conformation 64 | 2.2822 |
| 65 | Conformation 65 | 2.27758 |
| 66 | Conformation 66 | 2.2075 |
| 67 | Conformation 67 | 2.25662 |
| 68 | Conformation 68 | 2.16294 |
| 69 | Conformation 69 | 2.16375 |
| 70 | Conformation 70 | 2.18357 |
| 71 | Conformation 71 | 2.2964 |
| 72 | Conformation 72 | 2.34038 |
| 73 | Conformation 73 | 2.2884 |
| 74 | Conformation 74 | 2.21835 |
| 75 | Conformation 75 | 2.2761 |
| 76 | Conformation 76 | 2.38052 |
| 77 | Conformation 77 | 2.40351 |
| 78 | Conformation 78 | 2.40482 |
| 79 | Conformation 79 | 2.38917 |
| 80 | Conformation 80 | 2.29919 |
| 81 | Conformation 81 | 2.30834 |
| 82 | Conformation 82 | 2.3375 |
| 83 | Conformation 83 | 2.34827 |
| 84 | Conformation 84 | 2.39214 |
| 85 | Conformation 85 | 2.22298 |
| 86 | Conformation 86 | 2.27665 |
| 87 | Conformation 87 | 2.27897 |
| 88 | Conformation 88 | 2.39082 |
| 89 | Conformation 89 | 2.426 |
| 90 | Conformation 90 | 2.45473 |
| 91 | Conformation 91 | 2.3498 |
| 92 | Conformation 92 | 2.35948 |
| 93 | Conformation 93 | 2.37309 |
| 94 | Conformation 94 | 2.38734 |
| 95 | Conformation 95 | 2.46771 |
| 96 | Conformation 96 | 2.47872 |
| 97 | Conformation 97 | 2.49879 |
| 98 | Conformation 98 | 2.45321 |
| 99 | Conformation 99 | 2.51151 |
| 100 | Conformation 100 | 2.45717 |
| 101 | Average RMSD | 2.04441 |

**Table S6.** RMSD of the best conformation pose of compound **5ac** in in the protein of PDB: 4IQK.

| Entry | Item | RMSD (Å) |
| --- | --- | --- |
| 1 | Conformation 1 | 0 |
| 2 | Conformation 2 | 1.48899 |
| 3 | Conformation 3 | 1.63477 |
| 4 | Conformation 4 | 1.54228 |
| 5 | Conformation 5 | 1.68495 |
| 6 | Conformation 6 | 1.72931 |
| 7 | Conformation 7 | 1.61157 |
| 8 | Conformation 8 | 1.53423 |
| 9 | Conformation 9 | 1.57371 |
| 10 | Conformation 10 | 1.80008 |
| 11 | Conformation 11 | 1.70406 |
| 12 | Conformation 12 | 1.83992 |
| 13 | Conformation 13 | 1.8859 |
| 14 | Conformation 14 | 1.77 |
| 15 | Conformation 15 | 1.66779 |
| 16 | Conformation 16 | 1.67878 |
| 17 | Conformation 17 | 1.76254 |
| 18 | Conformation 18 | 2.03253 |
| 19 | Conformation 19 | 2.02738 |
| 20 | Conformation 20 | 2.03744 |
| 21 | Conformation 21 | 2.0697 |
| 22 | Conformation 22 | 2.01553 |
| 23 | Conformation 23 | 2.0424 |
| 24 | Conformation 24 | 2.07503 |
| 25 | Conformation 25 | 2.07547 |
| 26 | Conformation 26 | 2.13419 |
| 27 | Conformation 27 | 2.1082 |
| 28 | Conformation 28 | 1.93758 |
| 29 | Conformation 29 | 1.9029 |
| 30 | Conformation 30 | 1.98255 |
| 31 | Conformation 31 | 1.92821 |
| 32 | Conformation 32 | 1.9687 |
| 33 | Conformation 33 | 2.0016 |
| 34 | Conformation 34 | 2.08334 |
| 35 | Conformation 35 | 2.09491 |
| 36 | Conformation 36 | 2.11672 |
| 37 | Conformation 37 | 2.14087 |
| 38 | Conformation 38 | 2.17843 |
| 39 | Conformation 39 | 2.24642 |
| 40 | Conformation 40 | 2.22133 |
| 41 | Conformation 41 | 2.16974 |
| 42 | Conformation 42 | 2.13659 |
| 43 | Conformation 43 | 2.30465 |
| 44 | Conformation 44 | 2.27432 |
| 45 | Conformation 45 | 2.40578 |
| 46 | Conformation 46 | 2.34481 |
| 47 | Conformation 47 | 2.39707 |
| 48 | Conformation 48 | 2.2288 |
| 49 | Conformation 49 | 2.22817 |
| 50 | Conformation 50 | 2.28055 |
| 51 | Conformation 51 | 2.26412 |
| 52 | Conformation 52 | 2.26744 |
| 53 | Conformation 53 | 2.22656 |
| 54 | Conformation 54 | 2.30736 |
| 55 | Conformation 55 | 2.39704 |
| 56 | Conformation 56 | 2.37665 |
| 57 | Conformation 57 | 2.5129 |
| 58 | Conformation 58 | 2.68342 |
| 59 | Conformation 59 | 2.50651 |
| 60 | Conformation 60 | 2.49999 |
| 61 | Conformation 61 | 2.43235 |
| 62 | Conformation 62 | 2.53926 |
| 63 | Conformation 63 | 2.50695 |
| 64 | Conformation 64 | 2.46553 |
| 65 | Conformation 65 | 2.42593 |
| 66 | Conformation 66 | 2.52038 |
| 67 | Conformation 67 | 2.40952 |
| 68 | Conformation 68 | 2.45724 |
| 69 | Conformation 69 | 2.4565 |
| 70 | Conformation 70 | 2.52227 |
| 71 | Conformation 71 | 2.43283 |
| 72 | Conformation 72 | 2.44409 |
| 73 | Conformation 73 | 2.426 |
| 74 | Conformation 74 | 2.41777 |
| 75 | Conformation 75 | 2.45365 |
| 76 | Conformation 76 | 2.39529 |
| 77 | Conformation 77 | 2.40317 |
| 78 | Conformation 78 | 2.49961 |
| 79 | Conformation 79 | 2.5789 |
| 80 | Conformation 80 | 2.51114 |
| 81 | Conformation 81 | 2.58239 |
| 82 | Conformation 82 | 2.58705 |
| 83 | Conformation 83 | 2.6442 |
| 84 | Conformation 84 | 2.55977 |
| 85 | Conformation 85 | 2.51644 |
| 86 | Conformation 86 | 2.5465 |
| 87 | Conformation 87 | 2.66682 |
| 88 | Conformation 88 | 2.75754 |
| 89 | Conformation 89 | 2.63887 |
| 90 | Conformation 90 | 2.54987 |
| 91 | Conformation 91 | 2.60407 |
| 92 | Conformation 92 | 2.61174 |
| 93 | Conformation 93 | 2.65963 |
| 94 | Conformation 94 | 2.61098 |
| 95 | Conformation 95 | 2.61611 |
| 96 | Conformation 96 | 2.614 |
| 97 | Conformation 97 | 2.4888 |
| 98 | Conformation 98 | 2.44036 |
| 99 | Conformation 99 | 2.45558 |
| 100 | Conformation 100 | 2.5737 |
| 101 | Average RMSD | 2.22166 |

**Table S7.** RMSF of protein of PDB: 4IQK.

| Entry | Item | RMSF |
| --- | --- | --- |
| 1 | GLY325 | 2.7663 |
| 2 | ARG326 | 2.60444 |
| 3 | LEU327 | 1.159 |
| 4 | ILE328 | 0.865669 |
| 5 | TYR329 | 1.08862 |
| 6 | THR330 | 0.837901 |
| 7 | ALA331 | 0.613016 |
| 8 | GLY332 | 0.699885 |
| 9 | GLY333 | 0.623828 |
| 10 | TYR334 | 1.37288 |
| 11 | PHE335 | 0.862475 |
| 12 | ARG336 | 1.62019 |
| 13 | GLN337 | 1.20083 |
| 14 | SER338 | 0.638159 |
| 15 | LEU339 | 0.752434 |
| 16 | SER340 | 0.848743 |
| 17 | TYR341 | 1.4085 |
| 18 | LEU342 | 0.66621 |
| 19 | GLU343 | 0.891154 |
| 20 | ALA344 | 0.841199 |
| 21 | TYR345 | 1.15021 |
| 22 | ASN346 | 1.26987 |
| 23 | PRO347 | 1.52808 |
| 24 | SER348 | 1.92538 |
| 25 | ASN349 | 1.97471 |
| 26 | GLY350 | 1.47217 |
| 27 | THR351 | 1.33339 |
| 28 | TRP352 | 1.14772 |
| 29 | LEU353 | 1.1334 |
| 30 | ARG354 | 1.29371 |
| 31 | LEU355 | 1.16674 |
| 32 | ALA356 | 1.18485 |
| 33 | ASP357 | 1.24533 |
| 34 | LEU358 | 0.995318 |
| 35 | GLN359 | 1.3775 |
| 36 | VAL360 | 0.954173 |
| 37 | PRO361 | 0.779157 |
| 38 | ARG362 | 0.684034 |
| 39 | SER363 | 0.597291 |
| 40 | GLY364 | 0.581545 |
| 41 | LEU365 | 0.634723 |
| 42 | ALA366 | 0.791139 |
| 43 | GLY367 | 1.06556 |
| 44 | CYS368 | 1.08011 |
| 45 | VAL369 | 1.58506 |
| 46 | VAL370 | 1.26967 |
| 47 | GLY371 | 1.66967 |
| 48 | GLY372 | 1.54498 |
| 49 | LEU373 | 1.31608 |
| 50 | LEU374 | 1.05542 |
| 51 | TYR375 | 0.819095 |
| 52 | ALA376 | 0.723389 |
| 53 | VAL377 | 0.655183 |
| 54 | GLY378 | 0.641311 |
| 55 | GLY379 | 0.63681 |
| 56 | ARG380 | 1.24204 |
| 57 | ASN381 | 0.707388 |
| 58 | ASN382 | 1.14573 |
| 59 | SER383 | 1.2243 |
| 60 | PRO384 | 1.87062 |
| 61 | ASP385 | 2.23253 |
| 62 | GLY386 | 1.81995 |
| 63 | ASN387 | 2.11134 |
| 64 | THR388 | 2.3191 |
| 65 | ASP389 | 3.54805 |
| 66 | SER390 | 1.38589 |
| 67 | SER391 | 1.03385 |
| 68 | ALA392 | 0.948007 |
| 69 | LEU393 | 0.732003 |
| 70 | ASP394 | 0.990092 |
| 71 | CYS395 | 0.893173 |
| 72 | TYR396 | 1.41871 |
| 73 | ASN397 | 1.65946 |
| 74 | PRO398 | 2.06516 |
| 75 | MET399 | 2.67776 |
| 76 | THR400 | 2.36184 |
| 77 | ASN401 | 2.23951 |
| 78 | GLN402 | 1.9156 |
| 79 | TRP403 | 1.31591 |
| 80 | SER404 | 1.18289 |
| 81 | PRO405 | 1.23428 |
| 82 | CYS406 | 0.976635 |
| 83 | ALA407 | 0.937884 |
| 84 | PRO408 | 0.874083 |
| 85 | MET409 | 0.805566 |
| 86 | SER410 | 1.02121 |
| 87 | VAL411 | 1.24516 |
| 88 | PRO412 | 1.31286 |
| 89 | ARG413 | 0.668993 |
| 90 | ASN414 | 0.785425 |
| 91 | ARG415 | 0.723278 |
| 92 | ILE416 | 0.639319 |
| 93 | GLY417 | 0.654689 |
| 94 | VAL418 | 1.19953 |
| 95 | GLY419 | 0.811182 |
| 96 | VAL420 | 1.01397 |
| 97 | ILE421 | 0.977166 |
| 98 | ASP422 | 1.53027 |
| 99 | GLY423 | 1.01643 |
| 100 | HIS424 | 1.06171 |
| 101 | ILE425 | 0.640261 |
| 102 | TYR426 | 0.625224 |
| 103 | ALA427 | 0.531112 |
| 104 | VAL428 | 0.536688 |
| 105 | GLY429 | 0.540561 |
| 106 | GLY430 | 0.583639 |
| 107 | SER431 | 0.65415 |
| 108 | HIS432 | 1.12037 |
| 109 | GLY433 | 1.03591 |
| 110 | CYS434 | 1.27498 |
| 111 | ILE435 | 0.943782 |
| 112 | HIS436 | 1.00187 |
| 113 | HIS437 | 0.983531 |
| 114 | ASN438 | 0.667089 |
| 115 | SER439 | 0.671024 |
| 116 | VAL440 | 0.535377 |
| 117 | GLU441 | 0.754554 |
| 118 | ARG442 | 0.79918 |
| 119 | TYR443 | 0.913569 |
| 120 | GLU444 | 1.15581 |
| 121 | PRO445 | 1.38986 |
| 122 | GLU446 | 1.75 |
| 123 | ARG447 | 1.9597 |
| 124 | ASP448 | 1.34506 |
| 125 | GLU449 | 1.28945 |
| 126 | TRP450 | 0.762972 |
| 127 | HIS451 | 1.41225 |
| 128 | LEU452 | 0.964783 |
| 129 | VAL453 | 0.732733 |
| 130 | ALA454 | 0.711689 |
| 131 | PRO455 | 0.680302 |
| 132 | MET456 | 0.624417 |
| 133 | LEU457 | 0.780817 |
| 134 | THR458 | 0.615038 |
| 135 | ARG459 | 1.2214 |
| 136 | ARG460 | 0.521858 |
| 137 | ILE461 | 0.661266 |
| 138 | GLY462 | 0.664546 |
| 139 | VAL463 | 0.63174 |
| 140 | GLY464 | 0.641516 |
| 141 | VAL465 | 0.705179 |
| 142 | ALA466 | 0.737225 |
| 143 | VAL467 | 0.966702 |
| 144 | LEU468 | 1.12833 |
| 145 | ASN469 | 1.46335 |
| 146 | ARG470 | 1.48951 |
| 147 | LEU471 | 0.994763 |
| 148 | LEU472 | 0.821476 |
| 149 | TYR473 | 0.859281 |
| 150 | ALA474 | 0.55682 |
| 151 | VAL475 | 0.60922 |
| 152 | GLY476 | 0.544706 |
| 153 | GLY477 | 0.536927 |
| 154 | PHE478 | 0.739428 |
| 155 | ASP479 | 0.974691 |
| 156 | GLY480 | 1.3079 |
| 157 | THR481 | 1.65217 |
| 158 | ASN482 | 1.13536 |
| 159 | ARG483 | 0.746196 |
| 160 | LEU484 | 0.761605 |
| 161 | ASN485 | 0.82734 |
| 162 | SER486 | 0.744764 |
| 163 | ALA487 | 0.621416 |
| 164 | GLU488 | 0.685339 |
| 165 | CYS489 | 0.688237 |
| 166 | TYR490 | 0.67745 |
| 167 | TYR491 | 1.14508 |
| 168 | PRO492 | 1.08071 |
| 169 | GLU493 | 1.61017 |
| 170 | ARG494 | 1.80747 |
| 171 | ASN495 | 0.993121 |
| 172 | GLU496 | 1.14358 |
| 173 | TRP497 | 0.717582 |
| 174 | ARG498 | 1.41369 |
| 175 | MET499 | 1.05288 |
| 176 | ILE500 | 0.851914 |
| 177 | THR501 | 1.03413 |
| 178 | ALA502 | 0.709135 |
| 179 | MET503 | 0.803086 |
| 180 | ASN504 | 0.875148 |
| 181 | THR505 | 0.655811 |
| 182 | ILE506 | 0.813934 |
| 183 | ARG507 | 0.545325 |
| 184 | SER508 | 0.538756 |
| 185 | GLY509 | 0.52584 |
| 186 | ALA510 | 0.536099 |
| 187 | GLY511 | 0.551645 |
| 188 | VAL512 | 0.651433 |
| 189 | CYS513 | 0.672857 |
| 190 | VAL514 | 0.81518 |
| 191 | LEU515 | 1.02038 |
| 192 | HIS516 | 1.56687 |
| 193 | ASN517 | 1.15127 |
| 194 | CYS518 | 0.799586 |
| 195 | ILE519 | 0.745396 |
| 196 | TYR520 | 0.845157 |
| 197 | ALA521 | 0.598166 |
| 198 | ALA522 | 0.521239 |
| 199 | GLY523 | 0.521222 |
| 200 | GLY524 | 0.5382 |
| 201 | TYR525 | 0.693225 |
| 202 | ASP526 | 0.936588 |
| 203 | GLY527 | 1.32544 |
| 204 | GLN528 | 2.34372 |
| 205 | ASP529 | 1.20918 |
| 206 | GLN530 | 0.830647 |
| 207 | LEU531 | 0.620013 |
| 208 | ASN532 | 0.58867 |
| 209 | SER533 | 0.64277 |
| 210 | VAL534 | 0.54996 |
| 211 | GLU535 | 0.710079 |
| 212 | ARG536 | 0.872172 |
| 213 | TYR537 | 1.00696 |
| 214 | ASP538 | 1.25546 |
| 215 | VAL539 | 1.62382 |
| 216 | ALA540 | 1.82154 |
| 217 | THR541 | 1.49522 |
| 218 | ALA542 | 1.36654 |
| 219 | THR543 | 1.1529 |
| 220 | TRP544 | 0.886461 |
| 221 | THR545 | 0.832072 |
| 222 | PHE546 | 1.74702 |
| 223 | VAL547 | 0.88339 |
| 224 | ALA548 | 0.820696 |
| 225 | PRO549 | 0.778286 |
| 226 | MET550 | 0.617947 |
| 227 | LYS551 | 1.17567 |
| 228 | HIS552 | 0.741888 |
| 229 | ARG553 | 0.897869 |
| 230 | ARG554 | 0.570375 |
| 231 | SER555 | 0.60581 |
| 232 | ALA556 | 0.578073 |
| 233 | LEU557 | 0.567288 |
| 234 | GLY558 | 0.750383 |
| 235 | ILE559 | 0.661062 |
| 236 | THR560 | 0.865089 |
| 237 | VAL561 | 1.04246 |
| 238 | HIS562 | 1.21867 |
| 239 | GLN563 | 1.71435 |
| 240 | GLY564 | 1.16224 |
| 241 | ARG565 | 1.0632 |
| 242 | ILE566 | 0.848 |
| 243 | TYR567 | 0.717254 |
| 244 | VAL568 | 0.575388 |
| 245 | LEU569 | 0.827796 |
| 246 | GLY570 | 0.620902 |
| 247 | GLY571 | 0.587924 |
| 248 | TYR572 | 0.916464 |
| 249 | ASP573 | 1.16091 |
| 250 | GLY574 | 1.37865 |
| 251 | HIS575 | 2.18581 |
| 252 | THR576 | 1.17792 |
| 253 | PHE577 | 0.841043 |
| 254 | LEU578 | 0.765081 |
| 255 | ASP579 | 0.838695 |
| 256 | SER580 | 0.710853 |
| 257 | VAL581 | 0.598903 |
| 258 | GLU582 | 0.597585 |
| 259 | CYS583 | 0.737594 |
| 260 | TYR584 | 1.06278 |
| 261 | ASP585 | 1.41844 |
| 262 | PRO586 | 1.62553 |
| 263 | ASP587 | 1.98818 |
| 264 | THR588 | 1.68622 |
| 265 | ASP589 | 1.77205 |
| 266 | THR590 | 1.552 |
| 267 | TRP591 | 0.928868 |
| 268 | SER592 | 1.0263 |
| 269 | GLU593 | 1.31635 |
| 270 | VAL594 | 1.00166 |
| 271 | THR595 | 1.22707 |
| 272 | ARG596 | 1.30768 |
| 273 | MET597 | 0.761454 |
| 274 | THR598 | 0.906359 |
| 275 | SER599 | 1.00432 |
| 276 | GLY600 | 0.766313 |
| 277 | ARG601 | 0.91396 |
| 278 | SER602 | 0.638271 |
| 279 | GLY603 | 0.649942 |
| 280 | VAL604 | 0.773905 |
| 281 | GLY605 | 0.727769 |
| 282 | VAL606 | 0.930415 |
| 283 | ALA607 | 1.19747 |
| 284 | VAL608 | 3.13645 |
| 285 | THR609 | 3.79079 |

**Table S8.** RMSF of the best conformation pose of compound **5ab** in in the protein of PDB: 4IQK.

| Entry | Item | RMSF |
| --- | --- | --- |
| 1 | GLY325 | 2.83947 |
| 2 | ARG326 | 3.03317 |
| 3 | LEU327 | 1.19197 |
| 4 | ILE328 | 0.903073 |
| 5 | TYR329 | 1.15178 |
| 6 | THR330 | 1.10015 |
| 7 | ALA331 | 0.867061 |
| 8 | GLY332 | 0.660884 |
| 9 | GLY333 | 0.53141 |
| 10 | TYR334 | 0.699964 |
| 11 | PHE335 | 0.868419 |
| 12 | ARG336 | 1.59093 |
| 13 | GLN337 | 0.901679 |
| 14 | SER338 | 0.511126 |
| 15 | LEU339 | 0.731259 |
| 16 | SER340 | 0.733553 |
| 17 | TYR341 | 1.16499 |
| 18 | LEU342 | 1.07471 |
| 19 | GLU343 | 1.12585 |
| 20 | ALA344 | 0.852285 |
| 21 | TYR345 | 1.04174 |
| 22 | ASN346 | 1.17817 |
| 23 | PRO347 | 1.52757 |
| 24 | SER348 | 1.85096 |
| 25 | ASN349 | 1.62327 |
| 26 | GLY350 | 1.31962 |
| 27 | THR351 | 1.20679 |
| 28 | TRP352 | 0.998961 |
| 29 | LEU353 | 1.08871 |
| 30 | ARG354 | 1.53679 |
| 31 | LEU355 | 1.14181 |
| 32 | ALA356 | 1.02147 |
| 33 | ASP357 | 1.16497 |
| 34 | LEU358 | 0.843388 |
| 35 | GLN359 | 1.08815 |
| 36 | VAL360 | 0.717435 |
| 37 | PRO361 | 0.613674 |
| 38 | ARG362 | 0.57344 |
| 39 | SER363 | 0.511711 |
| 40 | GLY364 | 0.50946 |
| 41 | LEU365 | 0.59707 |
| 42 | ALA366 | 0.638638 |
| 43 | GLY367 | 0.868159 |
| 44 | CYS368 | 0.952011 |
| 45 | VAL369 | 1.40672 |
| 46 | VAL370 | 1.43954 |
| 47 | GLY371 | 1.95122 |
| 48 | GLY372 | 1.4162 |
| 49 | LEU373 | 1.3011 |
| 50 | LEU374 | 0.944325 |
| 51 | TYR375 | 0.99539 |
| 52 | ALA376 | 0.649441 |
| 53 | VAL377 | 0.610125 |
| 54 | GLY378 | 0.555675 |
| 55 | GLY379 | 0.557419 |
| 56 | ARG380 | 1.02461 |
| 57 | ASN381 | 0.7407 |
| 58 | ASN382 | 1.00098 |
| 59 | SER383 | 1.03544 |
| 60 | PRO384 | 1.71445 |
| 61 | ASP385 | 2.25156 |
| 62 | GLY386 | 1.55706 |
| 63 | ASN387 | 1.51814 |
| 64 | THR388 | 1.66383 |
| 65 | ASP389 | 2.28978 |
| 66 | SER390 | 0.968676 |
| 67 | SER391 | 1.21294 |
| 68 | ALA392 | 1.07499 |
| 69 | LEU393 | 0.980242 |
| 70 | ASP394 | 1.04488 |
| 71 | CYS395 | 0.900906 |
| 72 | TYR396 | 1.01825 |
| 73 | ASN397 | 1.27098 |
| 74 | PRO398 | 1.52737 |
| 75 | MET399 | 2.11354 |
| 76 | THR400 | 1.7357 |
| 77 | ASN401 | 1.6866 |
| 78 | GLN402 | 1.68929 |
| 79 | TRP403 | 1.0301 |
| 80 | SER404 | 1.07254 |
| 81 | PRO405 | 1.11315 |
| 82 | CYS406 | 1.15639 |
| 83 | ALA407 | 1.08528 |
| 84 | PRO408 | 1.19261 |
| 85 | MET409 | 1.13084 |
| 86 | SER410 | 1.24851 |
| 87 | VAL411 | 1.25156 |
| 88 | PRO412 | 1.20657 |
| 89 | ARG413 | 0.62019 |
| 90 | ASN414 | 0.543591 |
| 91 | ARG415 | 0.690361 |
| 92 | ILE416 | 0.572927 |
| 93 | GLY417 | 0.575622 |
| 94 | VAL418 | 0.664126 |
| 95 | GLY419 | 0.735579 |
| 96 | VAL420 | 0.966131 |
| 97 | ILE421 | 1.31052 |
| 98 | ASP422 | 1.6088 |
| 99 | GLY423 | 1.29564 |
| 100 | HIS424 | 1.18089 |
| 101 | ILE425 | 0.773561 |
| 102 | TYR426 | 0.70067 |
| 103 | ALA427 | 0.544023 |
| 104 | VAL428 | 0.540326 |
| 105 | GLY429 | 0.600742 |
| 106 | GLY430 | 0.590324 |
| 107 | SER431 | 0.584506 |
| 108 | HIS432 | 1.01017 |
| 109 | GLY433 | 0.882725 |
| 110 | CYS434 | 1.22059 |
| 111 | ILE435 | 1.08344 |
| 112 | HIS436 | 1.2276 |
| 113 | HIS437 | 1.06445 |
| 114 | ASN438 | 0.685438 |
| 115 | SER439 | 0.902268 |
| 116 | VAL440 | 0.577329 |
| 117 | GLU441 | 0.846184 |
| 118 | ARG442 | 0.867864 |
| 119 | TYR443 | 1.08151 |
| 120 | GLU444 | 1.25423 |
| 121 | PRO445 | 1.63522 |
| 122 | GLU446 | 2.11935 |
| 123 | ARG447 | 2.13703 |
| 124 | ASP448 | 1.68604 |
| 125 | GLU449 | 1.62536 |
| 126 | TRP450 | 0.776601 |
| 127 | HIS451 | 1.37381 |
| 128 | LEU452 | 1.40877 |
| 129 | VAL453 | 1.04772 |
| 130 | ALA454 | 0.79901 |
| 131 | PRO455 | 0.763472 |
| 132 | MET456 | 0.579296 |
| 133 | LEU457 | 0.948177 |
| 134 | THR458 | 0.698503 |
| 135 | ARG459 | 1.34034 |
| 136 | ARG460 | 0.622496 |
| 137 | ILE461 | 0.779868 |
| 138 | GLY462 | 0.697033 |
| 139 | VAL463 | 0.646514 |
| 140 | GLY464 | 0.641292 |
| 141 | VAL465 | 0.663364 |
| 142 | ALA466 | 0.665438 |
| 143 | VAL467 | 0.942487 |
| 144 | LEU468 | 1.04403 |
| 145 | ASN469 | 1.26457 |
| 146 | ARG470 | 1.76913 |
| 147 | LEU471 | 0.984046 |
| 148 | LEU472 | 0.851694 |
| 149 | TYR473 | 0.725228 |
| 150 | ALA474 | 0.549794 |
| 151 | VAL475 | 0.591561 |
| 152 | GLY476 | 0.579497 |
| 153 | GLY477 | 0.60123 |
| 154 | PHE478 | 0.700657 |
| 155 | ASP479 | 0.907466 |
| 156 | GLY480 | 1.15258 |
| 157 | THR481 | 1.78221 |
| 158 | ASN482 | 1.37994 |
| 159 | ARG483 | 0.953865 |
| 160 | LEU484 | 0.772978 |
| 161 | ASN485 | 0.692183 |
| 162 | SER486 | 0.709233 |
| 163 | ALA487 | 0.594694 |
| 164 | GLU488 | 0.701357 |
| 165 | CYS489 | 0.634294 |
| 166 | TYR490 | 0.877989 |
| 167 | TYR491 | 1.28939 |
| 168 | PRO492 | 1.45471 |
| 169 | GLU493 | 1.86842 |
| 170 | ARG494 | 1.91724 |
| 171 | ASN495 | 1.42421 |
| 172 | GLU496 | 1.36184 |
| 173 | TRP497 | 0.664078 |
| 174 | ARG498 | 1.25466 |
| 175 | MET499 | 1.06422 |
| 176 | ILE500 | 0.809876 |
| 177 | THR501 | 0.906402 |
| 178 | ALA502 | 0.684995 |
| 179 | MET503 | 0.682827 |
| 180 | ASN504 | 0.924037 |
| 181 | THR505 | 0.598822 |
| 182 | ILE506 | 0.934684 |
| 183 | ARG507 | 0.580242 |
| 184 | SER508 | 0.718034 |
| 185 | GLY509 | 0.678347 |
| 186 | ALA510 | 0.768855 |
| 187 | GLY511 | 0.662779 |
| 188 | VAL512 | 1.02302 |
| 189 | CYS513 | 1.06428 |
| 190 | VAL514 | 0.954559 |
| 191 | LEU515 | 1.13515 |
| 192 | HIS516 | 2.21878 |
| 193 | ASN517 | 1.48912 |
| 194 | CYS518 | 0.988448 |
| 195 | ILE519 | 0.83958 |
| 196 | TYR520 | 0.691628 |
| 197 | ALA521 | 0.511327 |
| 198 | ALA522 | 0.545377 |
| 199 | GLY523 | 0.544627 |
| 200 | GLY524 | 0.542235 |
| 201 | TYR525 | 0.897418 |
| 202 | ASP526 | 1.12394 |
| 203 | GLY527 | 1.22197 |
| 204 | GLN528 | 1.81008 |
| 205 | ASP529 | 1.281 |
| 206 | GLN530 | 0.945519 |
| 207 | LEU531 | 0.689195 |
| 208 | ASN532 | 0.598145 |
| 209 | SER533 | 0.674666 |
| 210 | VAL534 | 0.541579 |
| 211 | GLU535 | 0.670622 |
| 212 | ARG536 | 0.927942 |
| 213 | TYR537 | 1.0457 |
| 214 | ASP538 | 1.19008 |
| 215 | VAL539 | 1.6051 |
| 216 | ALA540 | 1.78713 |
| 217 | THR541 | 1.58734 |
| 218 | ALA542 | 1.4057 |
| 219 | THR543 | 1.09948 |
| 220 | TRP544 | 0.764289 |
| 221 | THR545 | 0.790268 |
| 222 | PHE546 | 0.854514 |
| 223 | VAL547 | 0.828628 |
| 224 | ALA548 | 0.711755 |
| 225 | PRO549 | 0.727297 |
| 226 | MET550 | 0.612482 |
| 227 | LYS551 | 1.37237 |
| 228 | HIS552 | 0.714631 |
| 229 | ARG553 | 0.977492 |
| 230 | ARG554 | 0.545454 |
| 231 | SER555 | 0.587222 |
| 232 | ALA556 | 0.564003 |
| 233 | LEU557 | 0.601911 |
| 234 | GLY558 | 0.6396 |
| 235 | ILE559 | 0.761023 |
| 236 | THR560 | 1.00691 |
| 237 | VAL561 | 0.778466 |
| 238 | HIS562 | 1.11308 |
| 239 | GLN563 | 1.92259 |
| 240 | GLY564 | 1.06078 |
| 241 | ARG565 | 1.54552 |
| 242 | ILE566 | 0.673643 |
| 243 | TYR567 | 0.673216 |
| 244 | VAL568 | 0.538436 |
| 245 | LEU569 | 0.856934 |
| 246 | GLY570 | 0.586923 |
| 247 | GLY571 | 0.553655 |
| 248 | TYR572 | 0.863055 |
| 249 | ASP573 | 1.27393 |
| 250 | GLY574 | 1.33682 |
| 251 | HIS575 | 1.87708 |
| 252 | THR576 | 1.21388 |
| 253 | PHE577 | 0.761754 |
| 254 | LEU578 | 0.631821 |
| 255 | ASP579 | 0.741772 |
| 256 | SER580 | 0.544671 |
| 257 | VAL581 | 0.518451 |
| 258 | GLU582 | 0.520909 |
| 259 | CYS583 | 0.628478 |
| 260 | TYR584 | 1.01747 |
| 261 | ASP585 | 1.46912 |
| 262 | PRO586 | 1.66647 |
| 263 | ASP587 | 2.19512 |
| 264 | THR588 | 1.7511 |
| 265 | ASP589 | 1.66435 |
| 266 | THR590 | 1.18811 |
| 267 | TRP591 | 0.728273 |
| 268 | SER592 | 0.840679 |
| 269 | GLU593 | 1.18095 |
| 270 | VAL594 | 0.766242 |
| 271 | THR595 | 0.791345 |
| 272 | ARG596 | 1.33114 |
| 273 | MET597 | 0.750856 |
| 274 | THR598 | 0.762804 |
| 275 | SER599 | 0.752811 |
| 276 | GLY600 | 0.6099 |
| 277 | ARG601 | 0.68075 |
| 278 | SER602 | 0.547082 |
| 279 | GLY603 | 0.54373 |
| 280 | VAL604 | 0.822849 |
| 281 | GLY605 | 1.27515 |
| 282 | VAL606 | 1.36979 |
| 283 | ALA607 | 2.96366 |
| 284 | VAL608 | 4.11098 |
| 285 | THR609 | 3.92339 |

**Table S9.** RMSF of the best conformation pose of compound **5ac** in in the protein of PDB: 4IQK.

| Entry | Item | RMSF |
| --- | --- | --- |
| 1 | GLY325 | 2.4721 |
| 2 | ARG326 | 2.53413 |
| 3 | LEU327 | 1.12235 |
| 4 | ILE328 | 0.762624 |
| 5 | TYR329 | 0.91406 |
| 6 | THR330 | 0.647104 |
| 7 | ALA331 | 0.599795 |
| 8 | GLY332 | 0.662292 |
| 9 | GLY333 | 0.538525 |
| 10 | TYR334 | 0.80667 |
| 11 | PHE335 | 0.844427 |
| 12 | ARG336 | 1.51727 |
| 13 | GLN337 | 0.990598 |
| 14 | SER338 | 0.561545 |
| 15 | LEU339 | 0.615422 |
| 16 | SER340 | 0.760259 |
| 17 | TYR341 | 1.04085 |
| 18 | LEU342 | 0.699991 |
| 19 | GLU343 | 0.782585 |
| 20 | ALA344 | 0.723224 |
| 21 | TYR345 | 0.929815 |
| 22 | ASN346 | 1.05964 |
| 23 | PRO347 | 1.38577 |
| 24 | SER348 | 1.60211 |
| 25 | ASN349 | 1.60653 |
| 26 | GLY350 | 1.24871 |
| 27 | THR351 | 1.12251 |
| 28 | TRP352 | 0.822242 |
| 29 | LEU353 | 1.09937 |
| 30 | ARG354 | 1.5336 |
| 31 | LEU355 | 1.07433 |
| 32 | ALA356 | 1.07871 |
| 33 | ASP357 | 1.10048 |
| 34 | LEU358 | 0.910668 |
| 35 | GLN359 | 1.52023 |
| 36 | VAL360 | 0.939745 |
| 37 | PRO361 | 0.613271 |
| 38 | ARG362 | 0.619019 |
| 39 | SER363 | 0.603501 |
| 40 | GLY364 | 0.539756 |
| 41 | LEU365 | 0.579922 |
| 42 | ALA366 | 0.649887 |
| 43 | GLY367 | 0.810594 |
| 44 | CYS368 | 0.950461 |
| 45 | VAL369 | 1.32012 |
| 46 | VAL370 | 1.24701 |
| 47 | GLY371 | 1.9827 |
| 48 | GLY372 | 1.5868 |
| 49 | LEU373 | 1.33299 |
| 50 | LEU374 | 1.08733 |
| 51 | TYR375 | 1.04426 |
| 52 | ALA376 | 0.605605 |
| 53 | VAL377 | 0.640341 |
| 54 | GLY378 | 0.564131 |
| 55 | GLY379 | 0.564071 |
| 56 | ARG380 | 0.981937 |
| 57 | ASN381 | 0.896273 |
| 58 | ASN382 | 1.30214 |
| 59 | SER383 | 1.42348 |
| 60 | PRO384 | 2.07802 |
| 61 | ASP385 | 2.31174 |
| 62 | GLY386 | 1.51163 |
| 63 | ASN387 | 1.3417 |
| 64 | THR388 | 1.34065 |
| 65 | ASP389 | 1.51597 |
| 66 | SER390 | 1.29556 |
| 67 | SER391 | 1.17793 |
| 68 | ALA392 | 0.852677 |
| 69 | LEU393 | 0.934703 |
| 70 | ASP394 | 0.886648 |
| 71 | CYS395 | 0.823821 |
| 72 | TYR396 | 1.25015 |
| 73 | ASN397 | 1.79045 |
| 74 | PRO398 | 2.25662 |
| 75 | MET399 | 3.03753 |
| 76 | THR400 | 2.34427 |
| 77 | ASN401 | 1.91441 |
| 78 | GLN402 | 1.76382 |
| 79 | TRP403 | 0.936394 |
| 80 | SER404 | 1.01444 |
| 81 | PRO405 | 1.04297 |
| 82 | CYS406 | 0.967243 |
| 83 | ALA407 | 0.968455 |
| 84 | PRO408 | 0.789853 |
| 85 | MET409 | 0.755789 |
| 86 | SER410 | 0.668502 |
| 87 | VAL411 | 0.726702 |
| 88 | PRO412 | 0.788023 |
| 89 | ARG413 | 0.540963 |
| 90 | ASN414 | 0.607014 |
| 91 | ARG415 | 0.692867 |
| 92 | ILE416 | 0.580507 |
| 93 | GLY417 | 0.685828 |
| 94 | VAL418 | 1.15833 |
| 95 | GLY419 | 0.838028 |
| 96 | VAL420 | 1.41713 |
| 97 | ILE421 | 1.35869 |
| 98 | ASP422 | 1.87973 |
| 99 | GLY423 | 1.31777 |
| 100 | HIS424 | 1.312 |
| 101 | ILE425 | 1.06201 |
| 102 | TYR426 | 0.686627 |
| 103 | ALA427 | 0.513841 |
| 104 | VAL428 | 0.518112 |
| 105 | GLY429 | 0.550591 |
| 106 | GLY430 | 0.495163 |
| 107 | SER431 | 0.531088 |
| 108 | HIS432 | 1.05327 |
| 109 | GLY433 | 0.812726 |
| 110 | CYS434 | 0.951505 |
| 111 | ILE435 | 0.909062 |
| 112 | HIS436 | 0.883364 |
| 113 | HIS437 | 0.827235 |
| 114 | ASN438 | 0.569268 |
| 115 | SER439 | 0.789074 |
| 116 | VAL440 | 0.566333 |
| 117 | GLU441 | 0.721246 |
| 118 | ARG442 | 1.0268 |
| 119 | TYR443 | 1.17491 |
| 120 | GLU444 | 1.49652 |
| 121 | PRO445 | 1.90896 |
| 122 | GLU446 | 2.37754 |
| 123 | ARG447 | 2.39194 |
| 124 | ASP448 | 2.3454 |
| 125 | GLU449 | 1.8126 |
| 126 | TRP450 | 0.980076 |
| 127 | HIS451 | 1.48498 |
| 128 | LEU452 | 1.31883 |
| 129 | VAL453 | 0.850814 |
| 130 | ALA454 | 0.726082 |
| 131 | PRO455 | 0.702594 |
| 132 | MET456 | 0.50482 |
| 133 | LEU457 | 0.731854 |
| 134 | THR458 | 0.545495 |
| 135 | ARG459 | 0.710561 |
| 136 | ARG460 | 0.483158 |
| 137 | ILE461 | 0.614407 |
| 138 | GLY462 | 0.682288 |
| 139 | VAL463 | 0.669658 |
| 140 | GLY464 | 0.842836 |
| 141 | VAL465 | 0.957811 |
| 142 | ALA466 | 0.764879 |
| 143 | VAL467 | 0.871592 |
| 144 | LEU468 | 1.0597 |
| 145 | ASN469 | 1.31064 |
| 146 | ARG470 | 1.84756 |
| 147 | LEU471 | 1.00026 |
| 148 | LEU472 | 0.988491 |
| 149 | TYR473 | 0.864198 |
| 150 | ALA474 | 0.523593 |
| 151 | VAL475 | 0.562837 |
| 152 | GLY476 | 0.551292 |
| 153 | GLY477 | 0.601267 |
| 154 | PHE478 | 0.755385 |
| 155 | ASP479 | 0.725888 |
| 156 | GLY480 | 1.069 |
| 157 | THR481 | 1.35046 |
| 158 | ASN482 | 0.977071 |
| 159 | ARG483 | 1.20634 |
| 160 | LEU484 | 0.571422 |
| 161 | ASN485 | 0.540522 |
| 162 | SER486 | 0.577465 |
| 163 | ALA487 | 0.503085 |
| 164 | GLU488 | 0.642751 |
| 165 | CYS489 | 0.608898 |
| 166 | TYR490 | 0.933683 |
| 167 | TYR491 | 1.26468 |
| 168 | PRO492 | 1.37234 |
| 169 | GLU493 | 1.97907 |
| 170 | ARG494 | 2.04863 |
| 171 | ASN495 | 1.48966 |
| 172 | GLU496 | 1.38077 |
| 173 | TRP497 | 0.693095 |
| 174 | ARG498 | 1.41058 |
| 175 | MET499 | 0.963113 |
| 176 | ILE500 | 0.733372 |
| 177 | THR501 | 0.802557 |
| 178 | ALA502 | 0.604522 |
| 179 | MET503 | 0.522571 |
| 180 | ASN504 | 0.762892 |
| 181 | THR505 | 0.598753 |
| 182 | ILE506 | 0.665224 |
| 183 | ARG507 | 0.536008 |
| 184 | SER508 | 0.567064 |
| 185 | GLY509 | 0.567976 |
| 186 | ALA510 | 0.573356 |
| 187 | GLY511 | 0.531379 |
| 188 | VAL512 | 0.613805 |
| 189 | CYS513 | 0.769609 |
| 190 | VAL514 | 0.851321 |
| 191 | LEU515 | 0.956214 |
| 192 | HIS516 | 1.6543 |
| 193 | ASN517 | 1.39761 |
| 194 | CYS518 | 0.830541 |
| 195 | ILE519 | 0.662858 |
| 196 | TYR520 | 0.668595 |
| 197 | ALA521 | 0.550036 |
| 198 | ALA522 | 0.518452 |
| 199 | GLY523 | 0.529966 |
| 200 | GLY524 | 0.570512 |
| 201 | TYR525 | 1.00905 |
| 202 | ASP526 | 1.14967 |
| 203 | GLY527 | 1.17705 |
| 204 | GLN528 | 2.00369 |
| 205 | ASP529 | 1.2908 |
| 206 | GLN530 | 0.985721 |
| 207 | LEU531 | 0.618572 |
| 208 | ASN532 | 0.537195 |
| 209 | SER533 | 0.645588 |
| 210 | VAL534 | 0.528615 |
| 211 | GLU535 | 0.540482 |
| 212 | ARG536 | 0.739129 |
| 213 | TYR537 | 0.982063 |
| 214 | ASP538 | 1.09126 |
| 215 | VAL539 | 1.49082 |
| 216 | ALA540 | 1.67468 |
| 217 | THR541 | 1.48016 |
| 218 | ALA542 | 1.32344 |
| 219 | THR543 | 1.08266 |
| 220 | TRP544 | 0.70442 |
| 221 | THR545 | 0.698412 |
| 222 | PHE546 | 0.999192 |
| 223 | VAL547 | 0.948521 |
| 224 | ALA548 | 0.66344 |
| 225 | PRO549 | 0.720126 |
| 226 | MET550 | 0.653835 |
| 227 | LYS551 | 1.04281 |
| 228 | HIS552 | 0.703314 |
| 229 | ARG553 | 0.89143 |
| 230 | ARG554 | 0.606912 |
| 231 | SER555 | 0.521921 |
| 232 | ALA556 | 0.566561 |
| 233 | LEU557 | 0.590406 |
| 234 | GLY558 | 0.660099 |
| 235 | ILE559 | 0.619926 |
| 236 | THR560 | 0.850829 |
| 237 | VAL561 | 0.830724 |
| 238 | HIS562 | 1.19843 |
| 239 | GLN563 | 1.85518 |
| 240 | GLY564 | 1.00157 |
| 241 | ARG565 | 1.41953 |
| 242 | ILE566 | 0.592241 |
| 243 | TYR567 | 0.633468 |
| 244 | VAL568 | 0.516974 |
| 245 | LEU569 | 0.721517 |
| 246 | GLY570 | 0.571281 |
| 247 | GLY571 | 0.60465 |
| 248 | TYR572 | 1.41794 |
| 249 | ASP573 | 1.45297 |
| 250 | GLY574 | 1.63605 |
| 251 | HIS575 | 2.31344 |
| 252 | THR576 | 1.33202 |
| 253 | PHE577 | 1.11434 |
| 254 | LEU578 | 0.755131 |
| 255 | ASP579 | 0.853255 |
| 256 | SER580 | 0.693531 |
| 257 | VAL581 | 0.56973 |
| 258 | GLU582 | 0.548011 |
| 259 | CYS583 | 0.625873 |
| 260 | TYR584 | 0.83119 |
| 261 | ASP585 | 1.17447 |
| 262 | PRO586 | 1.29604 |
| 263 | ASP587 | 1.67112 |
| 264 | THR588 | 1.4992 |
| 265 | ASP589 | 1.36628 |
| 266 | THR590 | 1.01926 |
| 267 | TRP591 | 0.724602 |
| 268 | SER592 | 0.841777 |
| 269 | GLU593 | 1.11355 |
| 270 | VAL594 | 0.87724 |
| 271 | THR595 | 1.10072 |
| 272 | ARG596 | 1.38625 |
| 273 | MET597 | 0.822255 |
| 274 | THR598 | 1.45339 |
| 275 | SER599 | 0.931356 |
| 276 | GLY600 | 0.804378 |
| 277 | ARG601 | 0.731601 |
| 278 | SER602 | 0.63367 |
| 279 | GLY603 | 0.579749 |
| 280 | VAL604 | 0.717045 |
| 281 | GLY605 | 0.686723 |
| 282 | VAL606 | 0.977311 |
| 283 | ALA607 | 1.30788 |
| 284 | VAL608 | 3.24464 |
| 285 | THR609 | 3.88858 |


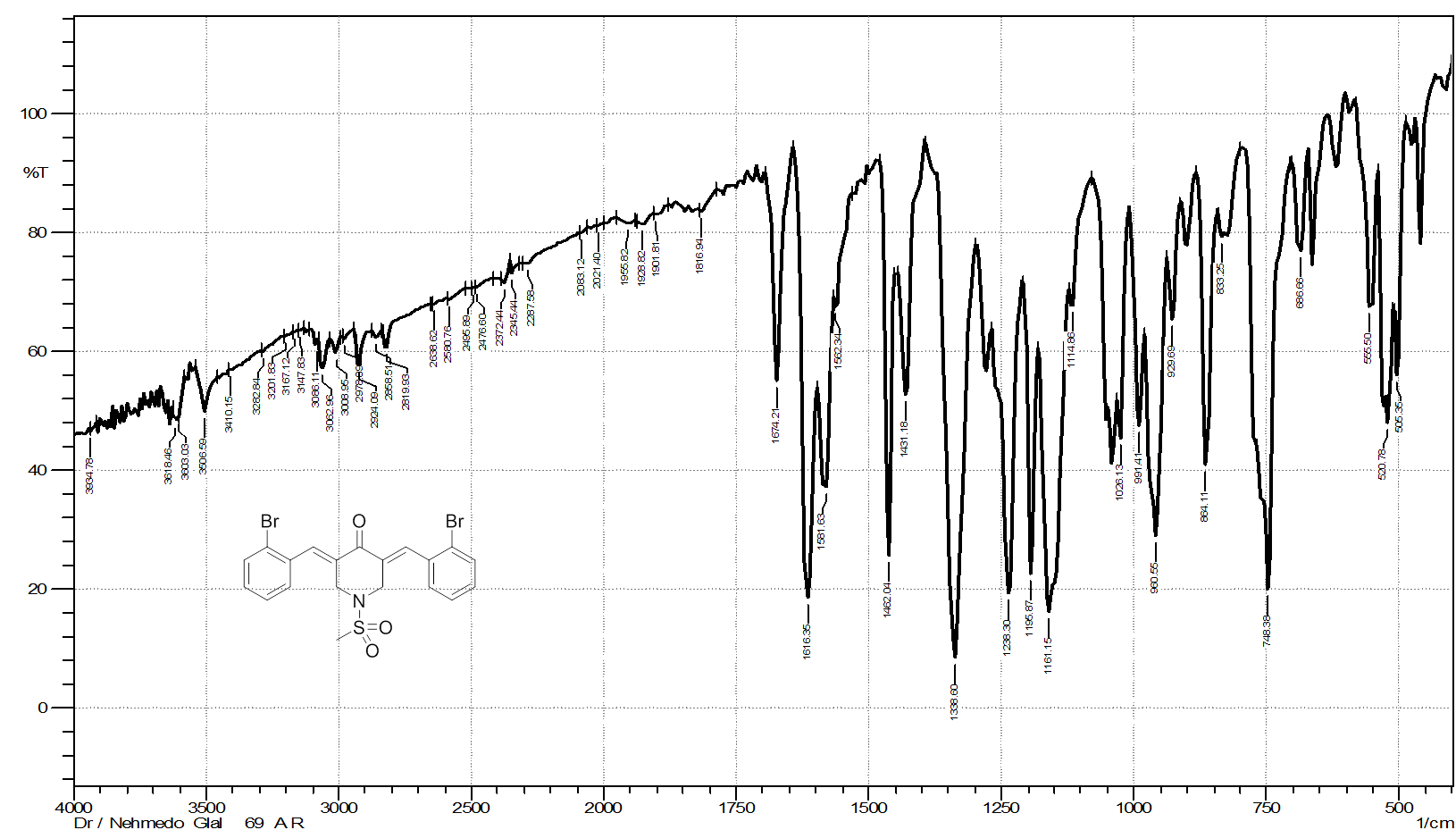


**Fig. S1.** IR spectrum of compound **5w** (KBr pellet).


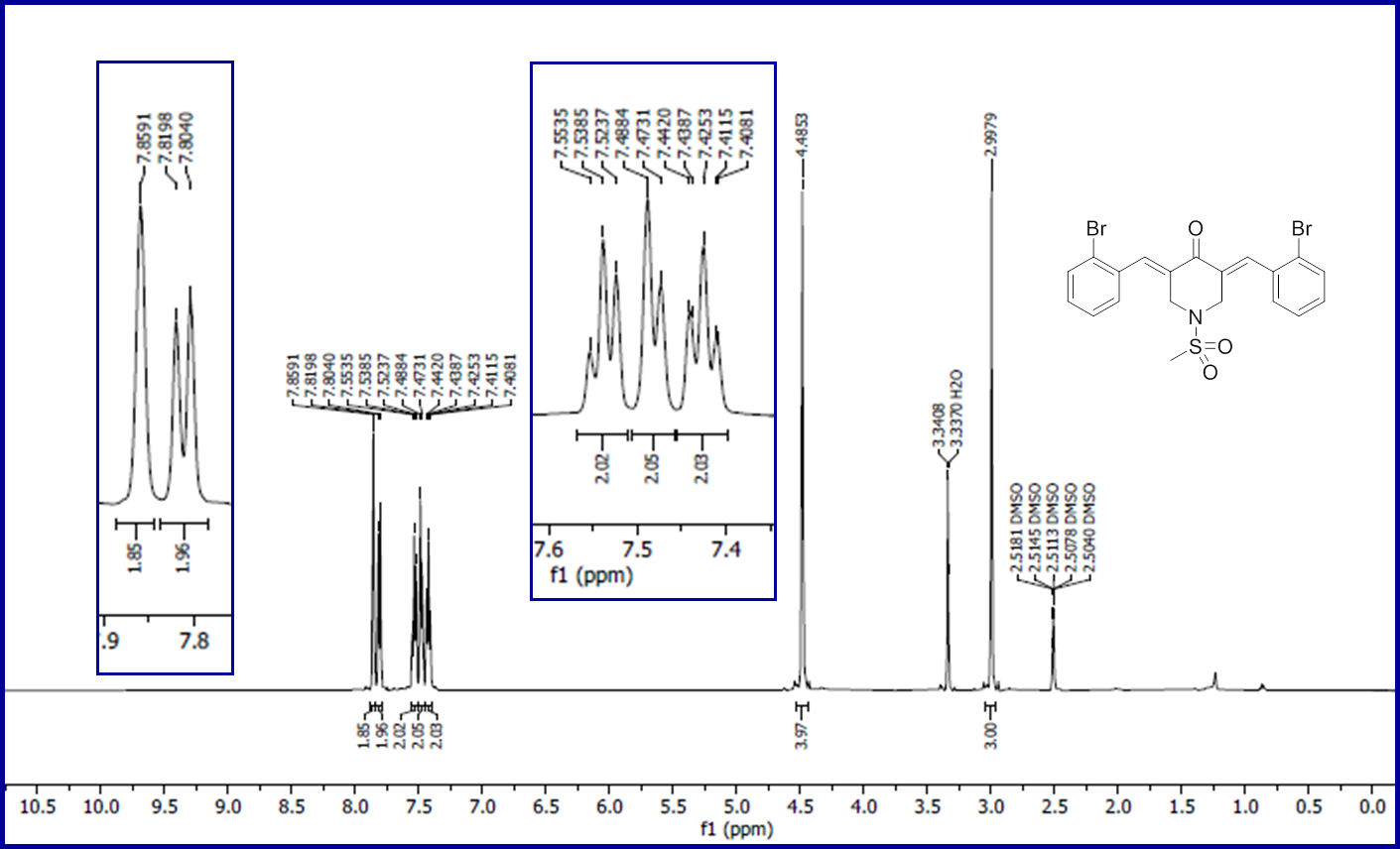


**Fig. S2.** ^1^H-NMR spectrum of compound **5w** in DMSO-*d_6_*.


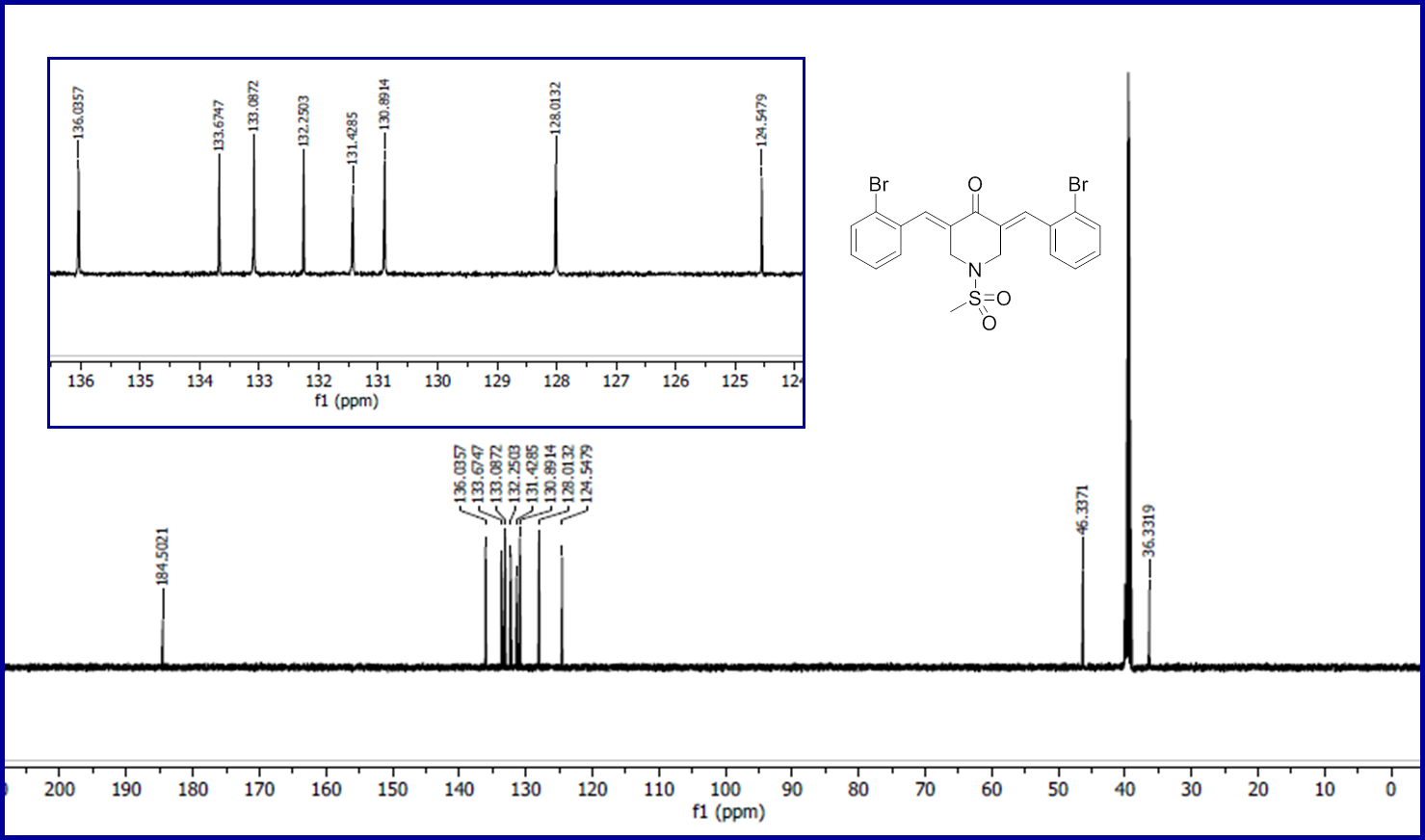


**Fig. S3.** ^13^C-NMR spectrum of compound **5w** in DMSO-*d_6_*.


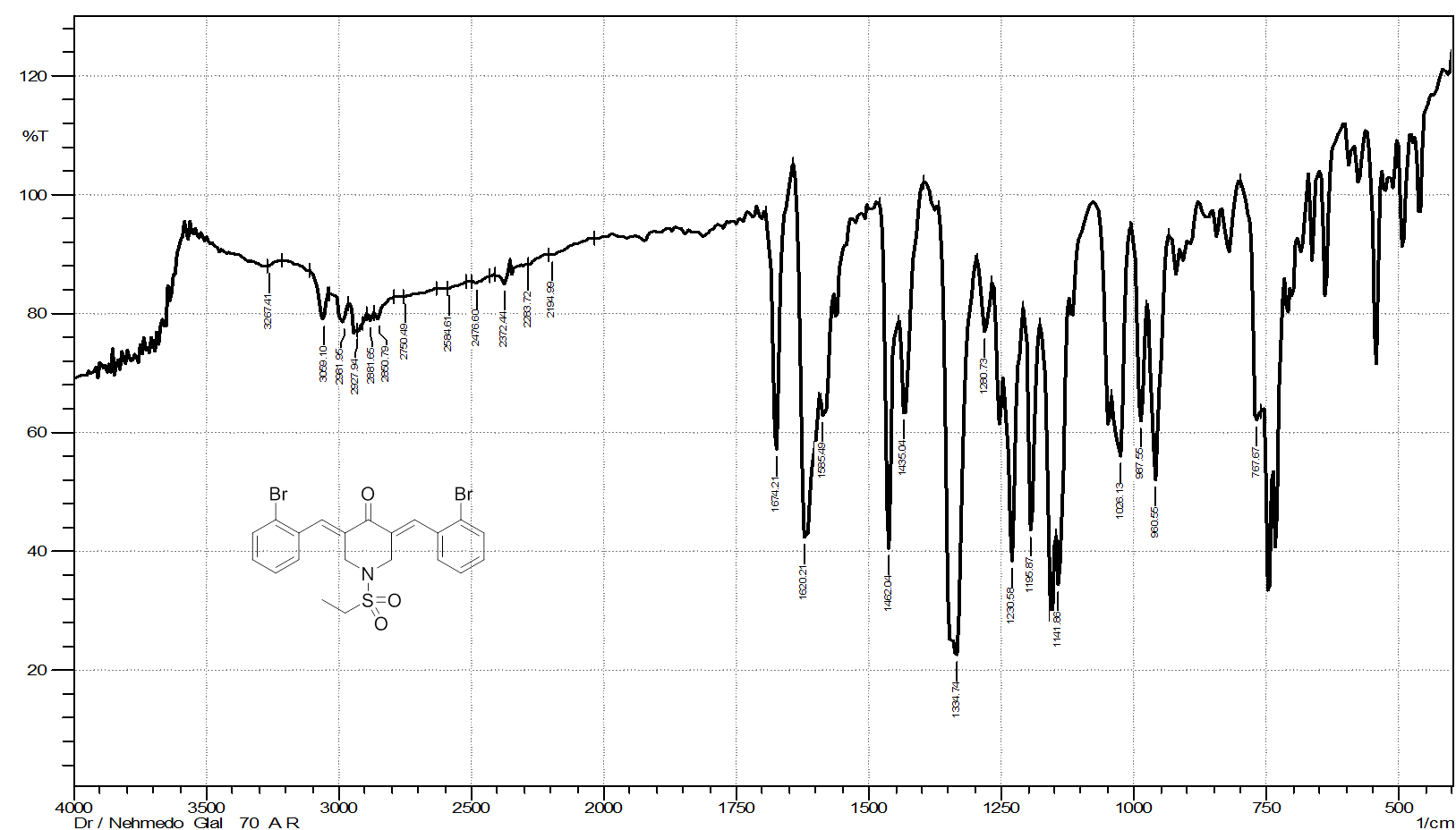


**Fig. S4.** IR spectrum of compound **5x** (KBr pellet).


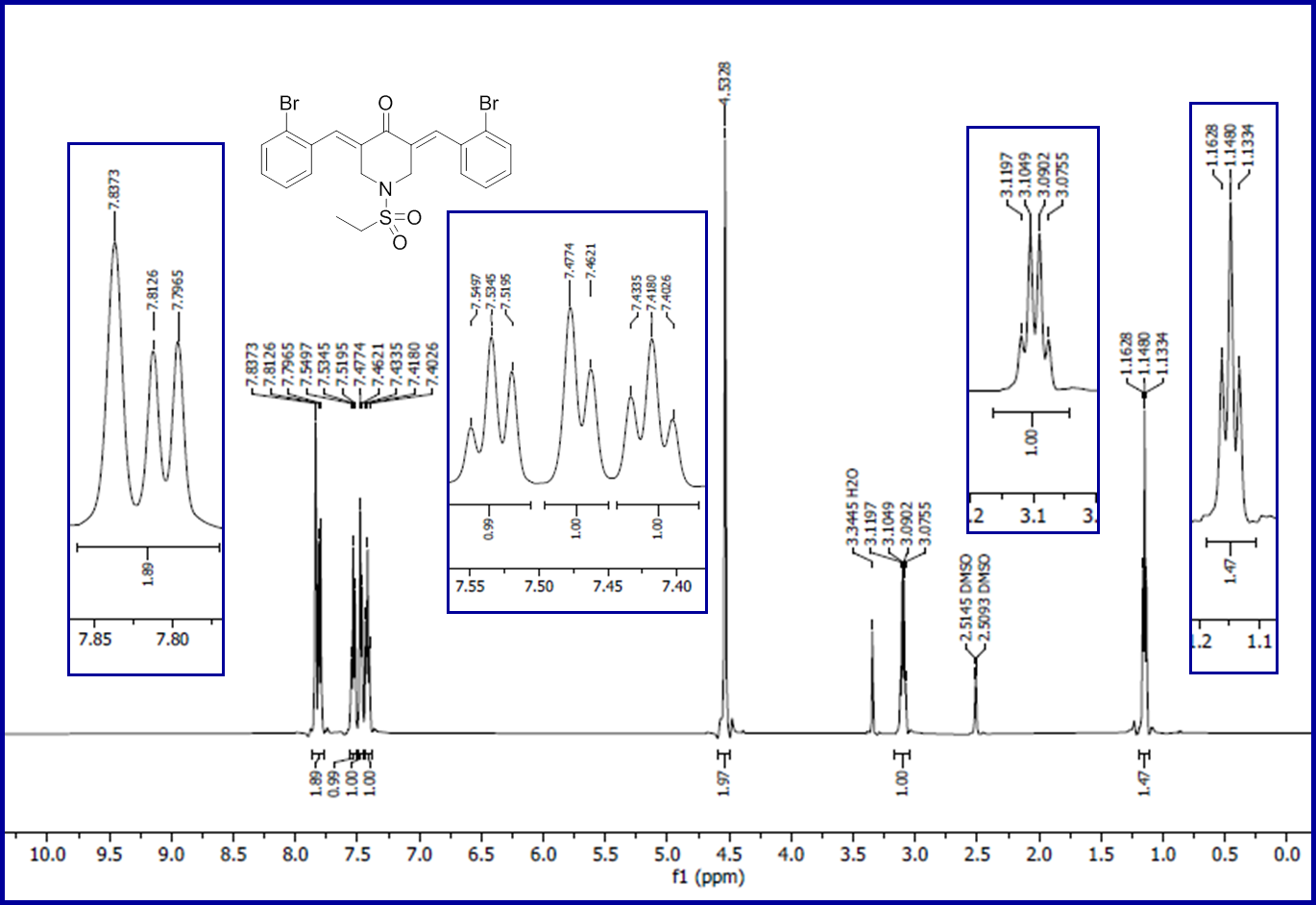


**Fig. S5.** ^1^H-NMR spectrum of compound **5x** in DMSO-*d_6_*.


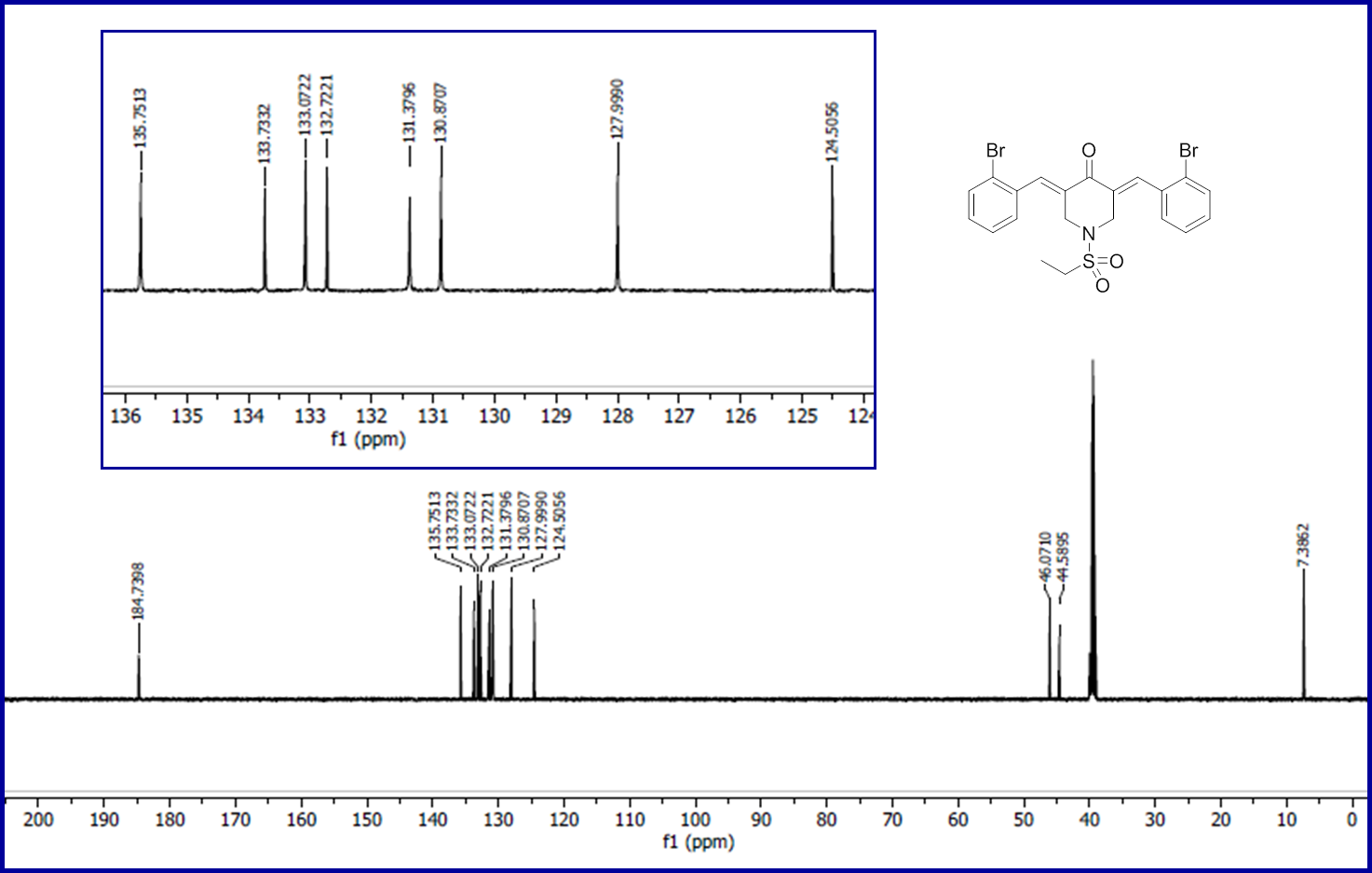


**Fig. S6.** ^13^C-NMR spectrum of compound **5x** in DMSO-*d_6_*.


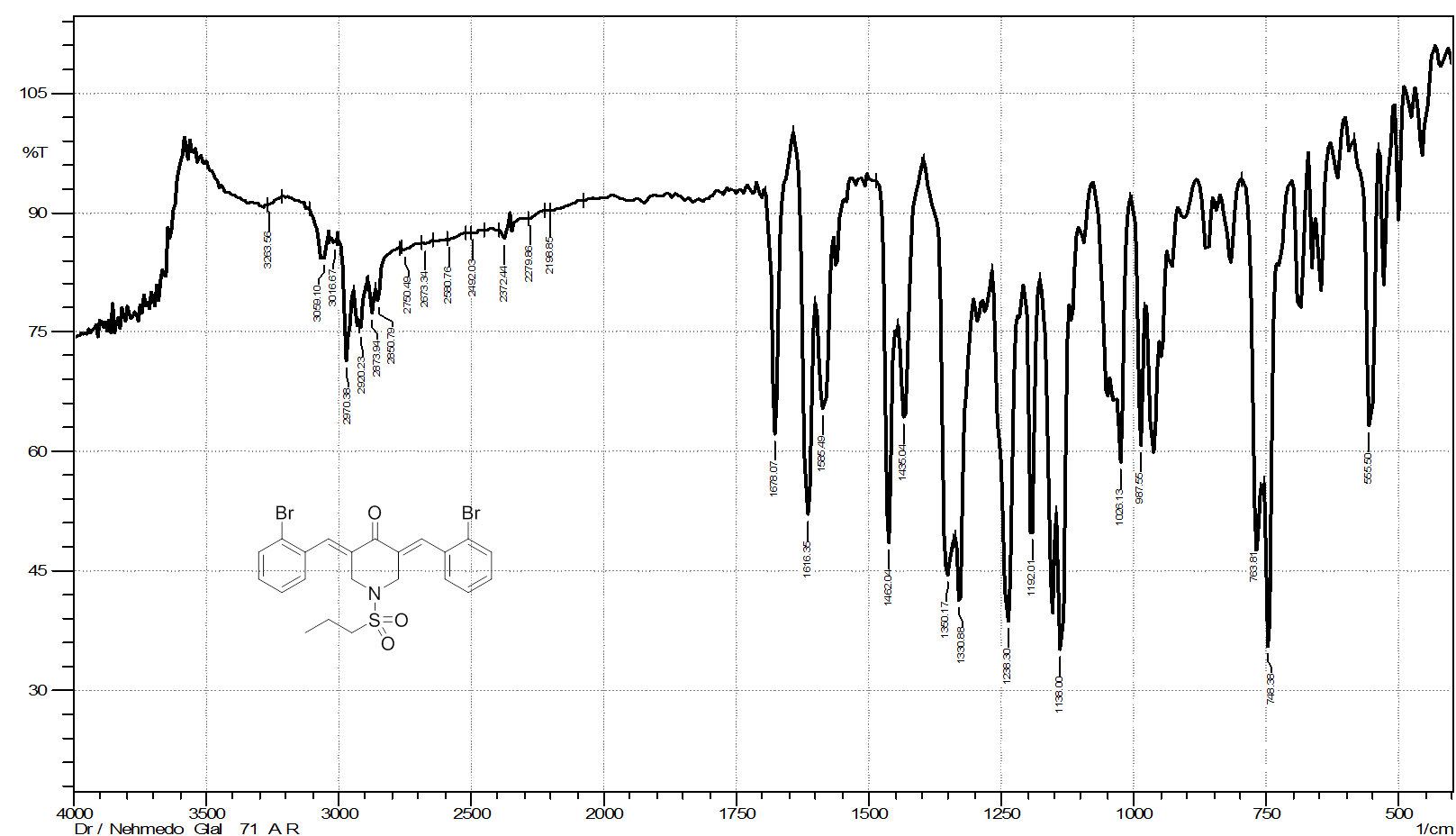


**Fig. S7.** IR spectrum of compound **5y** (KBr pellet).


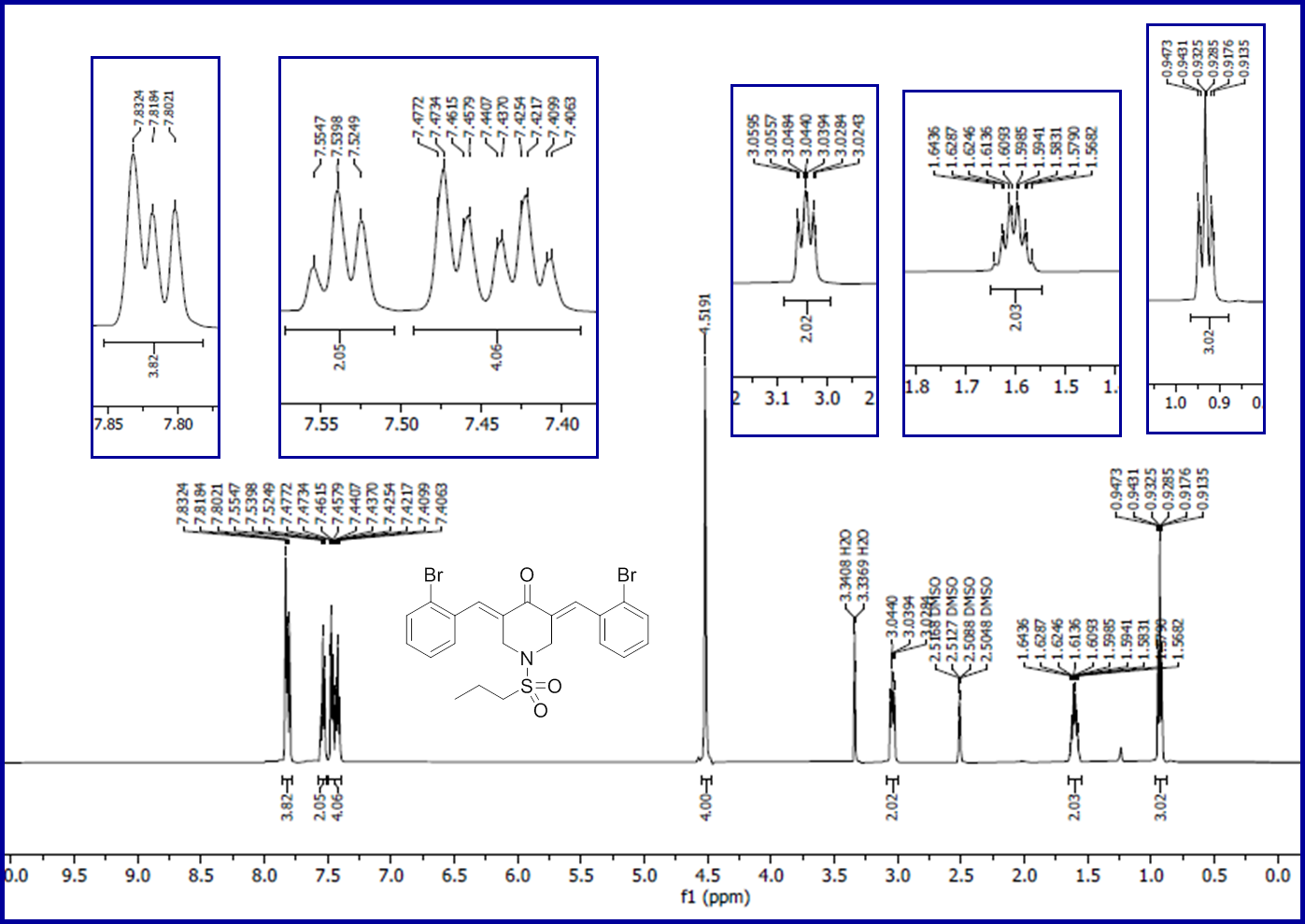


**Fig. S8.** ^1^H-NMR spectrum of compound **5y** in DMSO-*d_6_*.


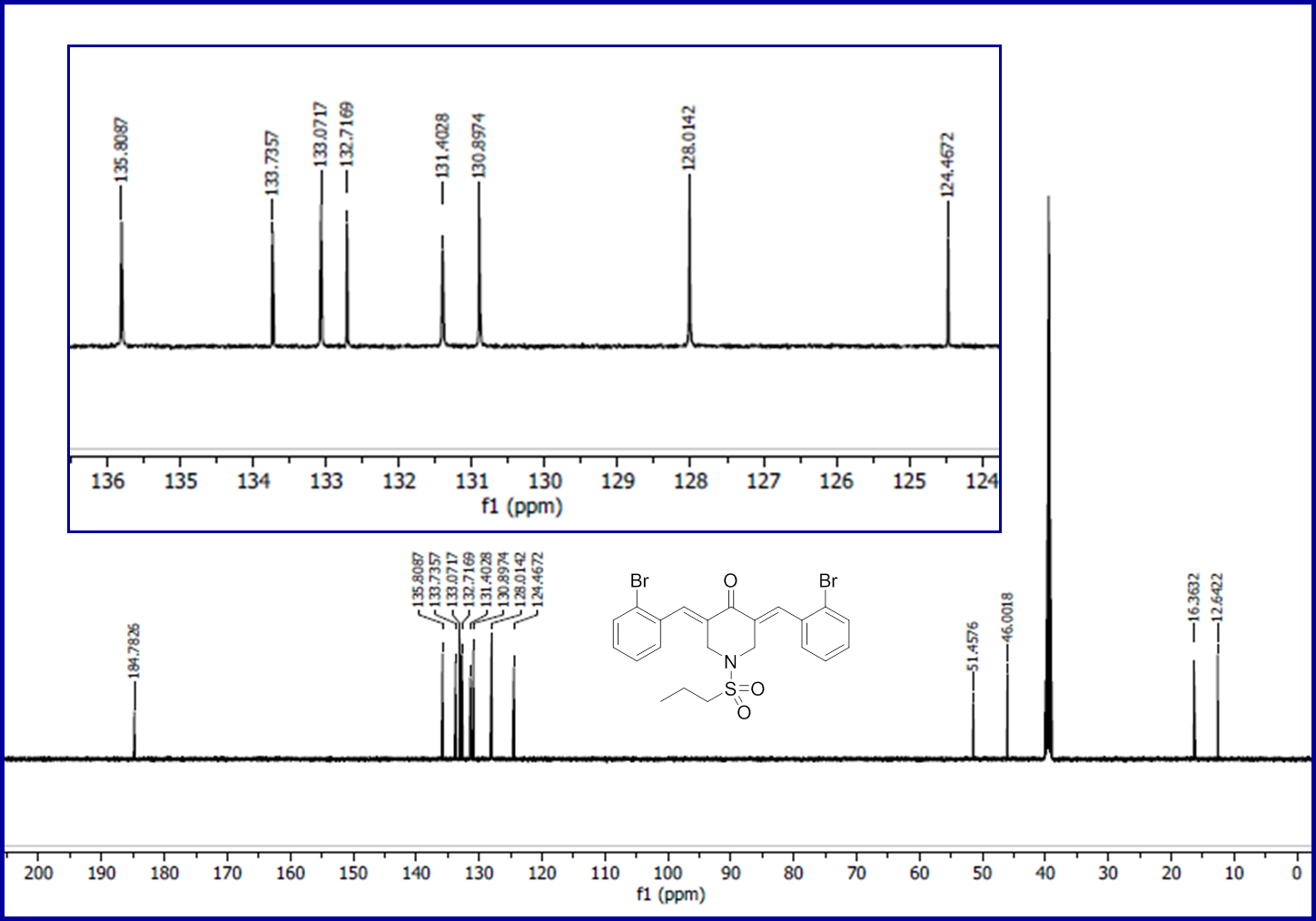


**Fig. S9.** ^13^C-NMR spectrum of compound **5y** in DMSO-*d_6_*.


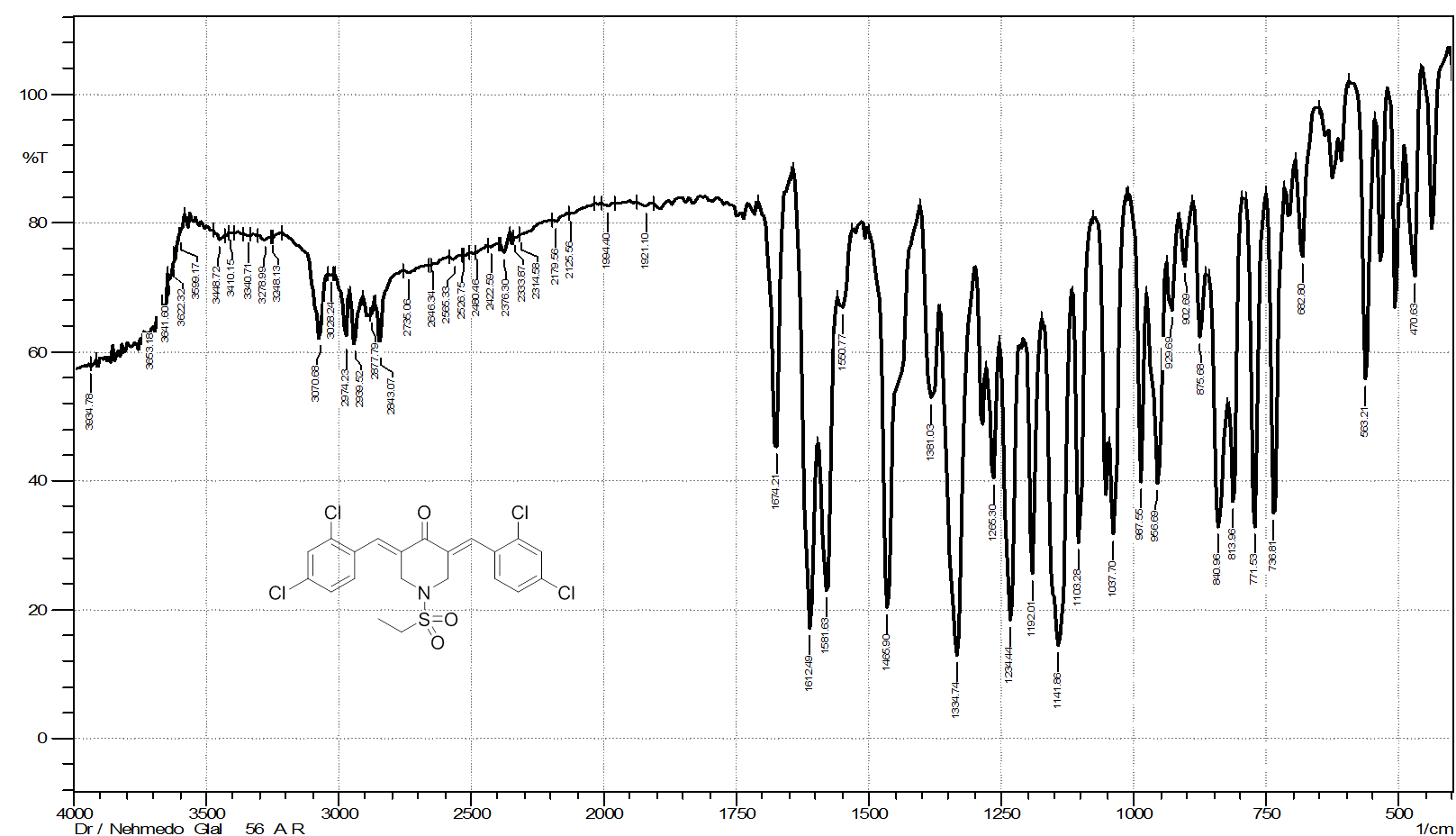


**Fig. S10.** IR spectrum of compound **5z** (KBr pellet).


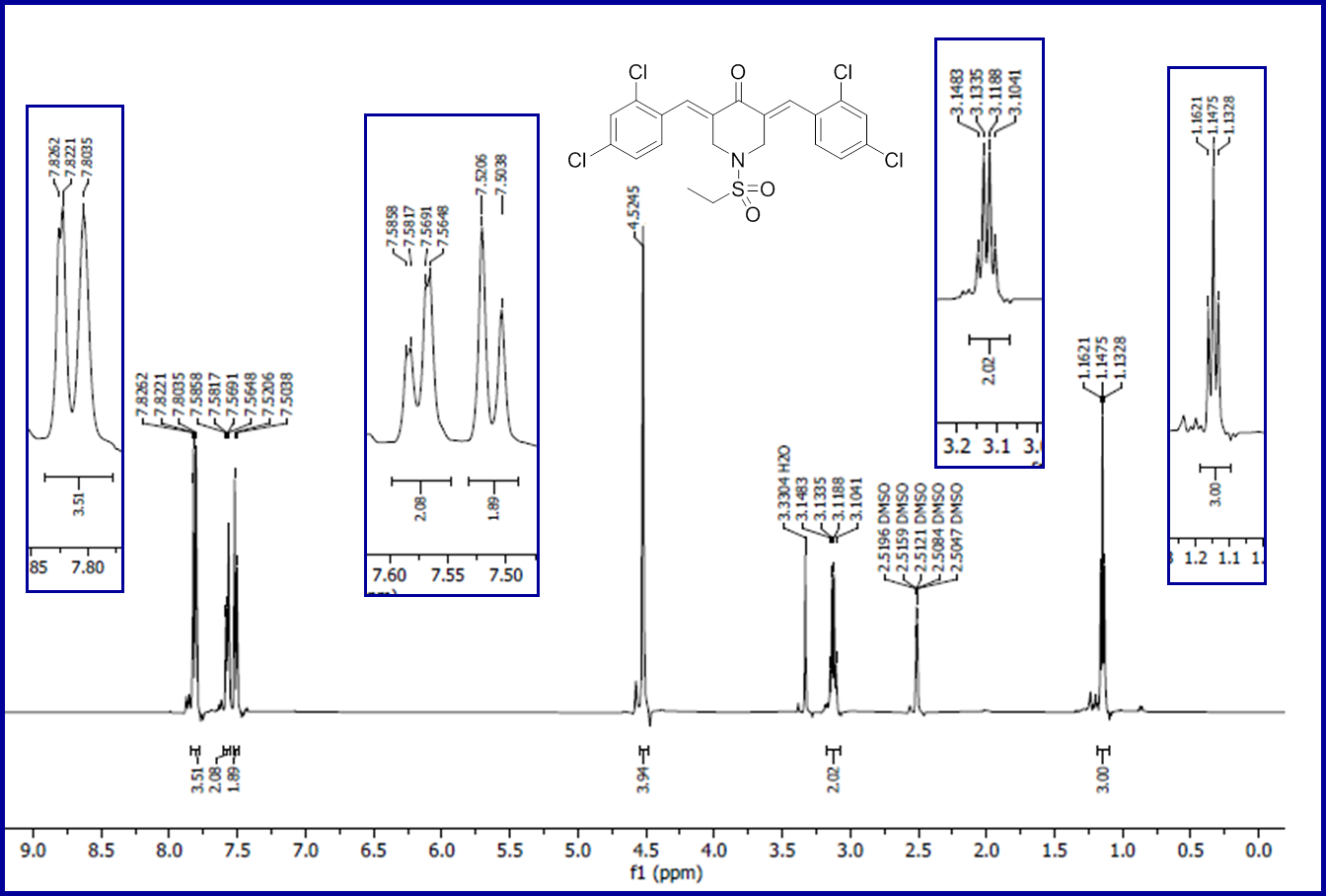


**Fig. S11.** ^1^H-NMR spectrum of compound **5z** in DMSO-*d_6_*.


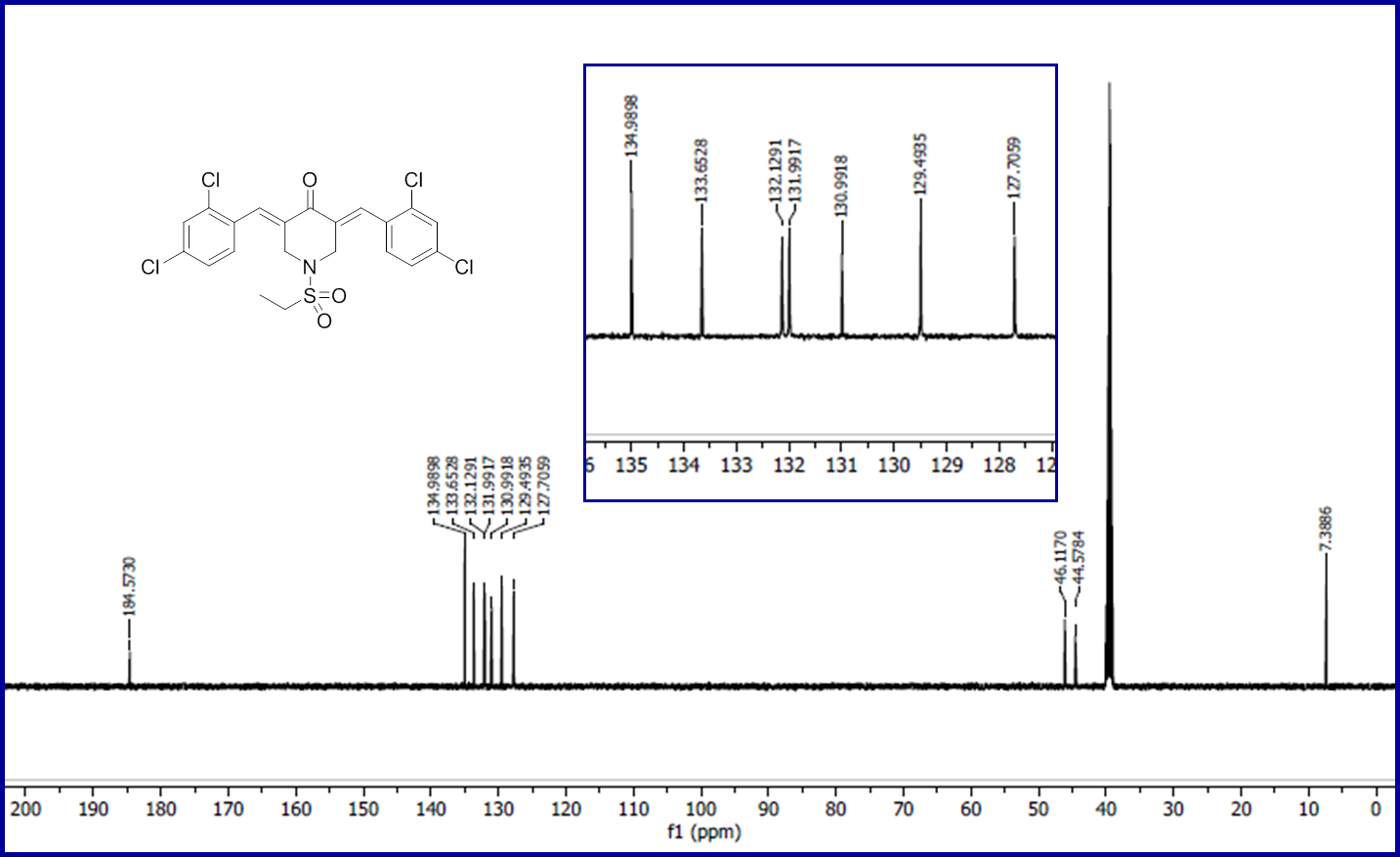


**Fig. S12.** ^13^C-NMR spectrum of compound **5z** in DMSO-*d_6_*.


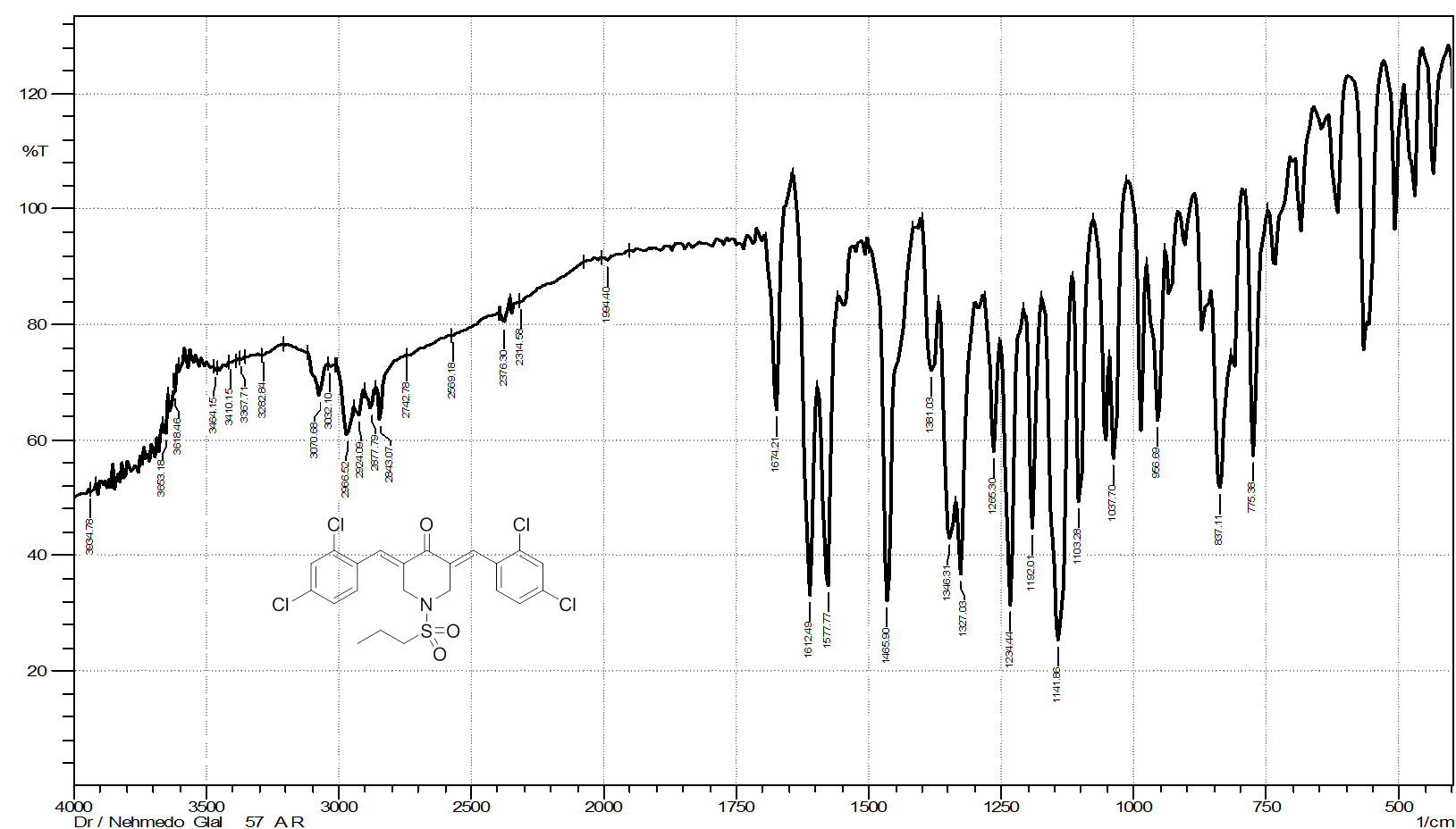


**Fig. S13.** IR spectrum of compound **5aa** (KBr pellet).


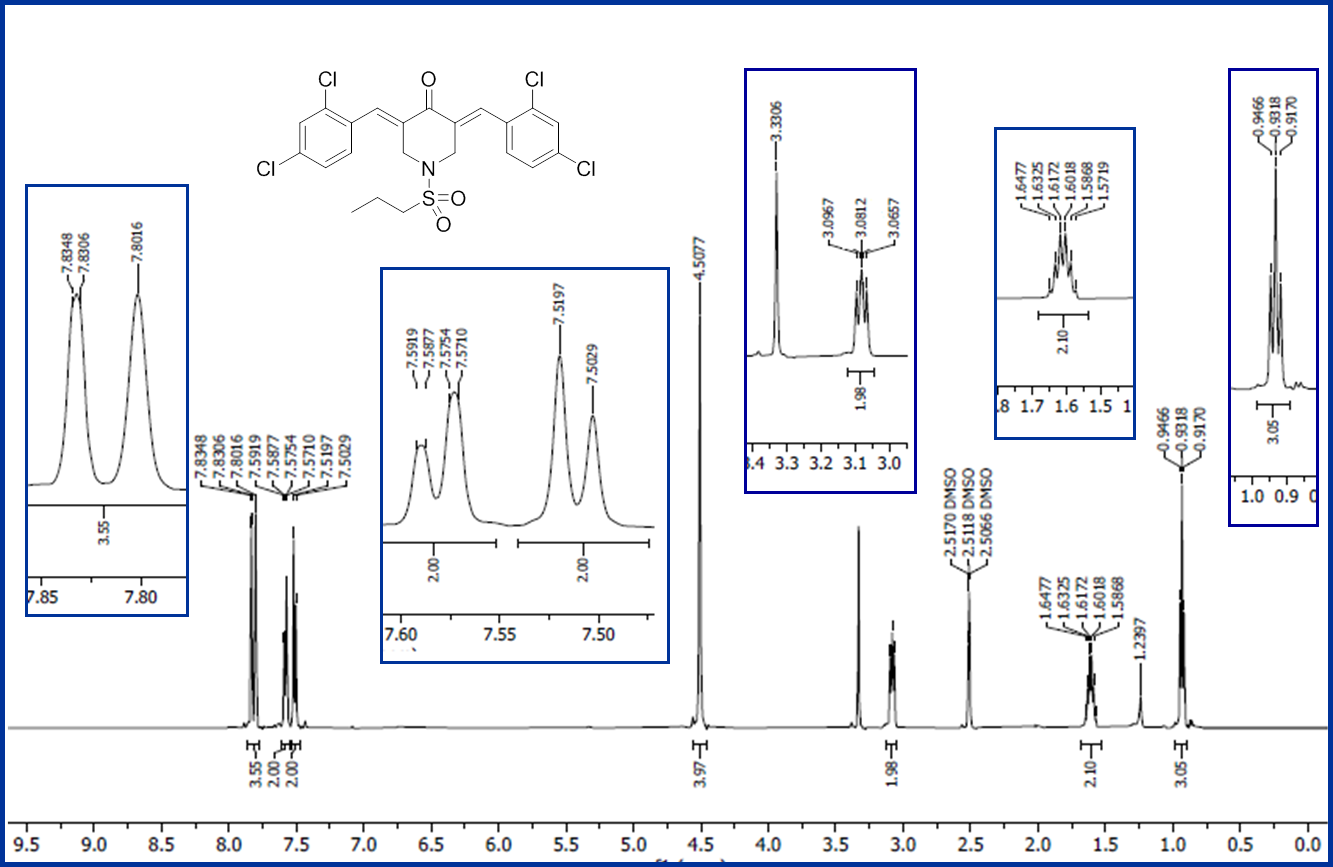


**Fig. S14.** ^1^H-NMR spectrum of compound **5aa** in DMSO-*d_6_*.


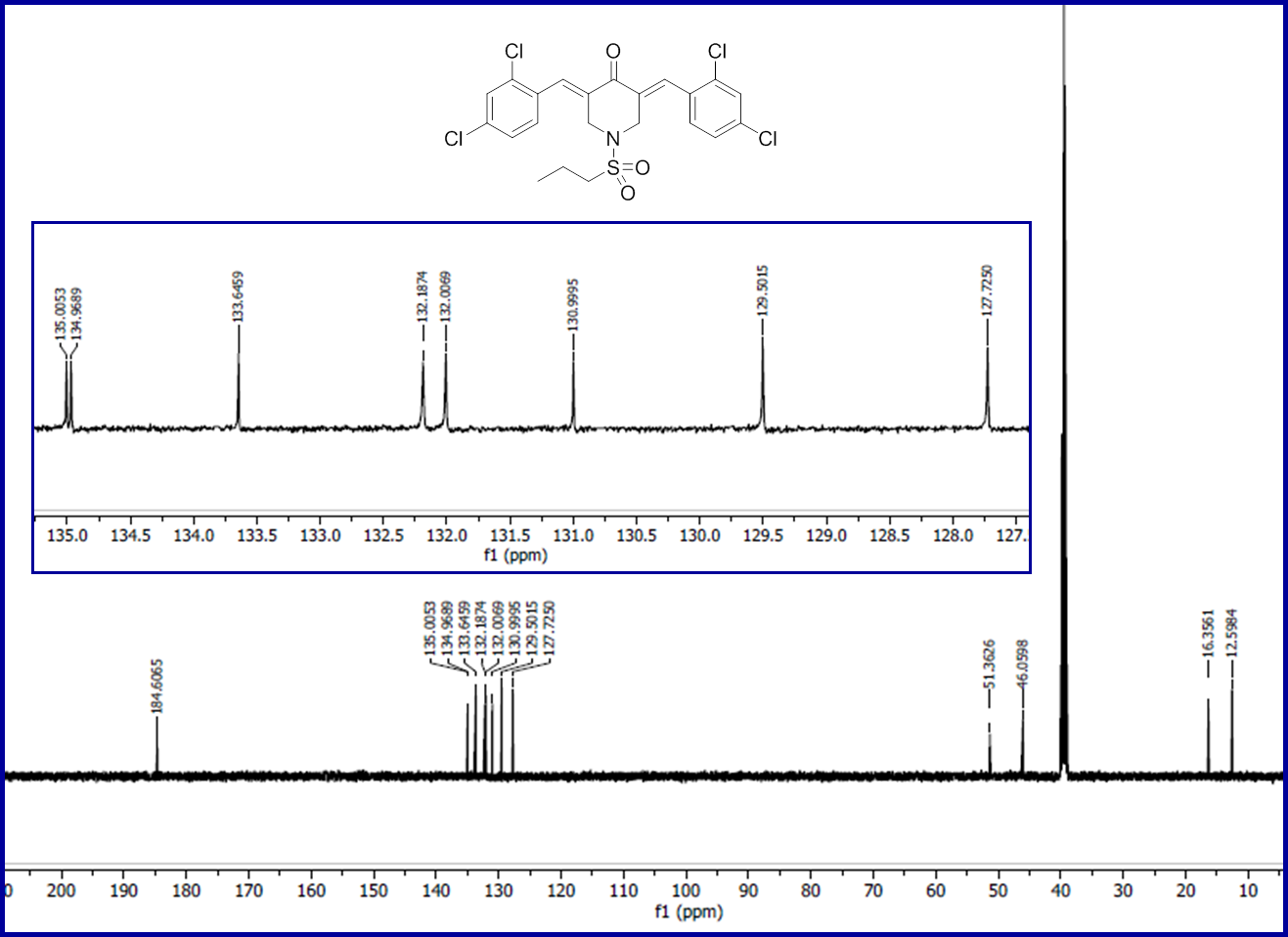


**Fig. S15.** ^13^C-NMR spectrum of compound **5aa** in DMSO-*d_6_*.


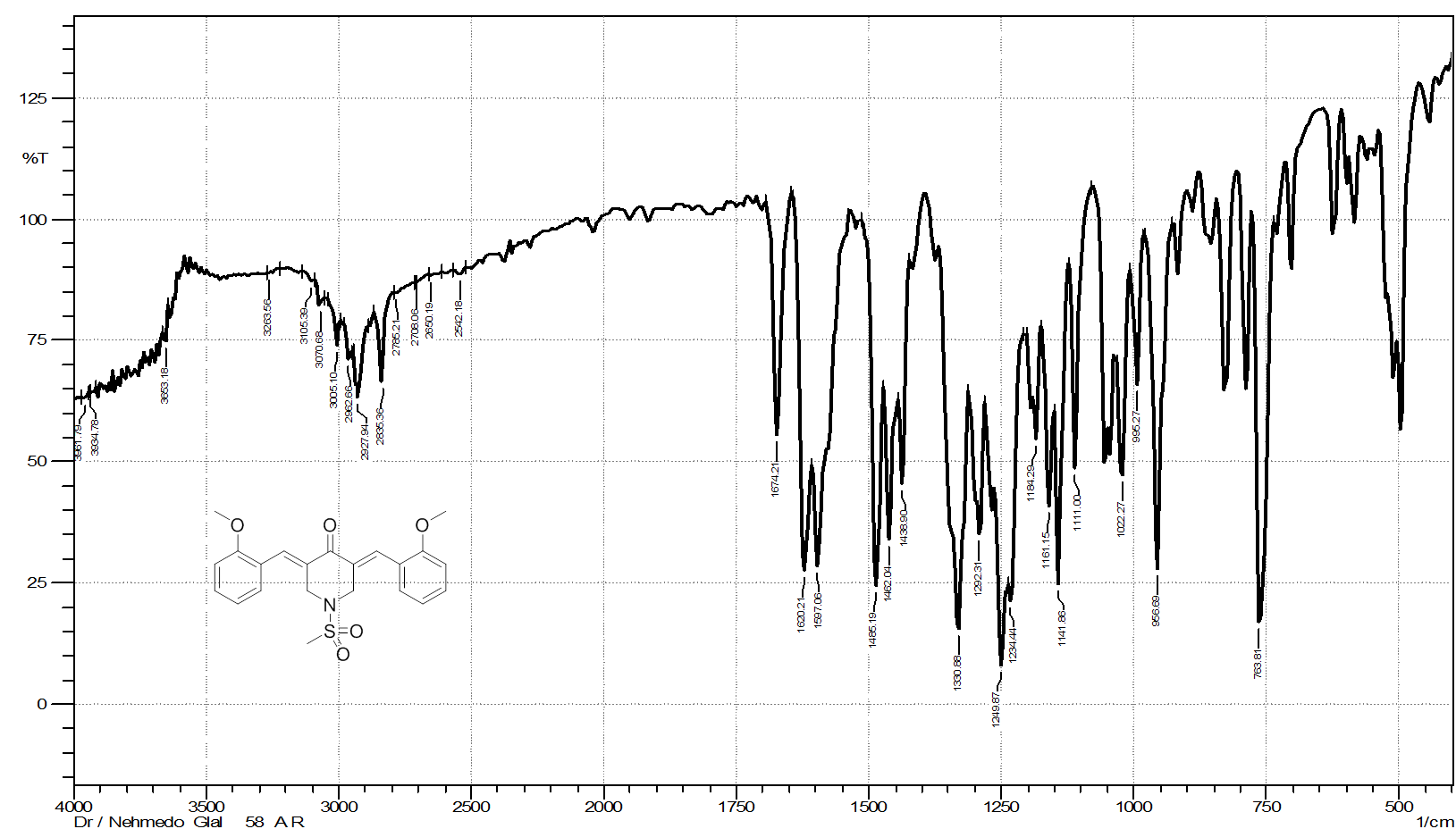


**Fig. S16.** IR spectrum of compound **5ab** (KBr pellet).


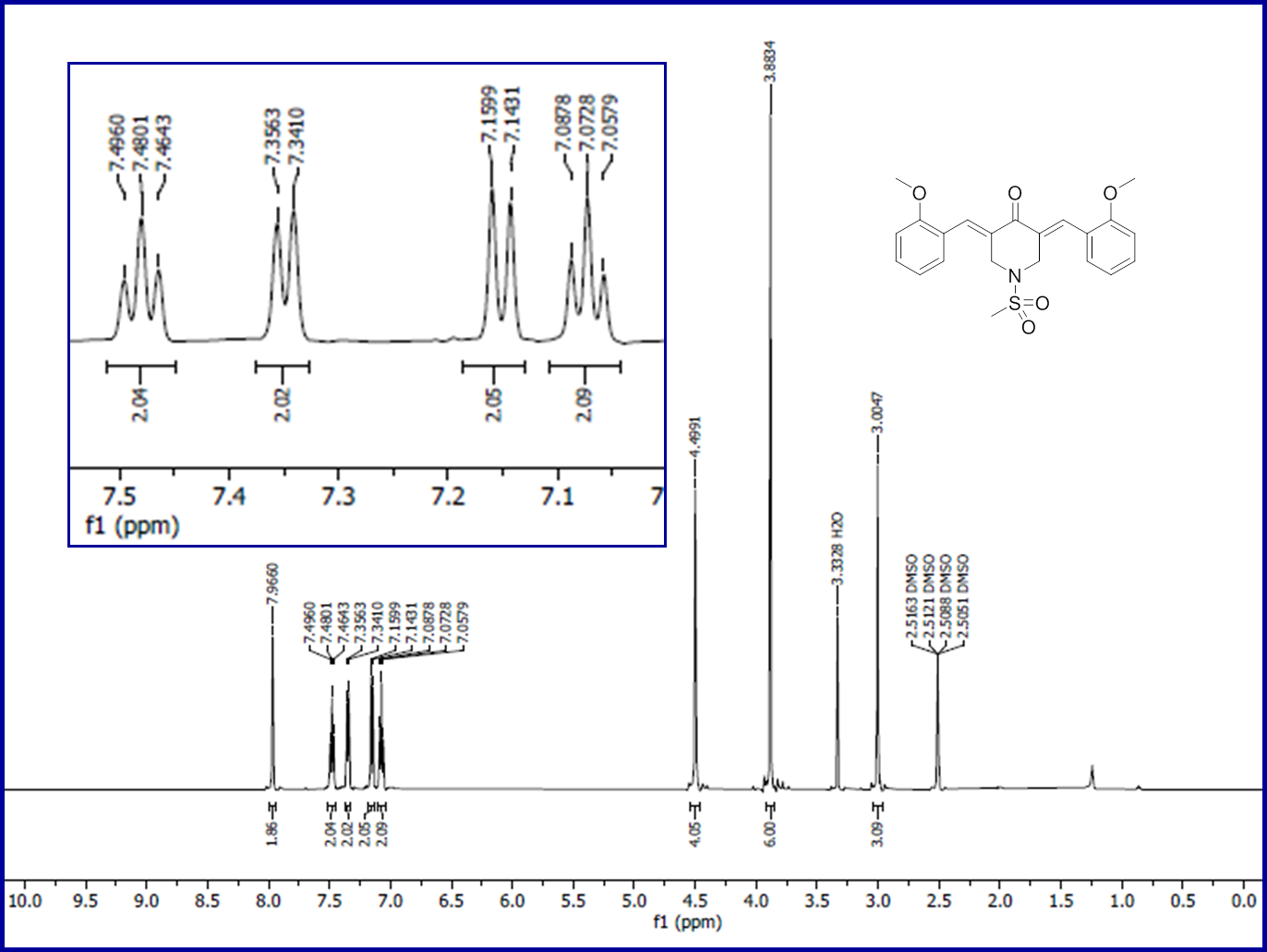


**Fig. S17.** ^1^H-NMR spectrum of compound **5ab** in DMSO-*d_6_*.


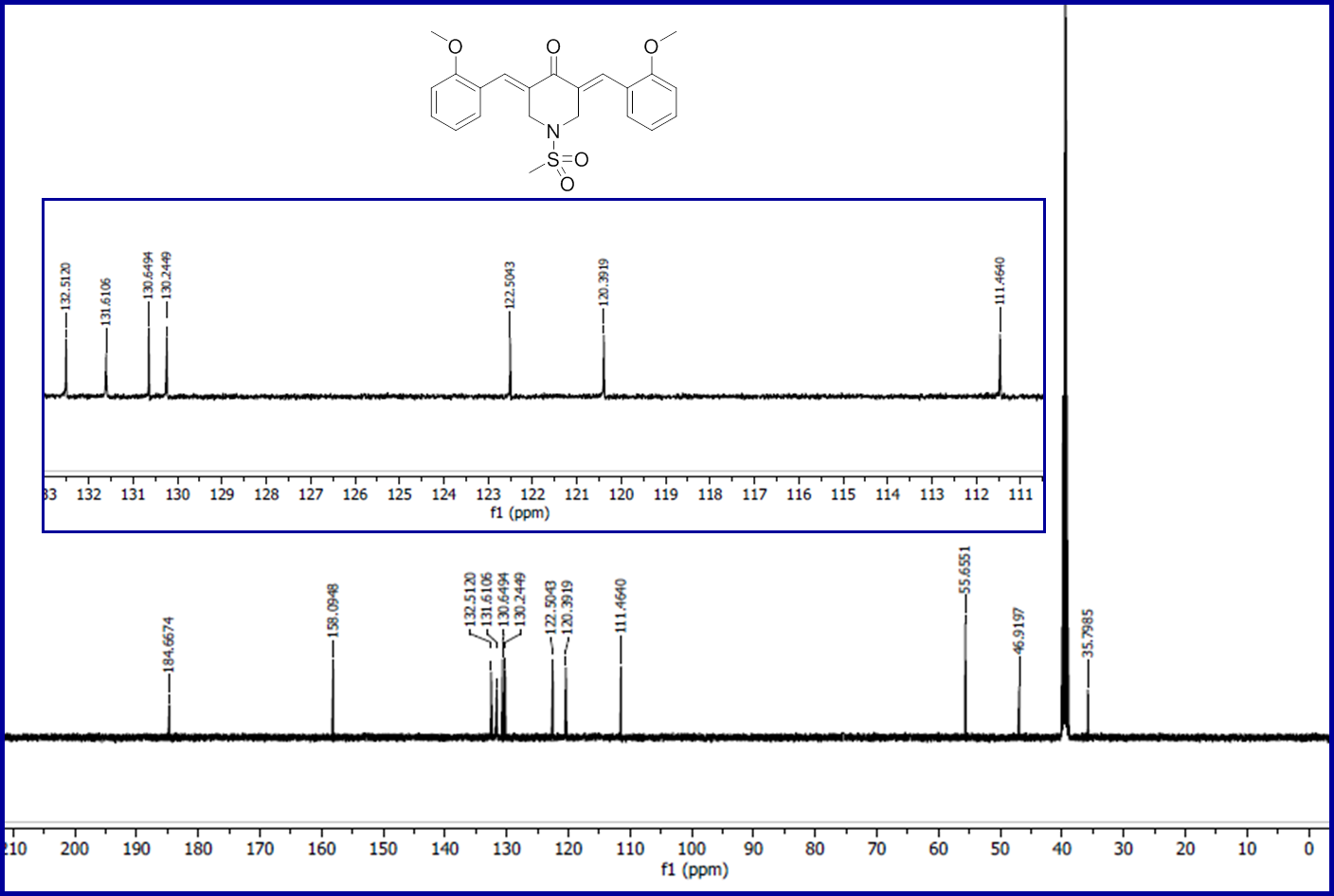


**Fig. S18.** ^13^C-NMR spectrum of compound **5ab** in DMSO-*d_6_*.


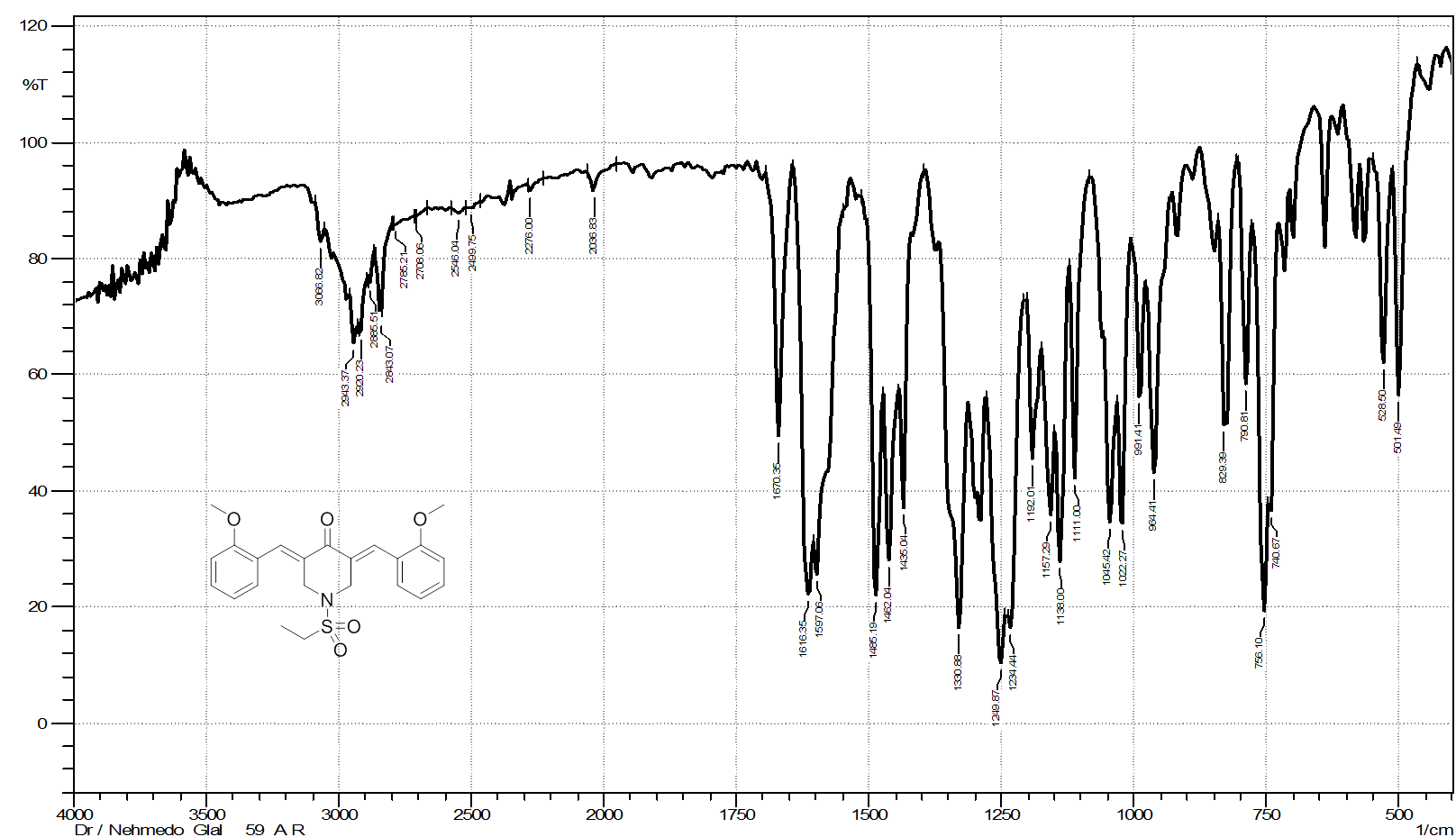


**Fig. S19.** IR spectrum of compound **5ac** (KBr pellet).


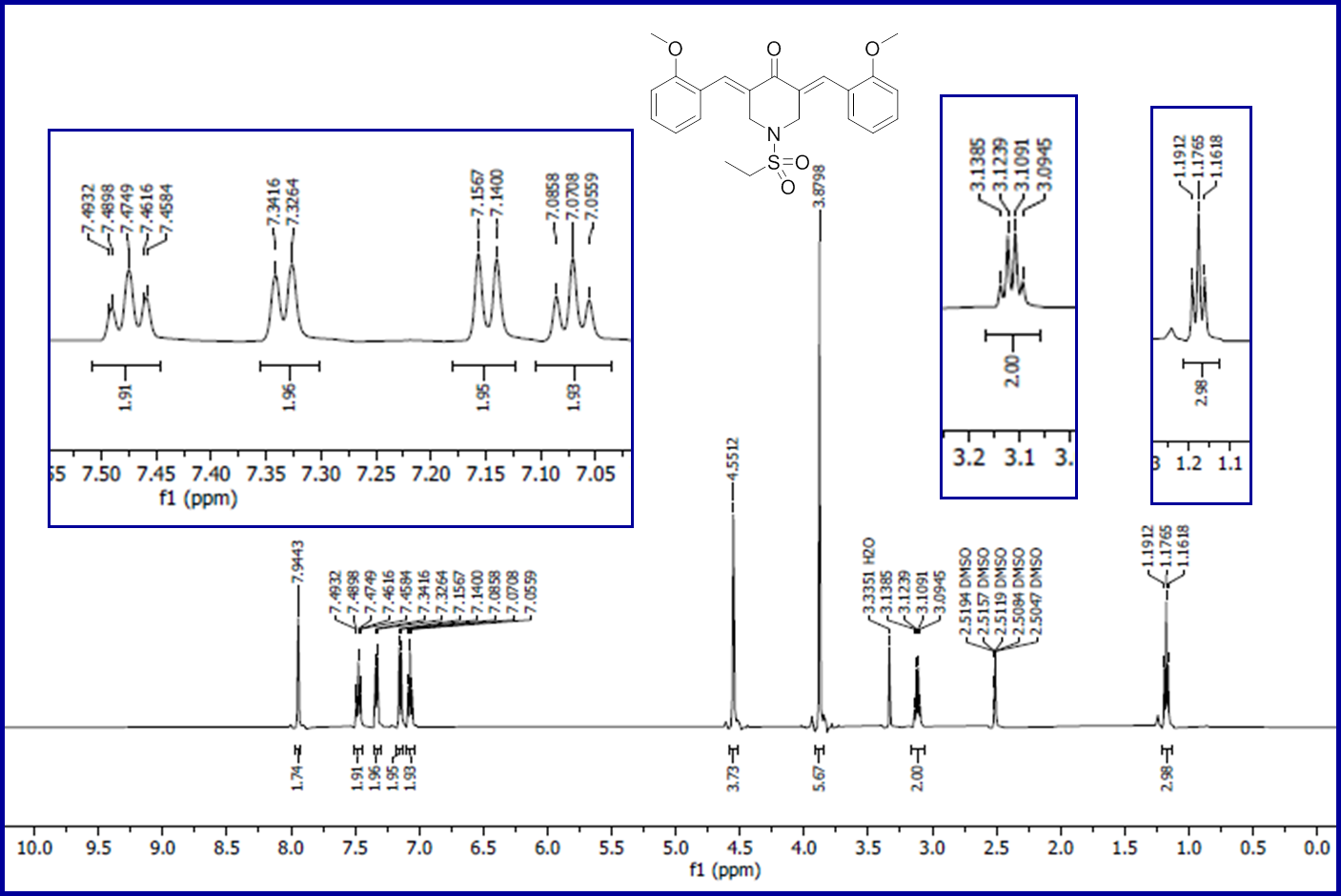


**Fig. S20.** ^1^H-NMR spectrum of compound **5ac** in DMSO-*d_6_*.


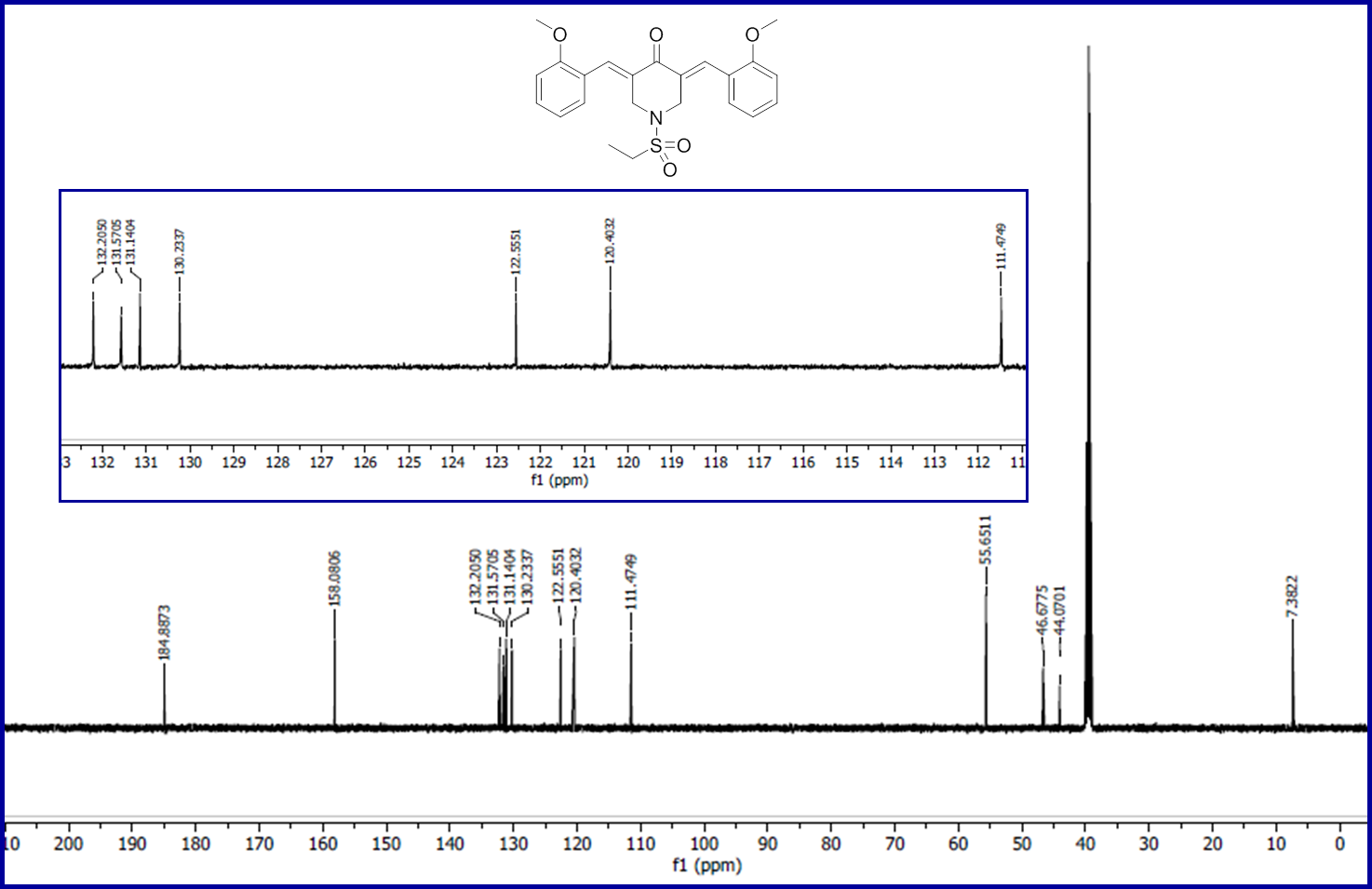


**Fig. S21.** ^13^C-NMR spectrum of compound **5ac** in DMSO-*d_6_*.


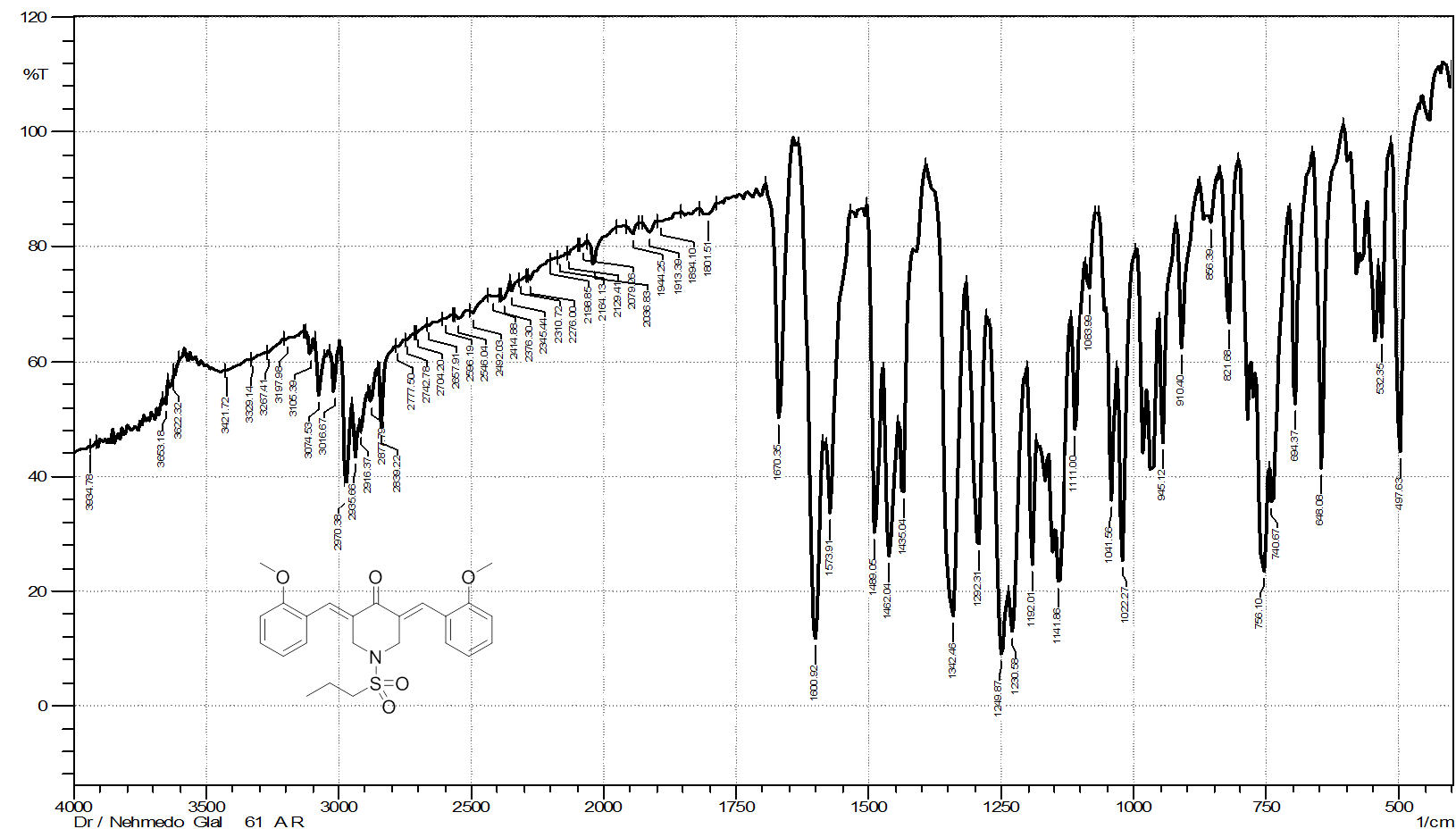


**Fig. S22.** IR spectrum of compound **5ad** (KBr pellet).


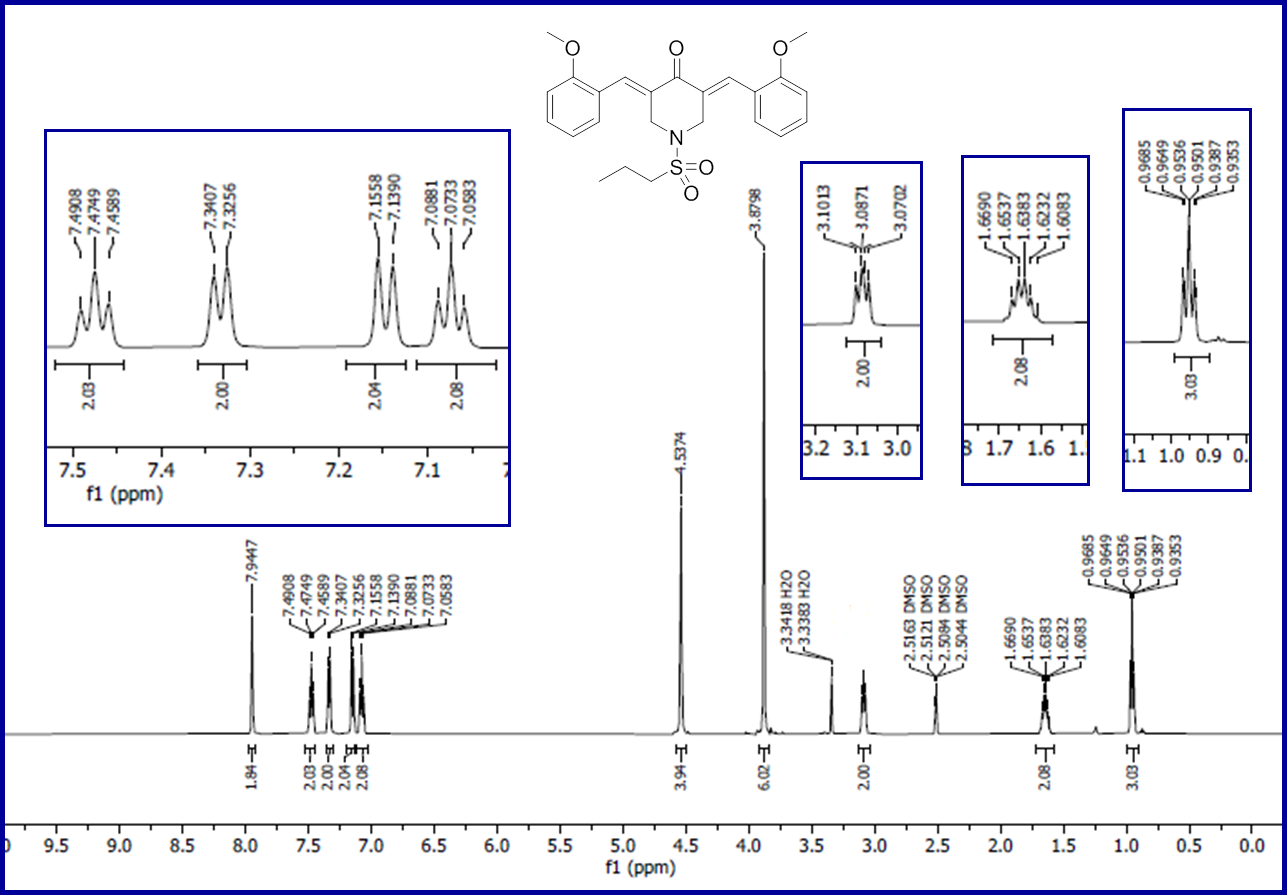


**Fig. S23.** ^1^H-NMR spectrum of compound **5ad** in DMSO-*d_6_*.


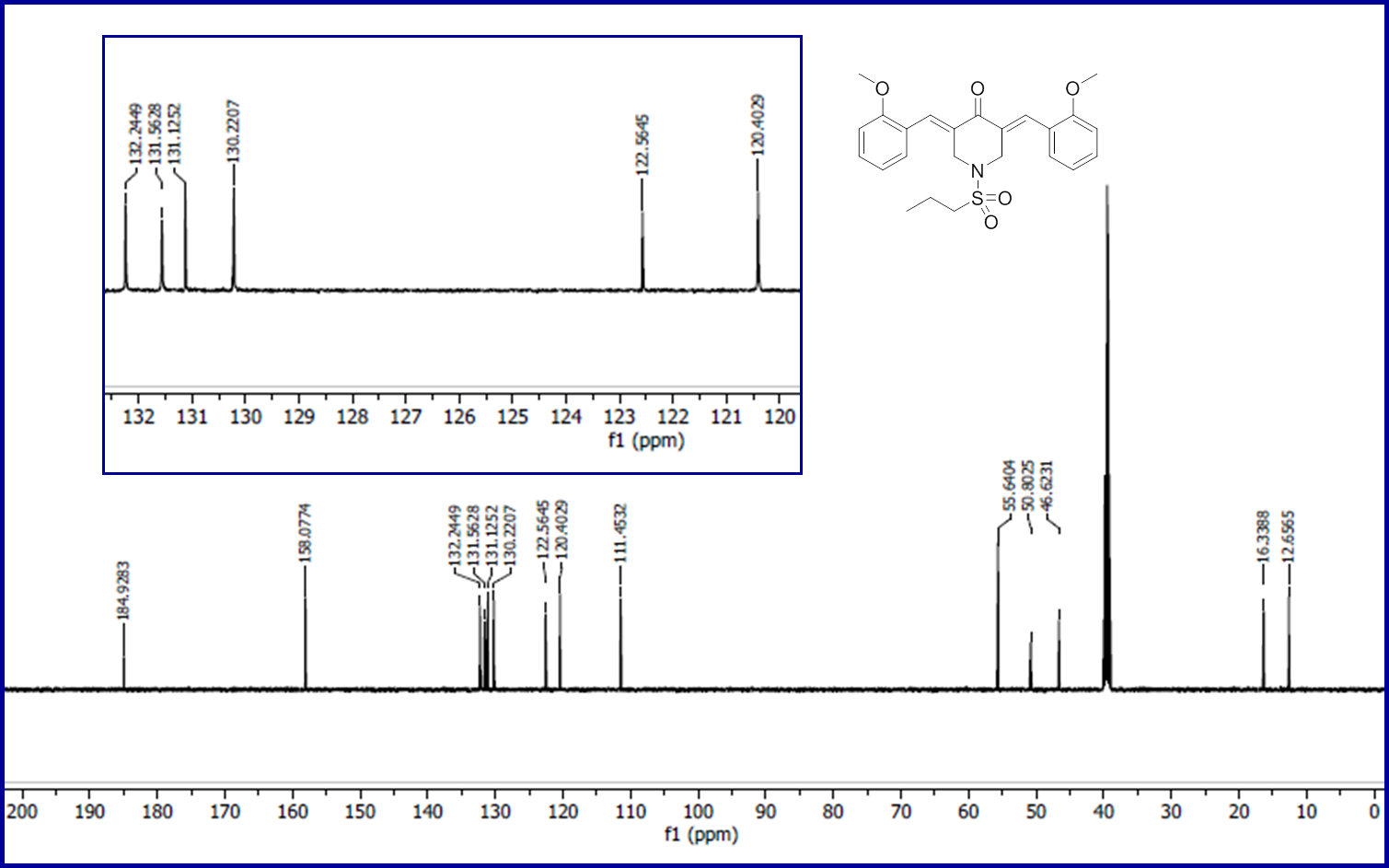


**Fig. S24.** ^13^C-NMR spectrum of compound **5ad** in DMSO-*d_6_*.


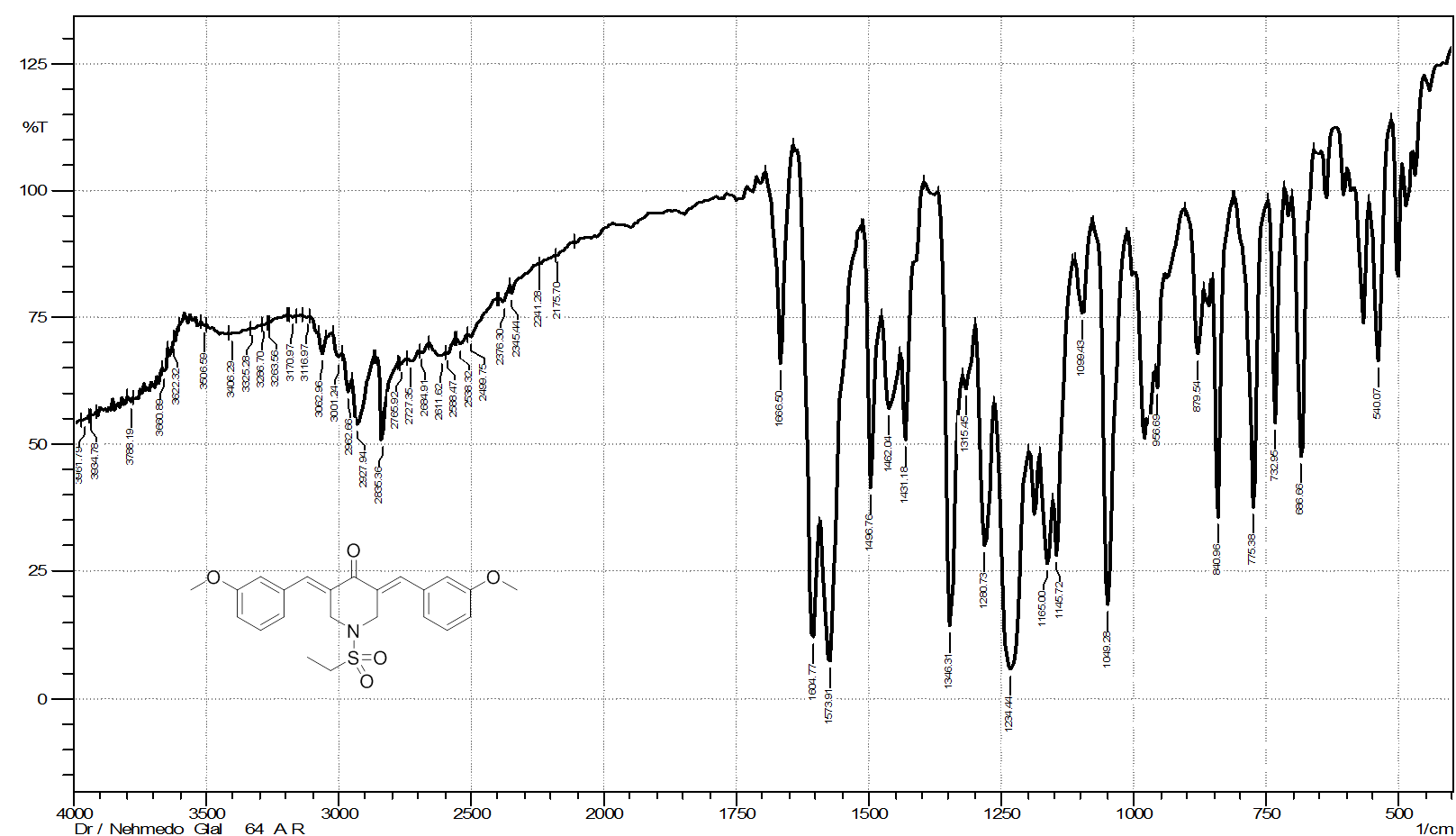


**Fig. S25.** IR spectrum of compound **5ae** (KBr pellet).


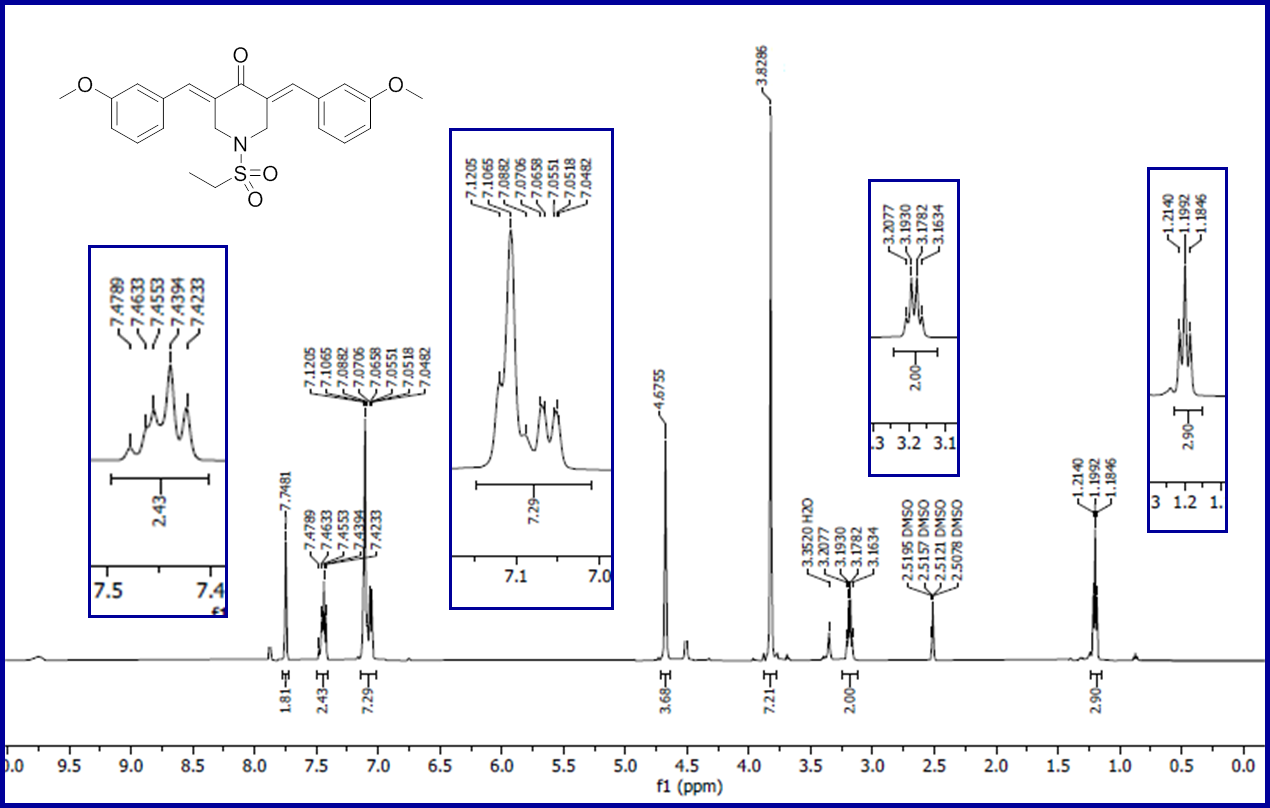


**Fig. S26.** ^1^H-NMR spectrum of compound **5ae** in DMSO-*d_6_*.


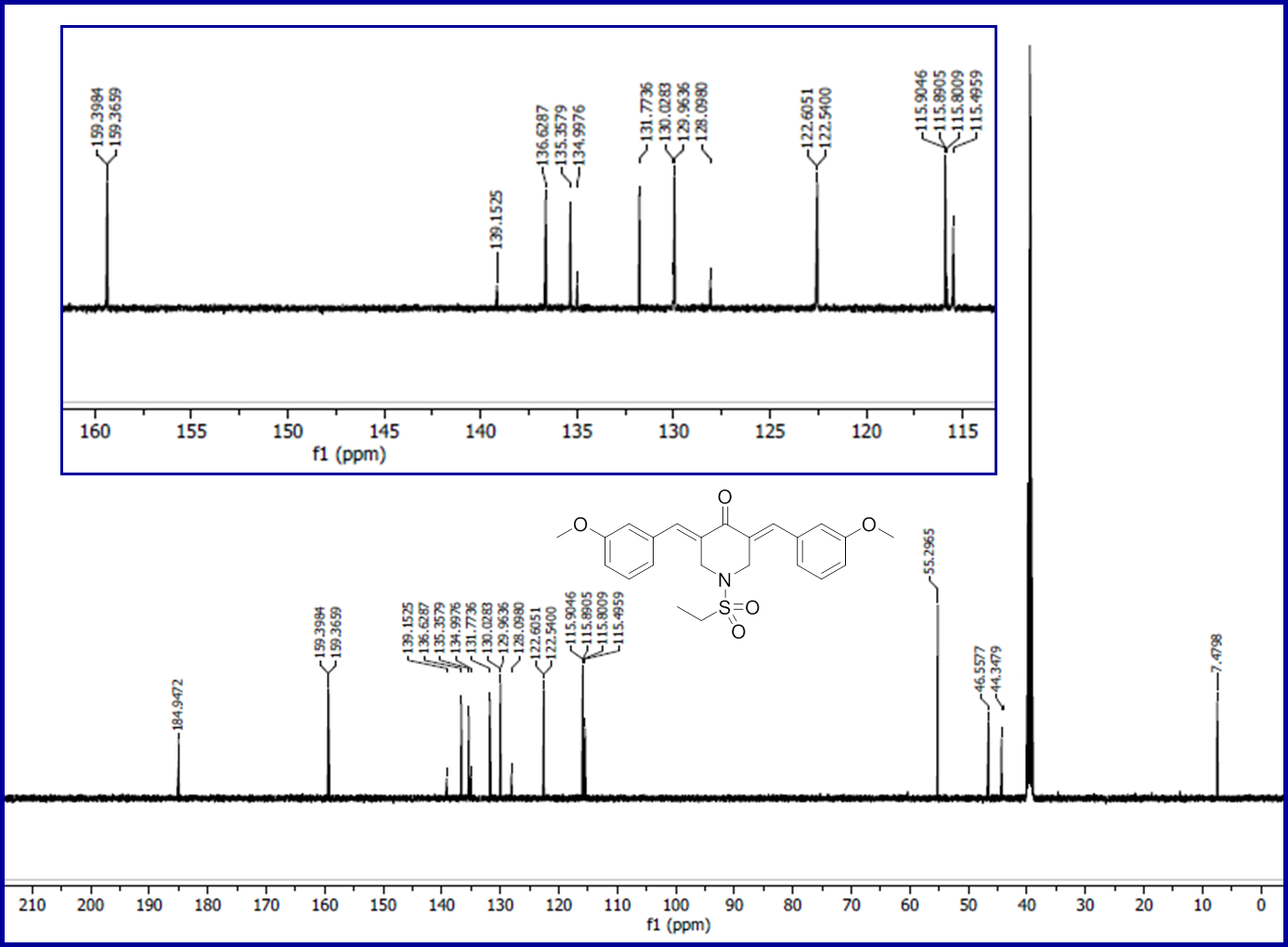


**Fig. S27.** ^13^C-NMR spectrum of compound **5ae** in DMSO-*d_6_*.


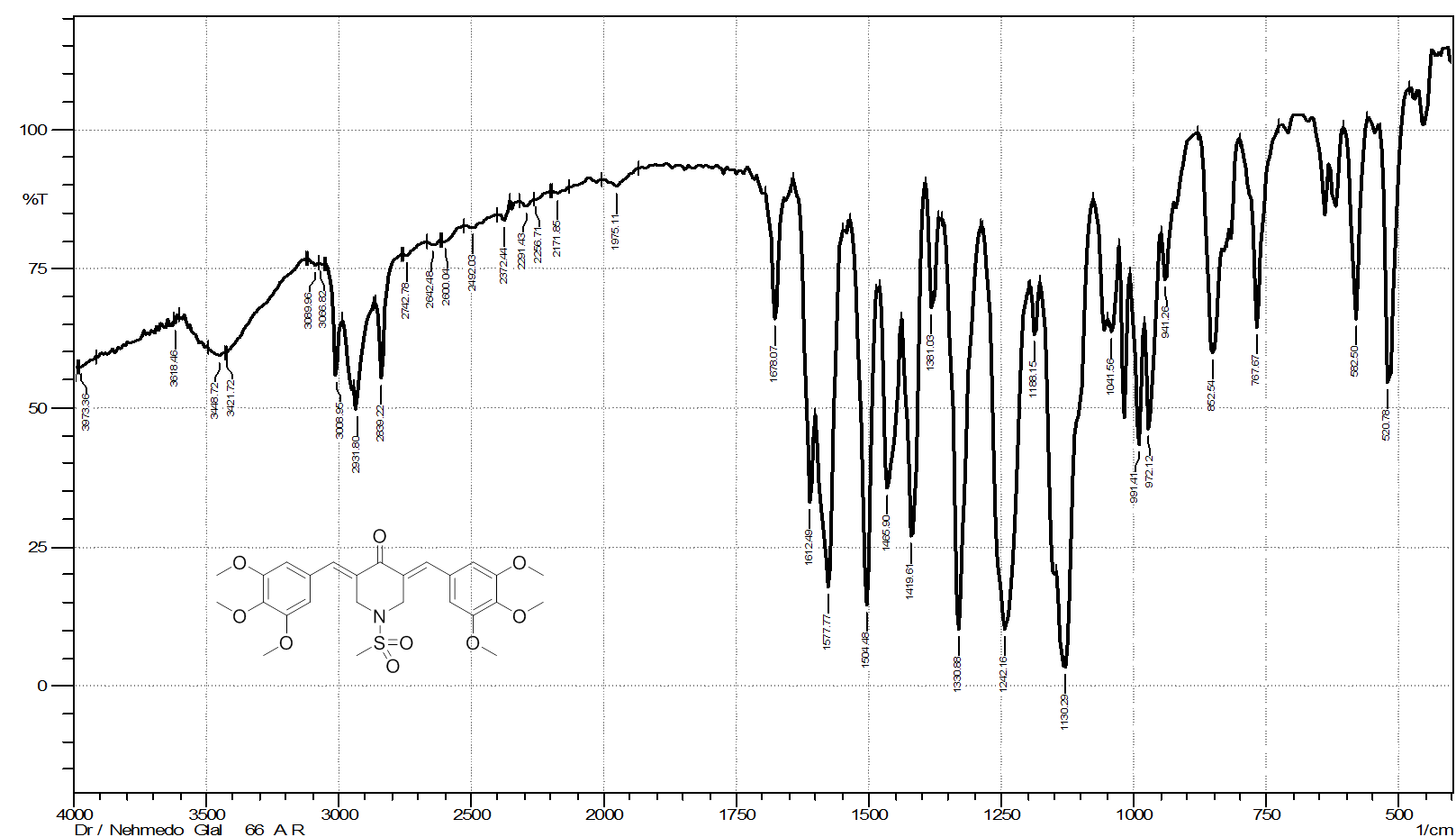


**Fig. S28.** IR spectrum of compound **5af** (KBr pellet).


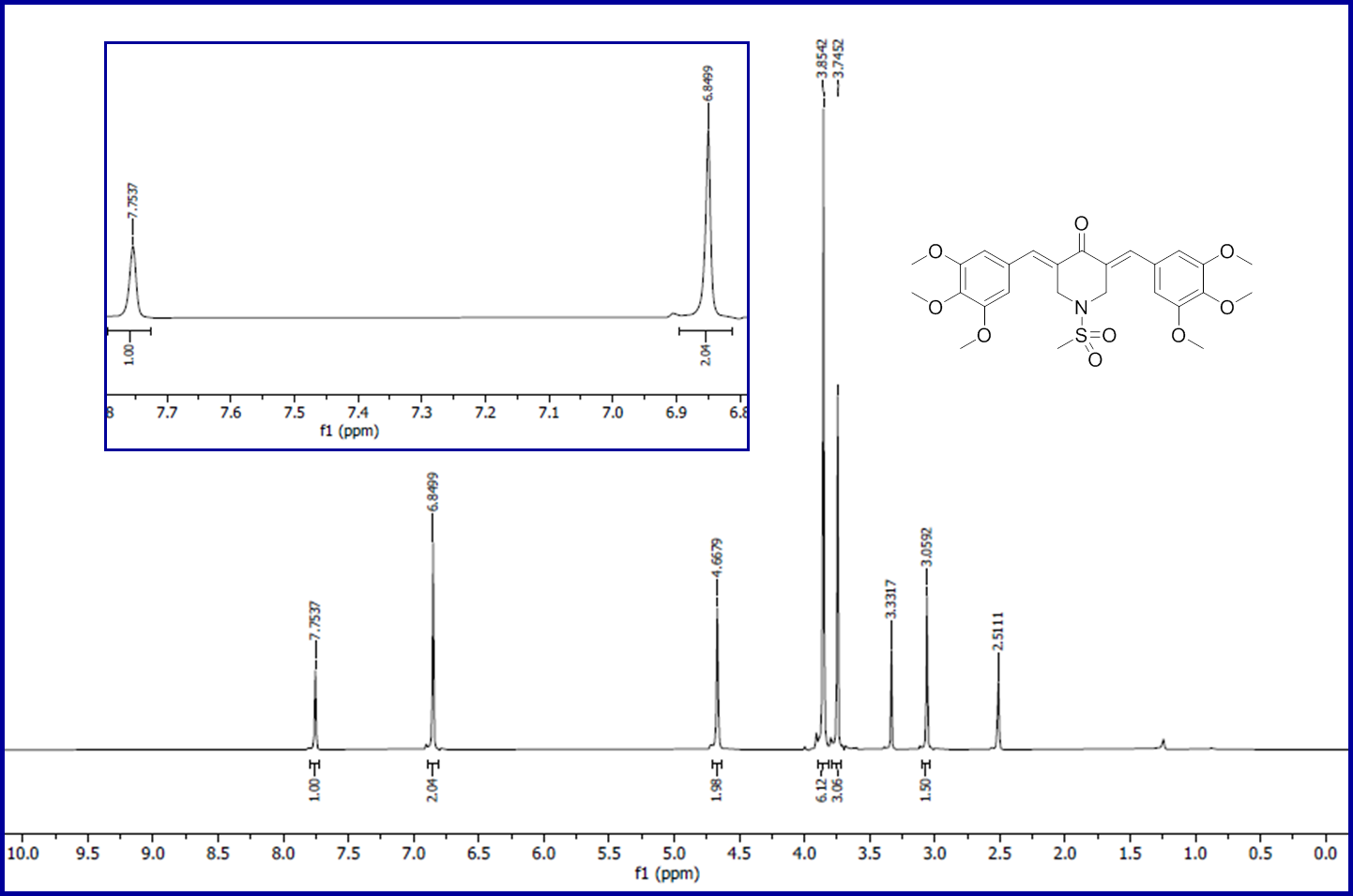


**Fig. S29.** ^1^H-NMR spectrum of compound **5af** in DMSO-*d_6_*.


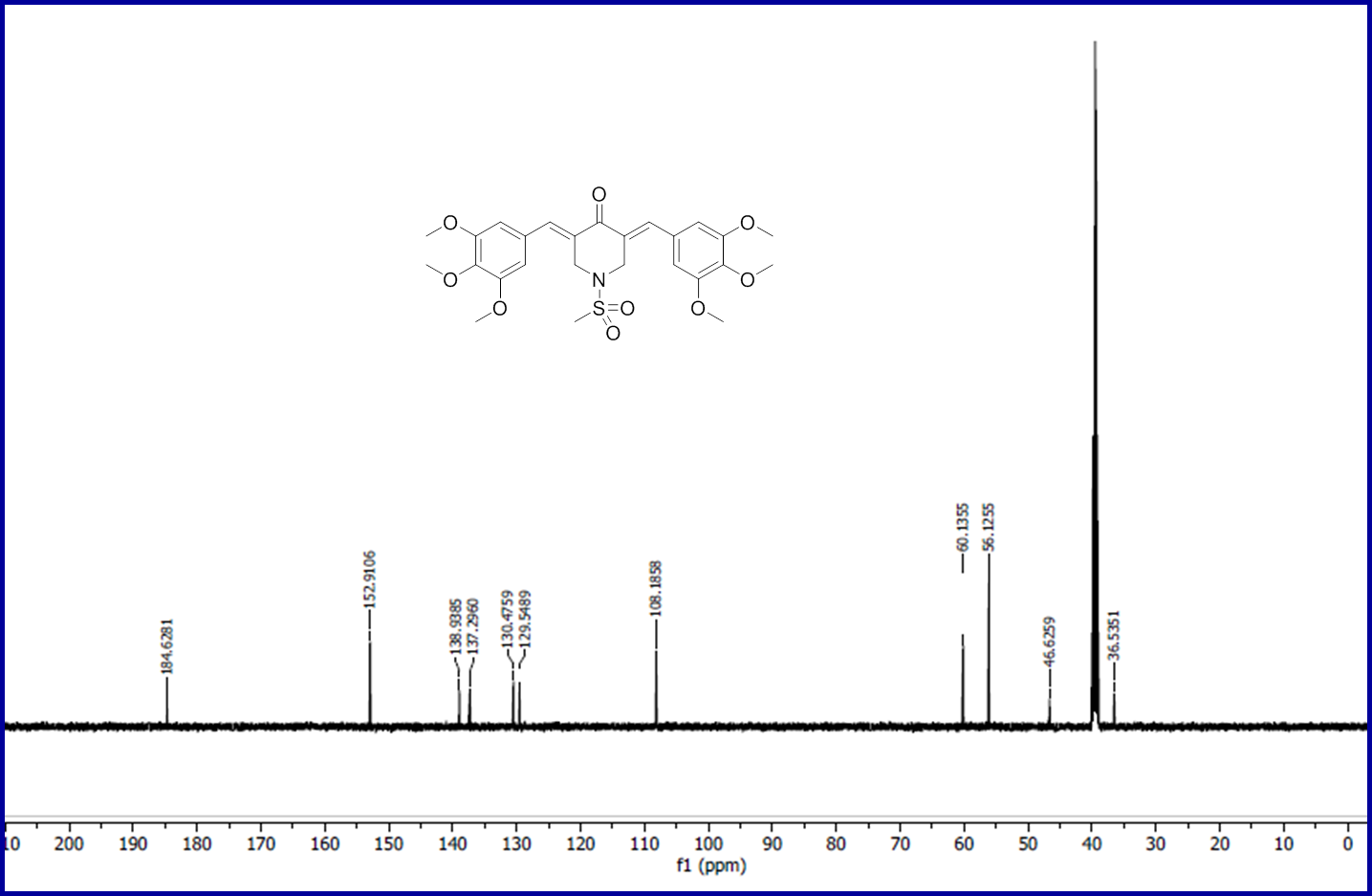


**Fig. S30.** ^13^C-NMR spectrum of compound **5af** in DMSO-*d_6_*.


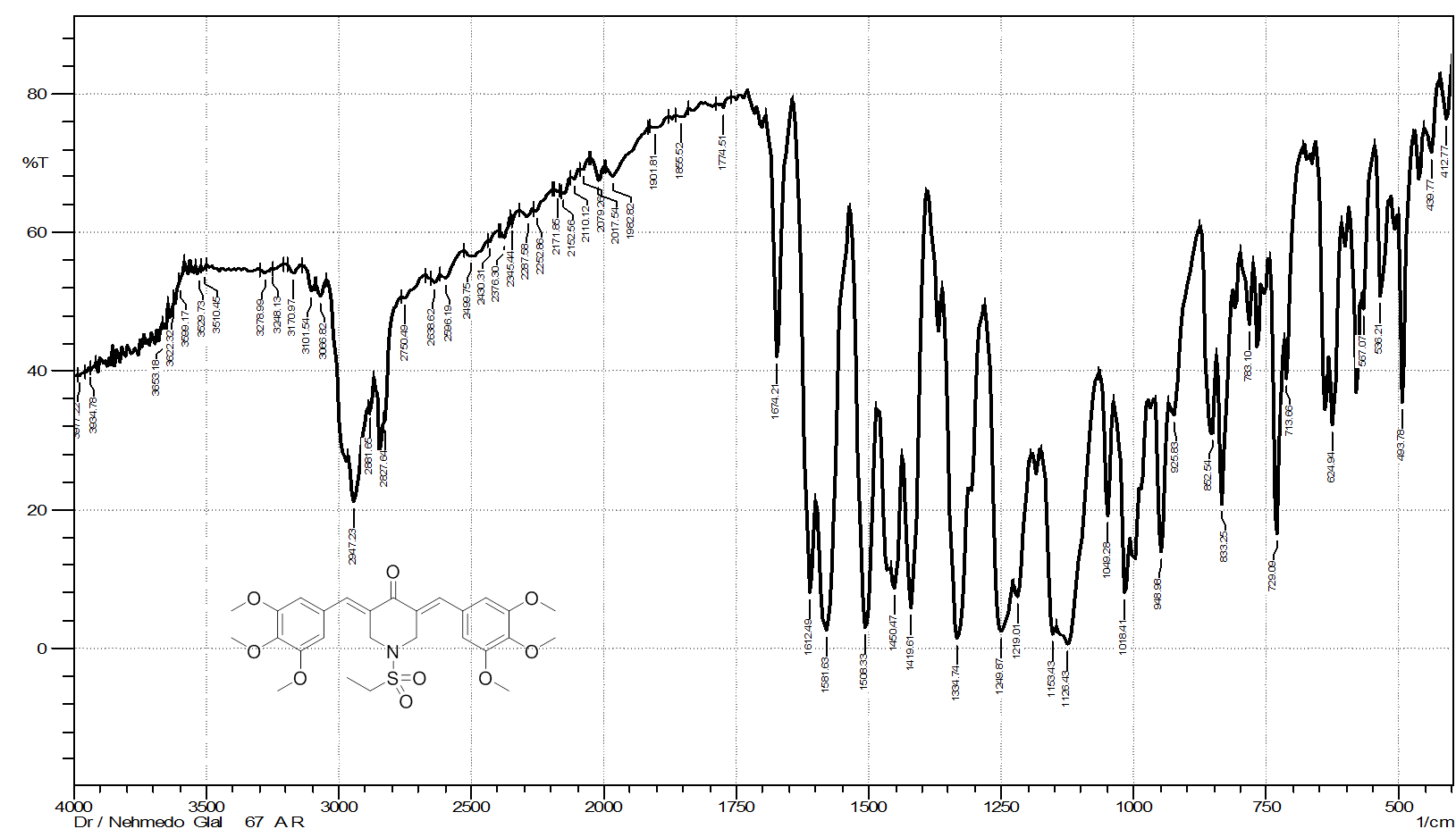


**Fig. S31.** IR spectrum of compound **5ag** (KBr pellet).


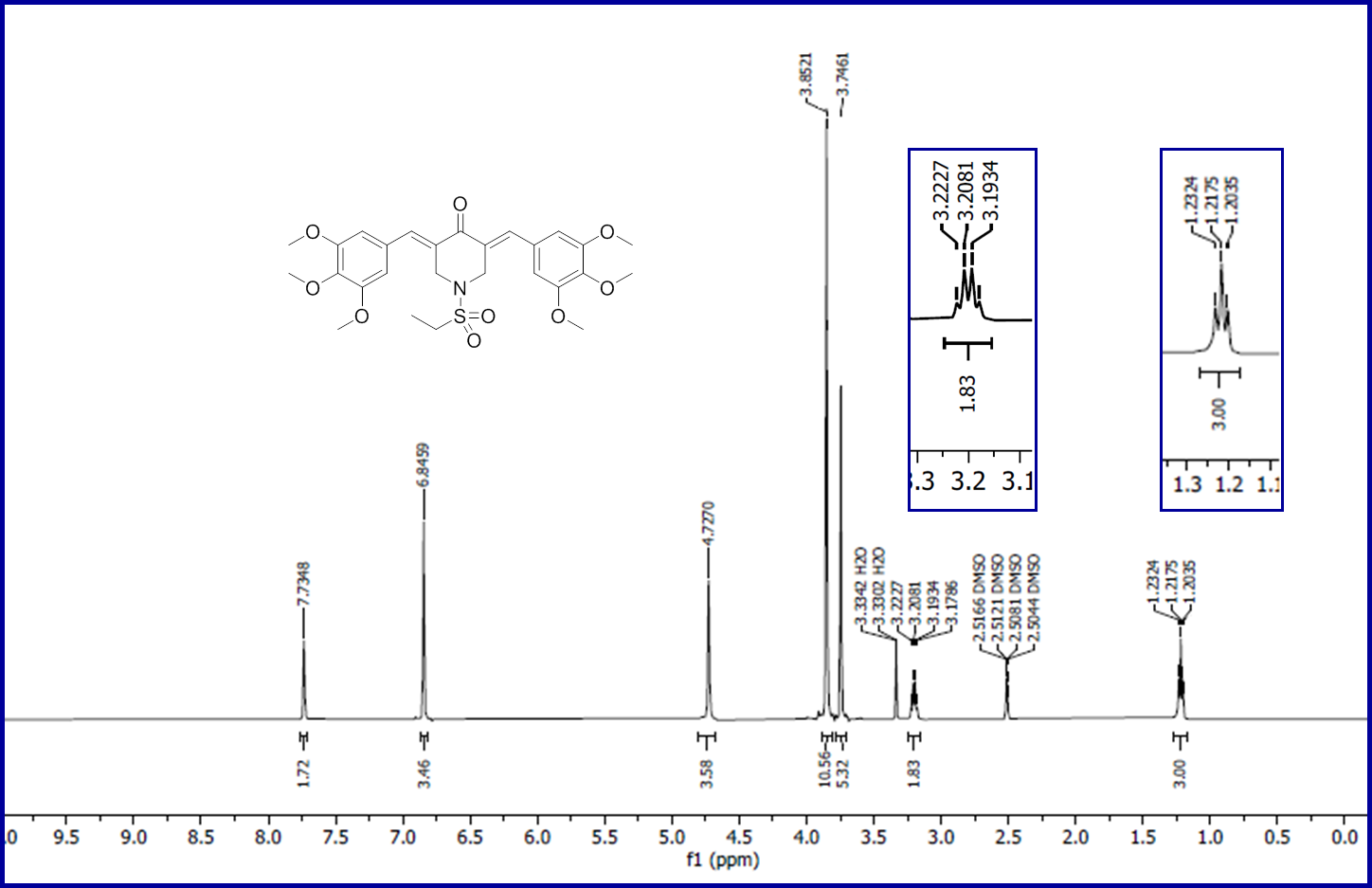


**Fig. S32.** ^1^H-NMR spectrum of compound **5ag** in DMSO-*d_6_*.


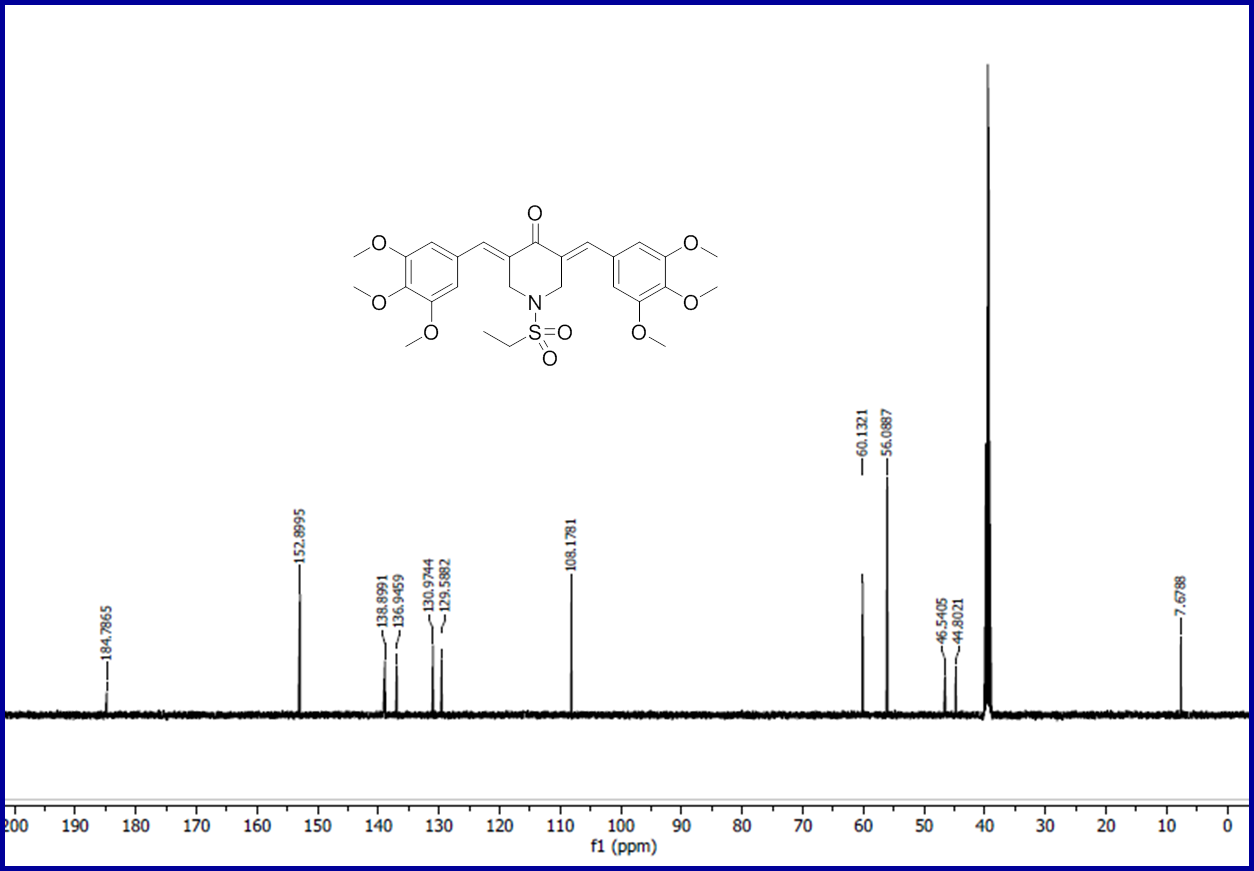


**Fig. S33.** ^13^C-NMR spectrum of compound **5ag** in DMSO-*d_6_*.


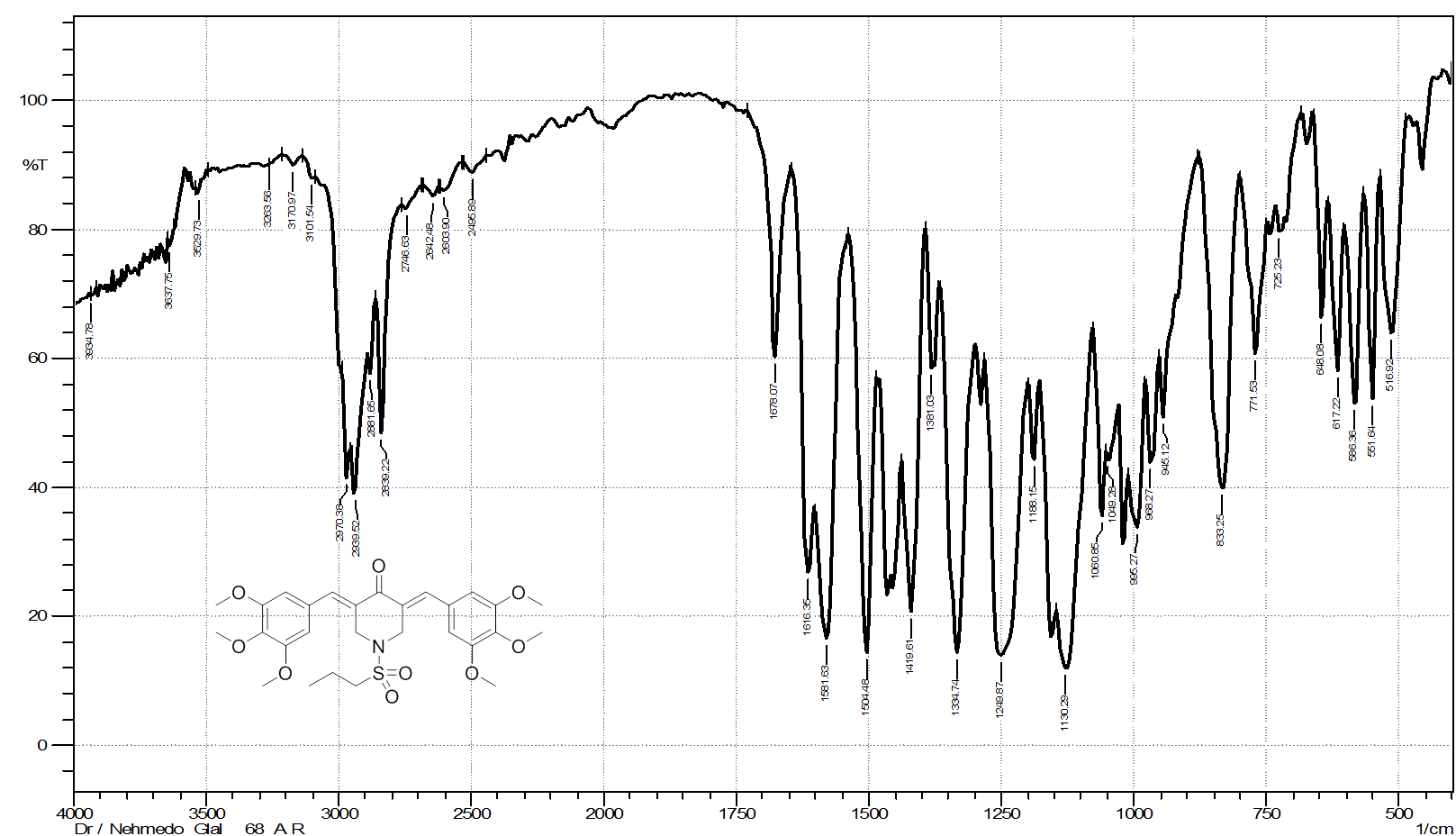


**Fig. S34.** IR spectrum of compound **5ah** (KBr pellet).


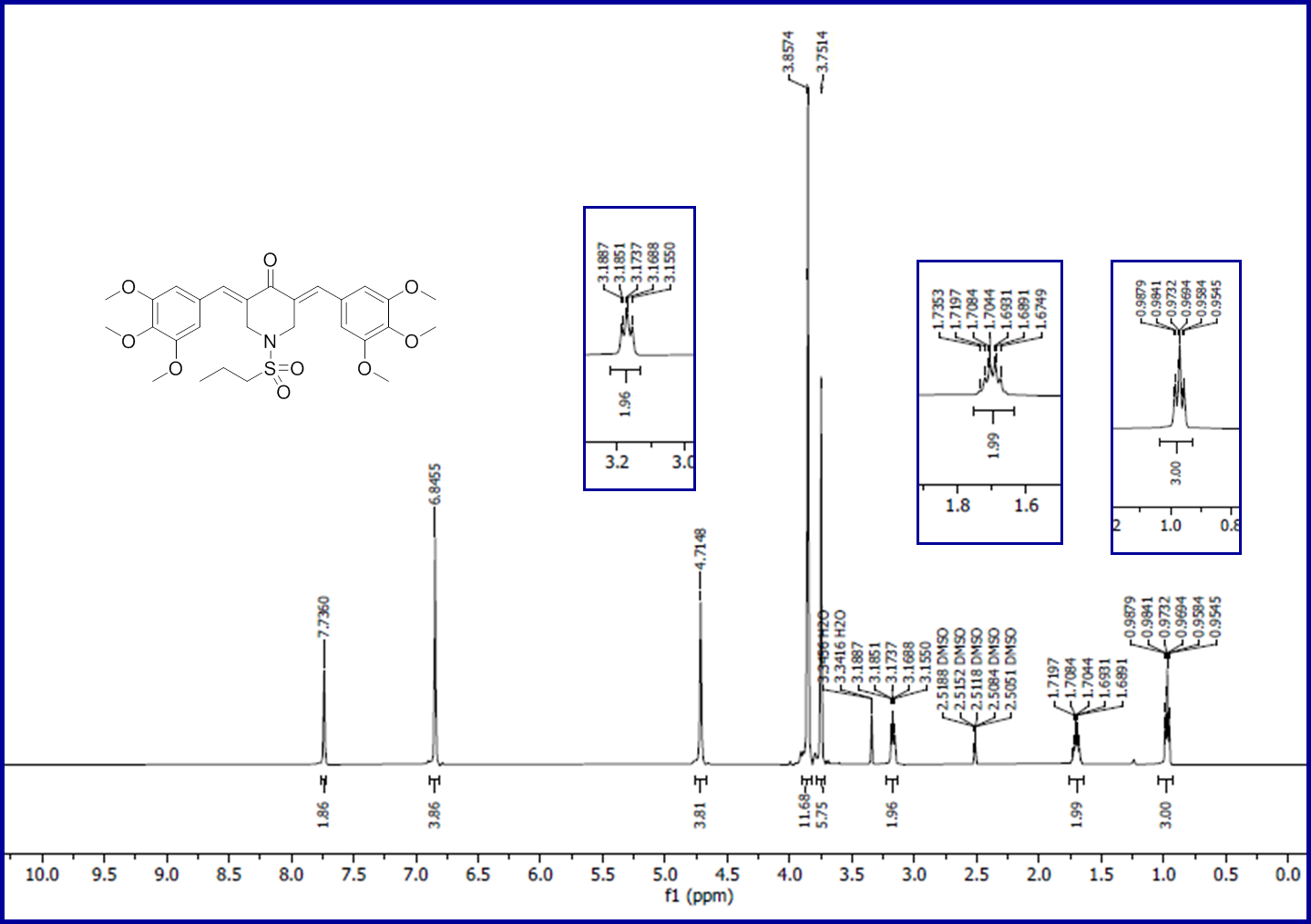


**Fig. S35.** ^1^H-NMR spectrum of compound **5ah** in DMSO-*d_6_*.


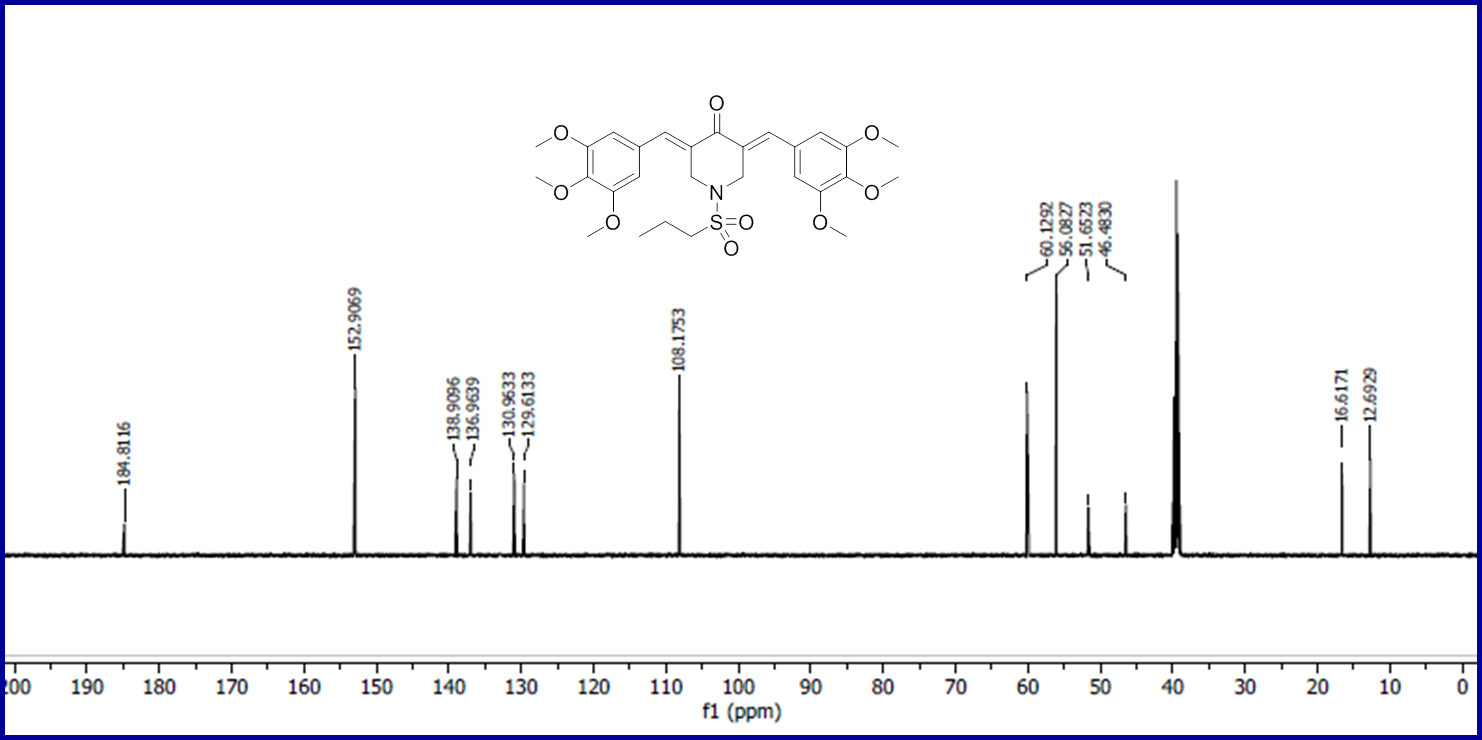


**Fig. S36.** ^13^C-NMR spectrum of compound **5ah** in DMSO-*d_6_*.

**Fig. S37.** Western blotting of NQO1 induction by pre-synthesized piperidones **5a‒5v**. Hepa1c1c7 cells were treated for 48h with vehicle (0.1% DMSO) or 10 µM of compounds **5a‒5v**. 4'BF was used as reference NQO1 inducer. Cell lysates were prepared and NQO1 expression was detected as mentioned in the experimental section.

**
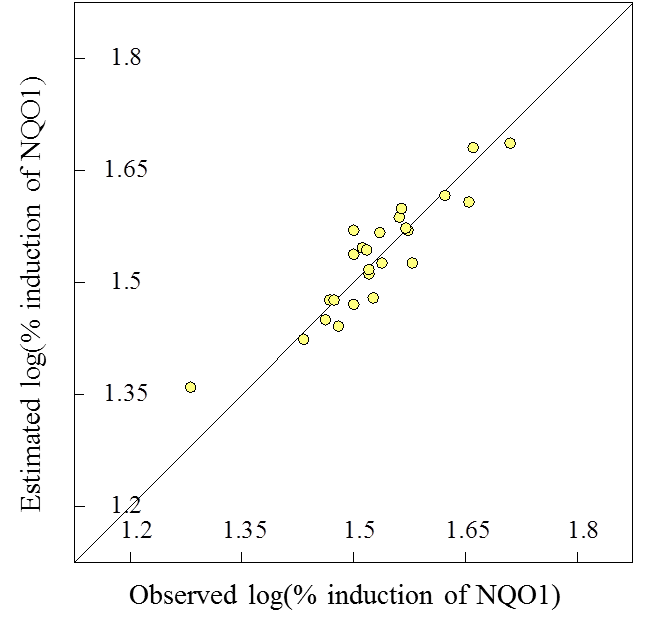
**

**Fig. S38.** QSAR plot representing the observed versus predicted property “log(% induction of NQO1)” for the training set compounds at 10 μM.

(A)

**(B)**


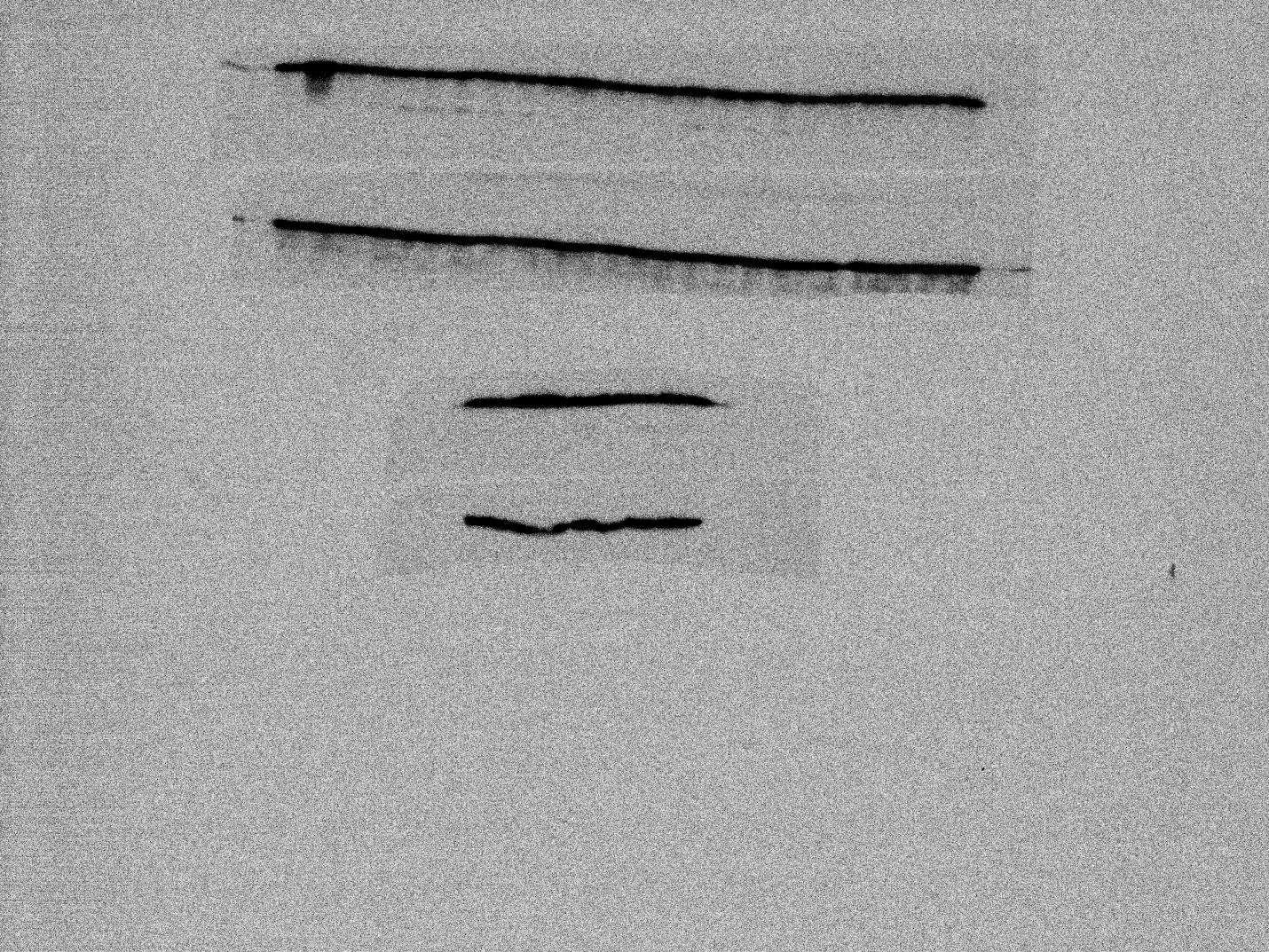


**Fig. S39.** Uncropped western blotting of NQO1 induction by pre-synthesized piperidones **5a‒5v**. Hepa1c1c7 cells were treated for 48h with vehicle (0.1% DMSO) or 10 µM of compounds **5a‒5v**. 4'BF was used as reference NQO1 inducer. Cell lysates were prepared and NQO1 expression was detected as mentioned in the experimental section. Image sensitivity was enhanced in B, compared to auto enhancement A. This was done to reveal the membrane strips in the background as no photos were taken for the membranes before chemiluminescence in this occasion for this experiment.


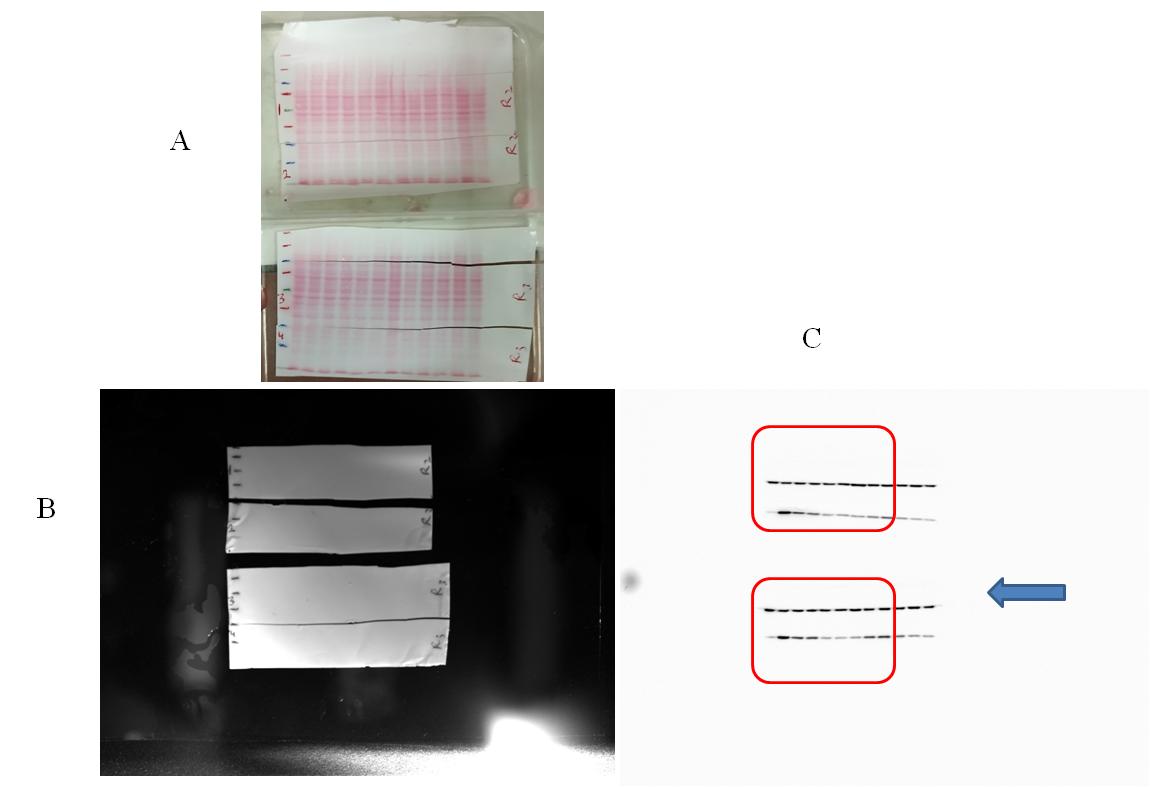


**D**


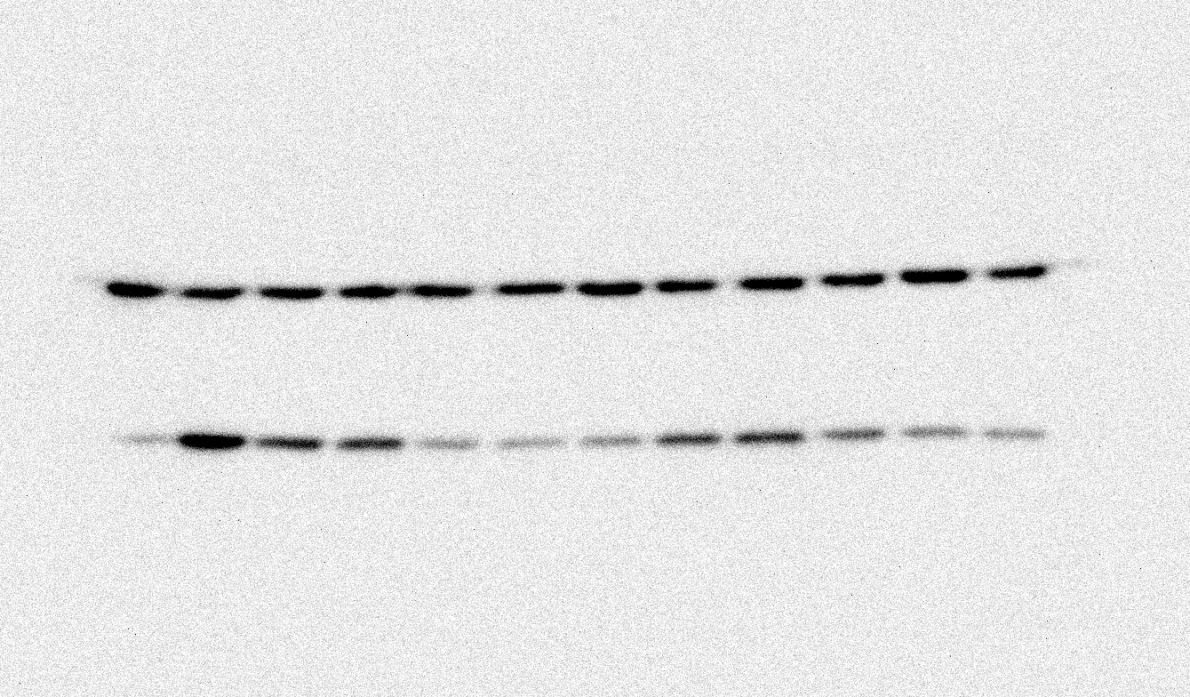


**Ctrl 4‵-BF 5ab 5ac - 5ae - 5w 5x**

**NQO1**

**β-Actin**

**42 kDa**

**30 kDa**

**Fig. S40.** Supplementary images of Figure 3. (A) Colored mobile camera photo of the Ponceau stained membranes of each replica showing the cut strips before blocking step. (B) Imager photos of membrane strips before chemiluminescence. Chemiluminescence image of the replica (2 replicas) of Figure 3. Blue arrow refers to the crude image source of Figure 3. Red shapes indicate the samples involved in the present study. (D) Uncropped western blotting of NQO1 induction by the synthesized piperidones **5w**, **5x**, **5ab**, **5ac** and **5ae** exhibited in the manuscript. Hepa1c1c7 cells were treated for 48h with vehicle (0.1% DMSO) or 10 µM of the tested compounds. 4'-BF was used as reference NQO1 inducer. Cell lysates were prepared and NQO1 expression was detected as mentioned in the experimental section.


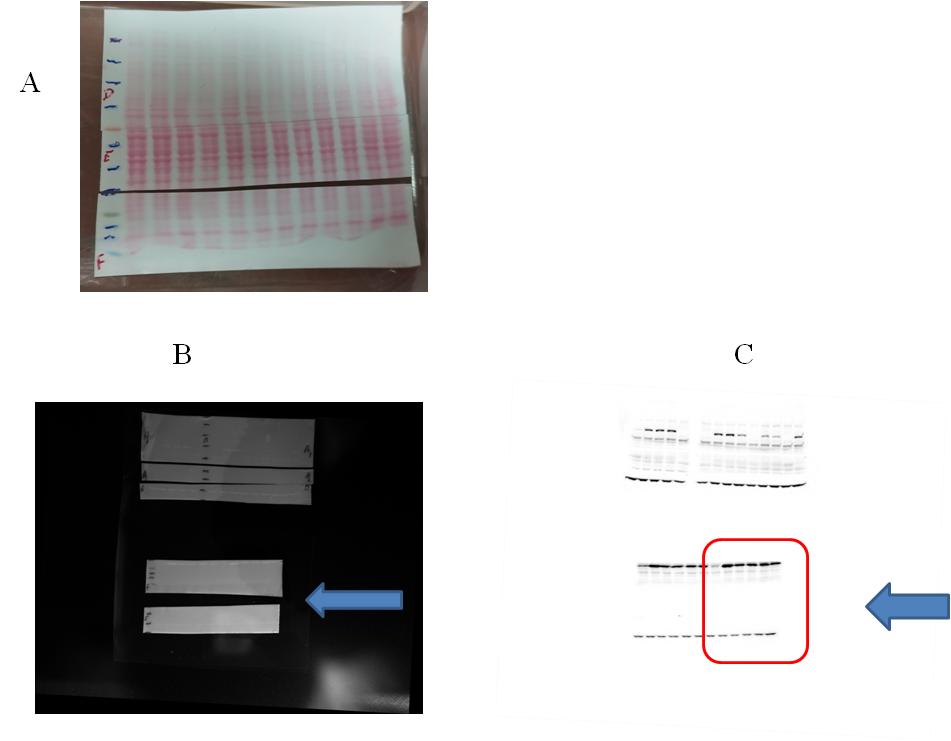


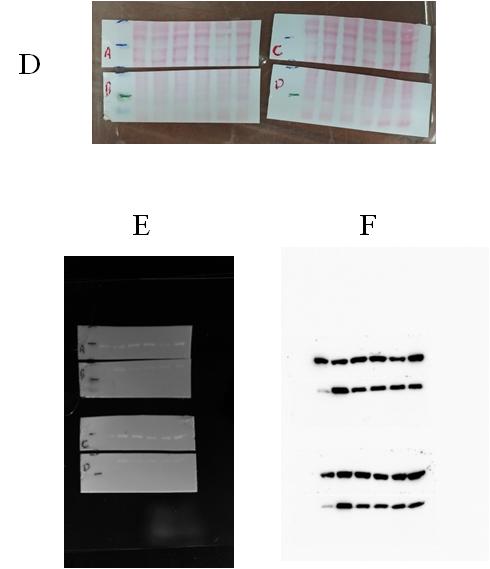


G

**Fig. S41.** Supplementary images of Figure 4. (A) Colored mobile camera photo of the Ponceau S- stained membranes of REPLICA 1 & REPLICA 2 showing the cut strips before blocking step. (B) Imager photos of membrane strips OF REPLICA 1 & REPLICA 2 before chemiluminescence. (C) Chemiluminescence image of the REPLICA 1 & REPLICA 2 of Figure 4, Blue arrow refers to the crude image source of Figure 4. (D) Colored mobile camera photo of the Ponceau stained membranes of REPLICA 3 & REPLICA 4 showing the cut strips before blocking step. (E) Imager photos of membrane strips OF REPLICA 3 & REPLICA 4 before chemiluminescence. (F) Chemiluminescence image of the REPLICA 3 & REPLICA 4 of Figure 4. (D) Uncropped western blotting showing NQO1 induction by 2.5 and 5 µM of compounds **5ab** and **5ac** mentioned in the manuscript as Figure 4.


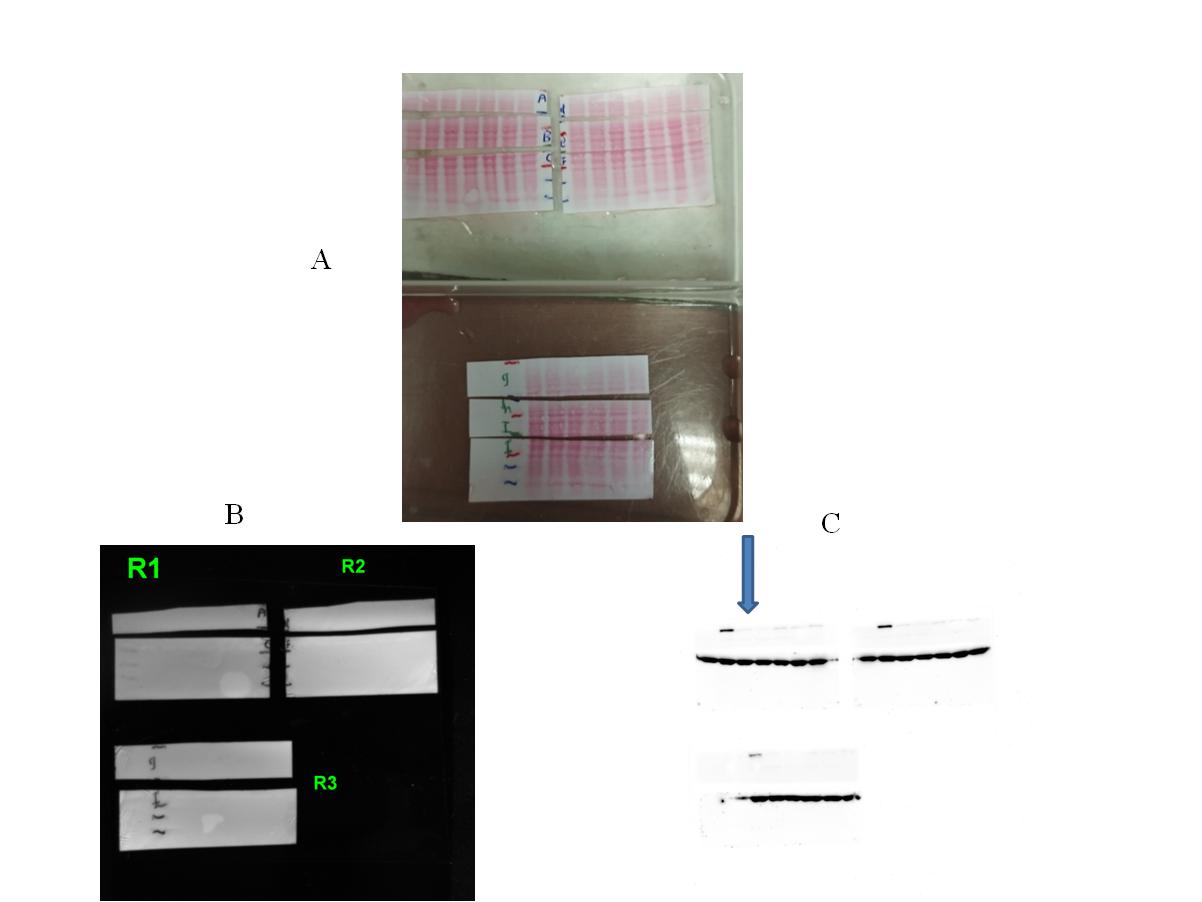


D

**Fig. S42.** Supplementary images of Figure 7. (A) Colored mobile camera photo of the Ponceau stained membranes of REPLICA 1, REPLICA 2 & REPLICA 3 showing the cut strips before blocking step. (B) Imager photos of membrane strips REPLICA 1, REPLICA 2 & REPLICA 3 before chemiluminescence. (C) Chemiluminescence image of REPLICA 1, REPLICA 2 & REPLICA 3 of Figure 7, Blue arrow refers to the crude image source of Figure 7. All replicas contain samples that are essential for the analysis. (D) Uncropped western blot showing concentration-dependent inhibition of LPS-induced iNOS expression by **5ab** and **5ac** in RAW264.7 macrophages mentioned in Figure 7 of manuscript.
